# Liver cancer burden attributable to high body mass index and high fasting plasma glucose in BRICS countries (1990–2021): a Global Burden of Disease 2021 analysis with projections to 2050

**DOI:** 10.3389/fonc.2025.1627282

**Published:** 2025-11-07

**Authors:** Jianya Cai, Yanhong Lan, Ying Wang, Hongwei Cheng, Shuangta Xu

**Affiliations:** 1 Department of Surgery, Quanzhou Medical College, Quanzhou, China; 2 Department of Surgery, The Second Affiliated Hospital of Fujian Medical University, Quanzhou, China; 3 Zhuhai UM Science & Technology Research Institute, University of Macau, Macau, Macao SAR, China

**Keywords:** liver cancer, high BMI, HFPG, burden of disease, projections

## Abstract

**Background:**

With the escalating prevalence of obesity and diabetes, high body mass index (BMI) and high fasting plasma glucose (HFPG) have emerged as increasingly significant risk factors for liver cancer worldwide. This study evaluates the burden of liver cancer attributable to high BMI and HFPG in BRICS countries from 1990 to 2021 and projects future trends to 2050, drawing upon data from the Global Burden of Disease (GBD) Study 2021.

**Method:**

Data on liver cancer burden, stratified by sex and age, were retrieved from the GBD database. Trends were assessed using estimated annual percentage change (EAPC) with 95% confidence intervals. Autoregressive integrated moving average (ARIMA) and exponential smoothing (ES) models were employed to generate future projections.

**Result:**

In 2021, South Africa exhibited the highest liver cancer mortality rate attributable to high BMI, whereas India recorded the most rapid growth. Between 1990 to 2021, mortality and disability-adjusted life years (DALYs) associated with high BMI-related liver cancer increased substantially in China, particularly among older populations. The decomposition analysis revealed that epidemiological change was the dominant driver behind the rising burden in both China and India, with population growth being a secondary yet substantial factor. Epidemiological transitions accounted for the predominant rise in mortality and DALYs in China and India. Forecasts indicate persistent increases in liver cancer mortality and DALYs attributable to high BMI and HFPG through 2050.

**Conclusion:**

The liver cancer burden attributable to high BMI and HFPG is anticipated to escalate across BRICS countries. Enhanced efforts in prevention, early screening, and comprehensive management of obesity, diabetes, and metabolic disorders are indispensable to mitigate the projected impact.

## 1 Introduction

Liver cancer, recognized as one of the principal contributors to cancer-related mortality worldwide, has become a major public health concern owing to its dynamic disease burden and the increasing relevance of risk factor management. According to World Health Organisation data, approximately 865,000 new cases of liver cancer and 757,948 related deaths were reported globally in 2022, placing it as the third most prevalent cause of cancer-related mortality. The mortality rate of liver cancer is particularly pronounced in countries undergoing socioeconomic transition (1). These patterns are largely attributable to lifestyle modifications that have precipitated the rising prevalence of metabolic disorders including obesity and diabetes, both of which are closely linked to the onset and progression of liver cancer (2, 3). High body mass index (BMI) and high fasting plasma glucose (HFPG), central features of metabolic syndrome, have been demonstrated to facilitate liver cancer development through multiple mechanisms, including metabolic dysfunction-associated fatty liver disease (MAFLD) and insulin resistance (4). This pathological cascade is especially evident in emerging economies experiencing rapid nutritional and lifestyle transitions.

As major developing economies, the BRICS countries (Brazil, Russia, India, China, and South Africa) have undergone rapid economic expansion but simultaneously confront substantial public health challenges. Evidence indicates that a pronounced trend of rising obesity prevalence is emerging within BRICS nations (5). As a principal determinant of metabolic syndrome, obesity, together with associated disturbances in glucose and lipid metabolism, is regarded as a contributor to liver cancer development in this region (6, 7). These metabolic disturbances are implicated in advancing the progression of MAFLD and in intensifying the inflammatory microenvironment of the liver (8, 9). The regional concentration of this risk pattern implies that the burden of metabolic-associated liver cancer may evolve along a distinctive trend within BRICS countries. Nevertheless, the majority of existing research has centered on traditional risk factors, particularly viral hepatitis (10, 11), leaving a marked gap in systematic evaluations of metabolic-related liver cancer burden, especially with respect to cross-country comparisons, long-term trend assessments, and projections of future trends.

Drawing upon data from the 2021 Global Burden of Disease (GBD) Study, this study seeks to systematically evaluate the changes in liver cancer burden attributable to high BMI and HFPG in BRICS countries between 1990 and 2021, and to forecast the burden scenario for 2025. With vaccination programs against viral hepatitis having effectively curtailed this traditional risk factor (12, 13), the contribution of metabolic determinants to liver cancer has become increasingly evident. In contrast to viral hepatitis-related liver cancer, metabolic-associated liver cancer constitutes a “silent epidemic”, marked by insidious onset, frequent diagnosis at advanced stages, and a profound deficit in public awareness. Therefore, through an examination of the spatiotemporal heterogeneity of these metabolic risks, this study establishes an evidence-based framework for refining cancer prevention strategies and rectifying the existing imbalance in disease control priorities. The findings are anticipated to provide essential reference points for public health policy development in emerging economies.

## 2 Method

### 2.1 Data source

The GBD 2021 study (https://vizhub.healthdata.org/gbd-results/) systematically compiles and analyses up-to-date global disease burden data on 371 diseases and injuries, while also estimating the associations between 88 risk factors and corresponding health outcomes (14, 15). Data on the attributable fractions of liver cancer burden, estimated through the GBD comparative risk assessment framework that assigns disease burden to high BMI and HFPG, were obtained from the GBD 2021 database. High BMI was defined as a BMI ≥ 25 kg/m², and HFPG was defined as a fasting plasma glucose level exceeding 86.4-97.2 mg/dL (16).

### 2.2 Descriptive analysis

The distributional characteristics of liver cancer burden attributable to high BMI and HFPG in BRICS countries, stratified by sex and age, were examined for the years 1990 and 2021. In the GBD 2021 study, the Age-Standardized Rate (ASR per 100,000) was calculated by first summing the products of the age-specific rate (*ai*) and its corresponding weight (*wi*) for each age group, and then dividing this sum by the total standard population weight. The formula for calculating the ASR was defined as follows:


$$ASR=\frac{\sum_{i=1}^{A}a_{i}w_{i}}{\sum_{i=1}^{A}w_{i}}\times 100,000$$

Where i denotes the ith age group, $a_{i}$ represents the age-specific rate, and $w_{i}$ indicates the population size (or weight) in the corresponding age groups of the selected reference standard population (17). In this study, ASRs were expressed per 100,000 population.

Uncertainty intervals (UIs) were derived from the 2.5th and 97.5th percentiles of a 1000-draw distribution for each metric (18). All statistical analyses and graphical visualizations were performed using R software (version 4.1.0). Key R packages included data.table (v1.16.2) and openxlsx (v4.2.7.1) for data reading and storage; dplyr (v1.1.4), tidyr (v1.3.1), and purrr (v1.0.2) for data cleaning and wrangling; and ggplot2 (v3.3.5), ggmap (v4.4.0), and forecast (v8.23.0) for data visualization. Throughout the analysis, a P-value of less than 0.05 was considered to indicate statistical significance.

### 2.3 Trend analysis

The estimated annual percentage change (EAPC) was employed to evaluate the long-term trends in age-standardized mortality rate (ASMR), age-standardized disability-adjusted life years rate (ASDR), age-standardized years lived with disability rate (ASYR), and age-standardized years of life lost (YLLs) rate between 1990 and 2021.The formula for calculating EAPC was expressed as follows:


y=α+βx+ϵ

$$EAPC=\left(e^{\beta }-1\right)\times 100\%$$

Where y denotes ln(ASR), x represents the calendar year, and β corresponds to slope derived from the linear regression of the natural logarithm of the ASR on the year (19).

### 2.4 Decomposition analysis

Decomposition analysis is applied to determine the additive contributions of differences in population factors to variations in overall values between two populations (20). In this study, the contributions of age structure, population growth, and epidemiological shifts to changes in deaths, disability-adjusted life years (DALYs), years lived with disability (YLDs), and YLLs from liver cancer attributable to high BMI and HFPG in BRICS countries between 1990 and 2021 were quantified.

### 2.5 Forecasting analysis

In this study, projections of liver cancer burden attributable to high BMI and HFPG were generated using the exponential smoothing (ES) model and the autoregressive integrated moving average (ARIMA) model. The ARIMA model is particularly suited to capturing long-term trends and seasonal fluctuations in data, whereas the ES model emphasizes recent observations, thereby offering a complementary perspective on potential future developments (21).

## 3 Result

### 3.1 Overall burden in BRICS

**Figure 1:**
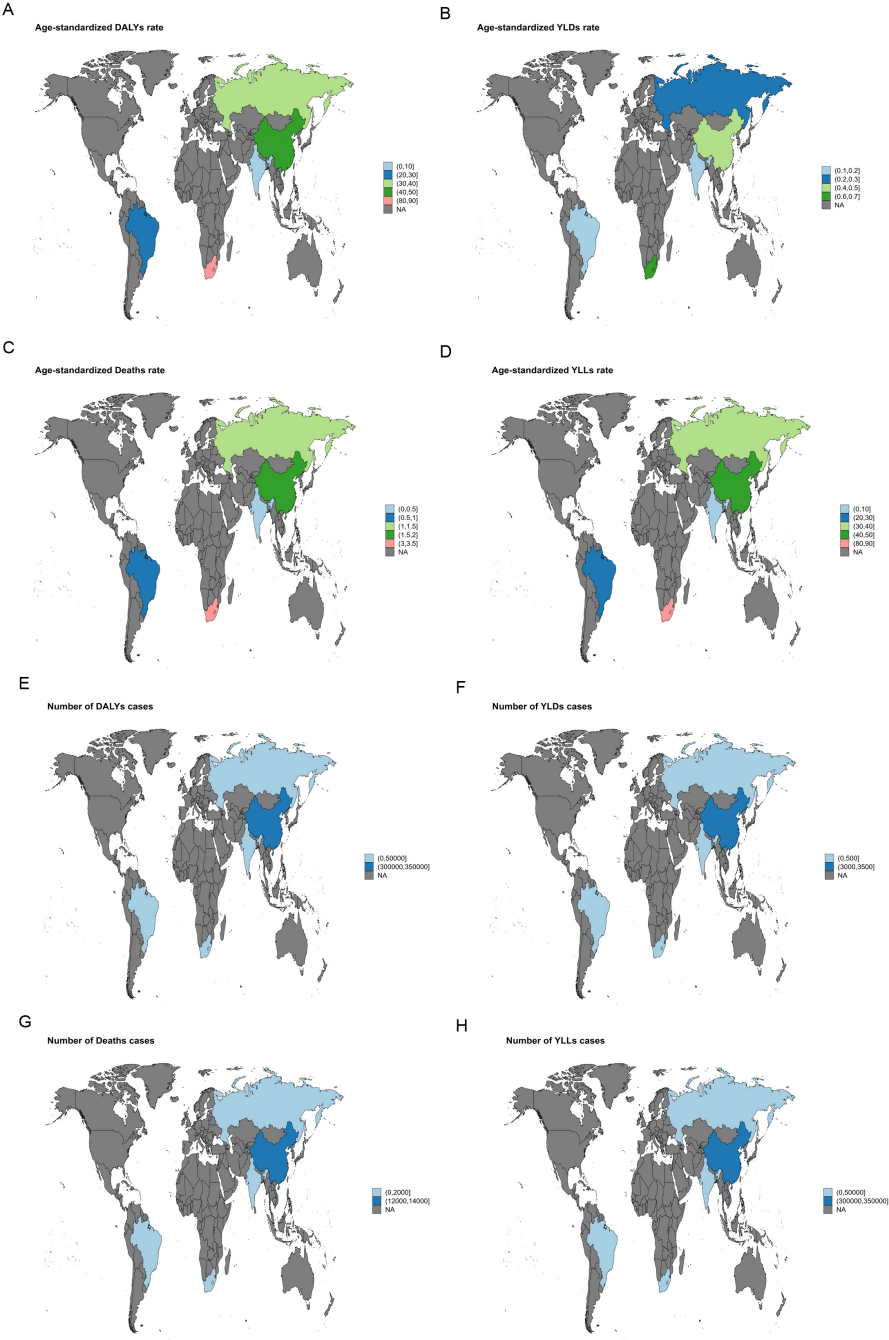
World map illustrating the disease burden of liver cancer attributable to high BMI in BRICS countries in 2021. **(A)** ASDR. **(B)** ASYR. **(C)** ASMR. **(D)** Age-standardized YLL rate. **(E)** Number of DALY cases. **(F)** Number of YLD cases. **(G)** Number of deaths. **(H)** Number of YLL cases.

**Figure 2:**
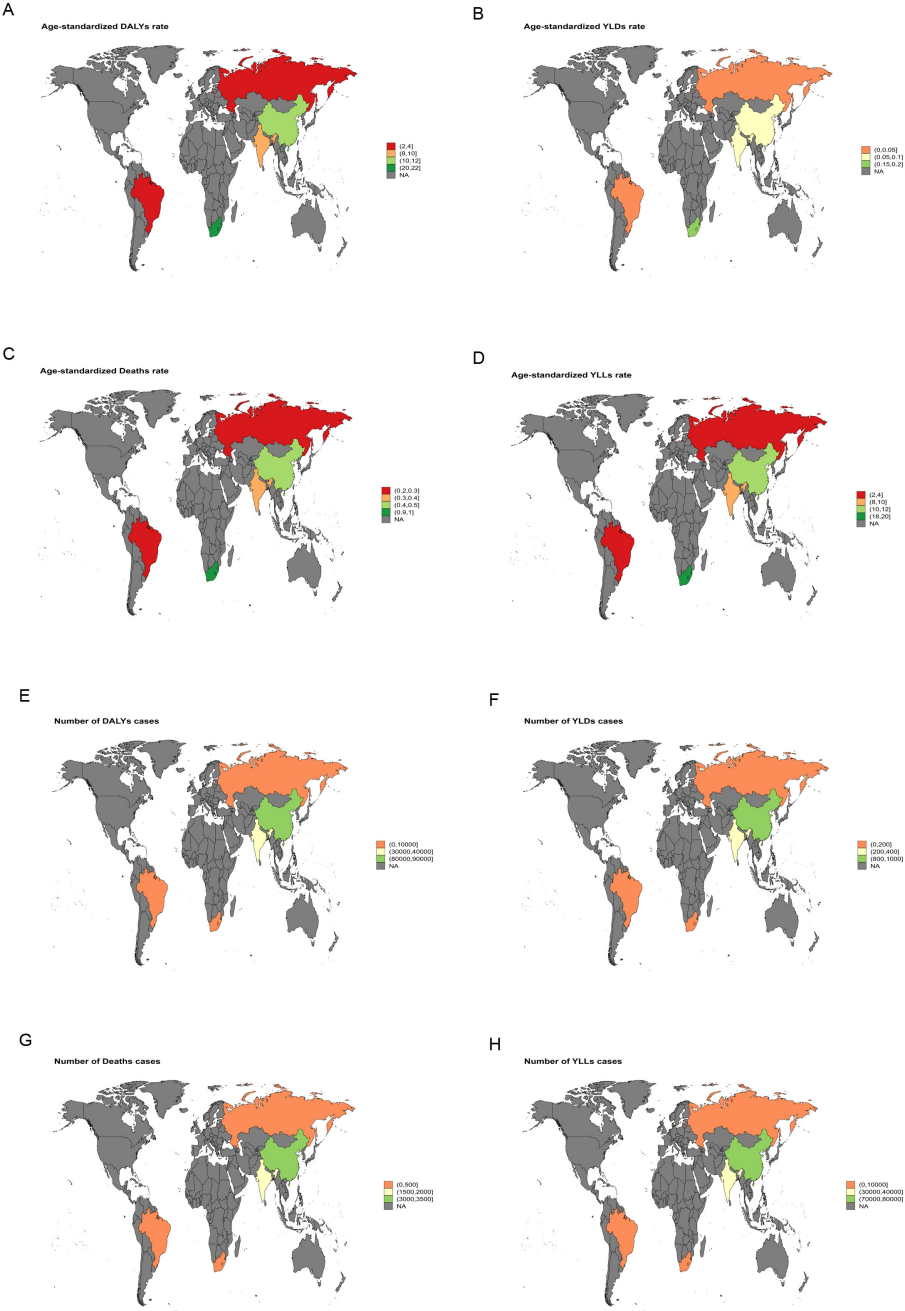
World map illustrating the disease burden of liver cancer attributable to HFPG in BRICS countries in 2021. **(A)** ASDR. **(B)** ASYR. **(C)** ASMR. **(D)** Age-standardized YLLs rate. **(E)** Number of DALY cases. **(F)** Number of YLD cases. **(G)** Number of deaths. **(H)** Number of YLL cases.

In 2021, South Africa recorded the highest ASMR for liver cancer attributable to high BMI [3.29 (95% UI: 1.4-5.6)], and the highest ASDR was likewise reported in South Africa [84.81 (95% UI: 36.21-143.71)] (**Figure 1**). For liver cancer associated with HFPG, the highest ASMR was also noted in South Africa [1.00 (95% UI: 0.09-2.12)], together with the highest ASDR [20.07 (95% UI: 1.87-42.64)] (**Figure 2**).

**Figure 3:**
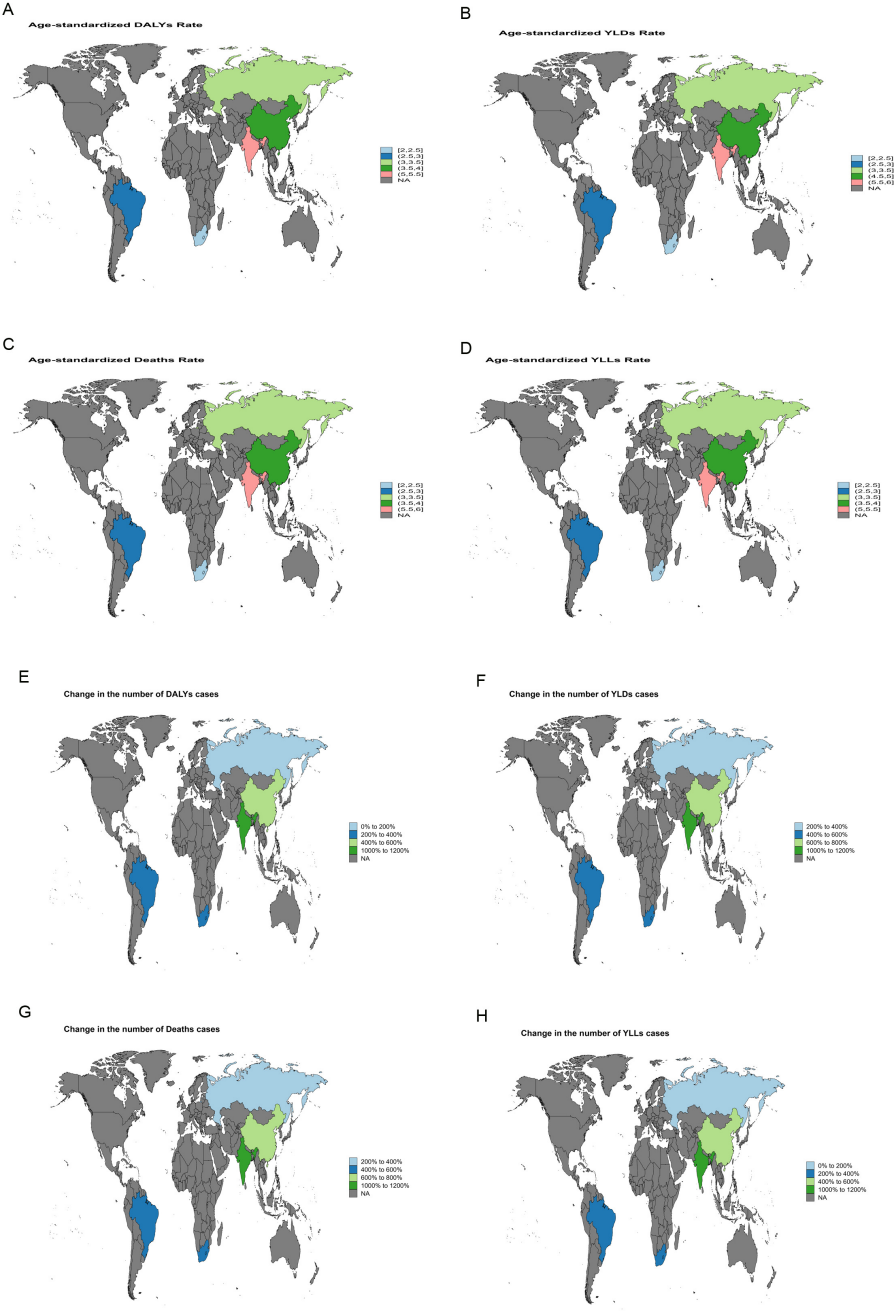
World map depicting the EAPC of liver cancer burden attributable to high BMI in BRICS countries from 1990 to 2021. **(A)** EAPC of the ASDR. **(B)** EAPC of the ASYR. **(C)** EAPC of the ASMR. **(D)** EAPC of the age-standardized YLL rate. **(E)** EAPC in the number of DALYs. **(F)** EAPC in the number of YLDs. **(G)** EAPC in the number of deaths. **(H)** EAPC in the number of YLLs.

**Figure 4:**
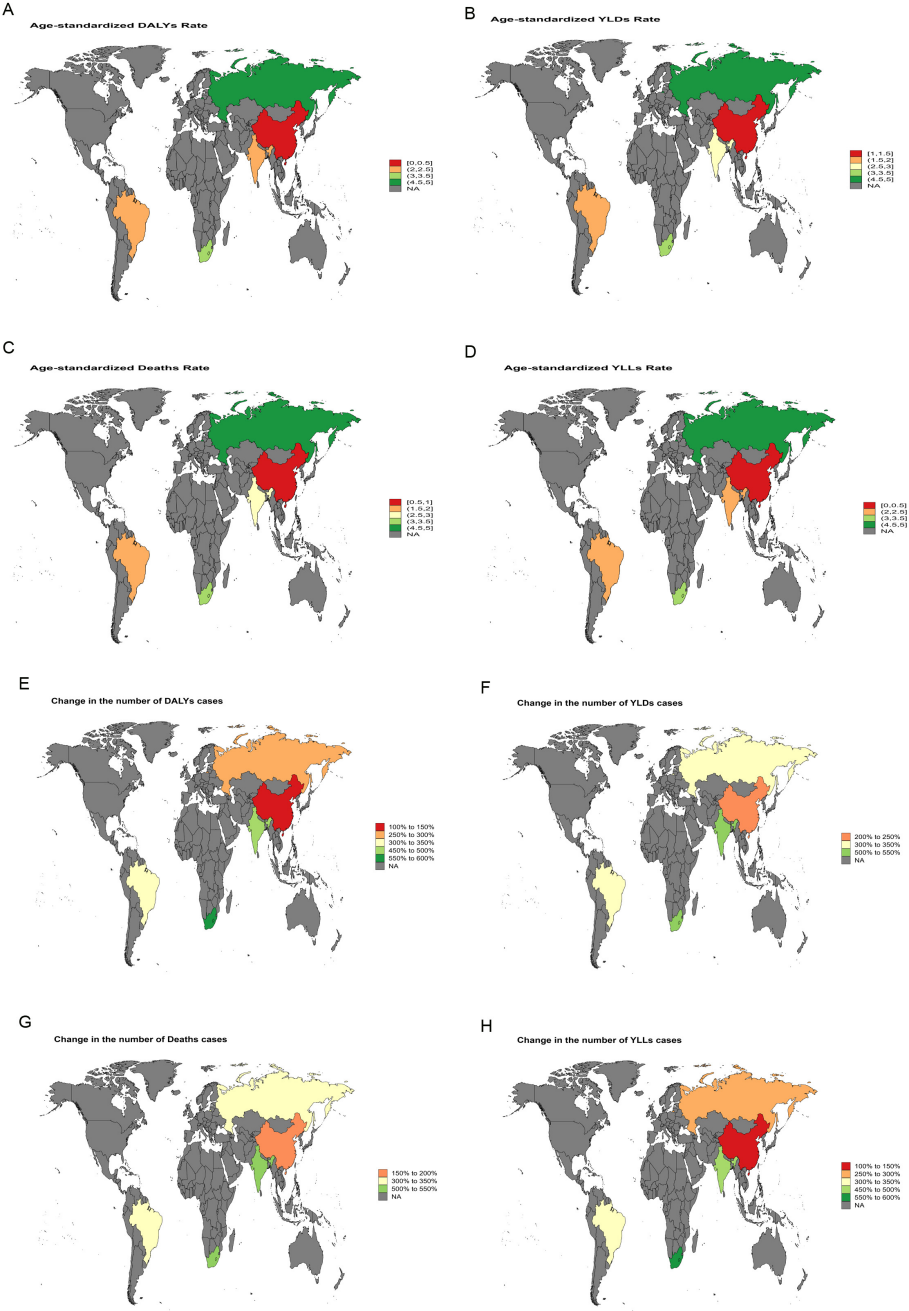
World map depicting the EAPC of liver cancer burden attributable to HFPG in BRICS countries from 1990 to 2021. **(A)** EAPC of the ASDR. **(B)** EAPC of the ASYR. **(C)** EAPC of the ASMR. **(D)** EAPC of the age-standardized YLL rate. **(E)** EAPC in the number of DALYs. **(F)** EAPC in the number of YLDs. **(G)** EAPC in the number of deaths. **(H)** EAPC in the number of YLLs.

Between 1990 and 2021, India exhibited the largest increase in ASMR for liver cancer attributable to high BMI, with an EAPC of 5.64 [95% confidence intervals (95%CI): 5.49-5.78]. The most rapid growth in ASDR was likewise recorded in India, with an EAPC of 5.45 (95% *CI*: 5.37-5.53) (**Figure 3**). For liver cancer associated with HFPG, the steepest rise in ASMR was observed in the Russian Federation, with an EAPC of 4.99 (95% *CI*: 4.55-5.42), and the fastest growth in ASDR was also noted in the Russian Federation, with an EAPC of 4.62 (95% *CI*: 4.20-5.05) (**Figure 4**).

### 3.2 Sex-specific burden

In 2021, the ASMR for liver cancer attributable to high BMI was higher in males than in females across BRICS countries, with a similar pattern observed for the ASDR (**Supplementary Figure S1**). For liver cancer associated with HFPG, the ASMR was likewise higher in males than in females, with the ASDR showing the same pattern (**Supplementary Figure S2**).

Between 1990 and 2021, in China, the ASMR for liver cancer attributable to high BMI in females increased from 0.37 (95% UI: 0.15-0.66) in 1990 to 1.19 (95% UI: 0.46-2.19) in 2021, with an EAPC of 4.17 (95% *CI*: 4.02-4.33). Among males, the ASMR rose from 0.73 (95% UI: 0.3-1.23) in 1990 to 2.19 (95% UI: 0.83-4.15) in 2021, corresponding to an EAPC of 3.81 (95% *CI*: 3.66-3.95). The ASDR for females increased from 9.85 (95% UI: 4.01-17.38) in 1990 to 29.67 (95% UI: 11.57-55.35) in 2021, with an EAPC of 3.92 (95% *CI*: 3.79-4.05). For males, the ASDR rose from 22.32 (95% UI: 9.06-37.64) in 1990 to 64.92 (95% UI: 24.41-124.21) in 2021, reflecting an EAPC of 3.65 (95% *CI*: 3.50-3.81) (**Supplementary Figures S3**-**S7**). For liver cancer attributable to HFPG in China, the ASMR for females declined slightly from 0.45 (95% UI: 0.05-0.99) in 1990 to 0.43 (95% UI: 0.04-0.93) in 2021, with an EAPC of 0.58 (95% *CI*: 0.28-0.89). In males the ASMR increased from 0.48 (95% UI: 0.05-1.03) in 1990 to 0.55 (95% UI: 0.06-1.23) in 2021, with an EAPC of 1.08 (95% *CI*: 0.76-1.40). The ASDR for females declined from 10.77 (95% UI: 1.12-23.72) in 1990 to 9.41 (95% UI: 0.96-20.13) in 2021, with an EAPC of 0.17 (95% *CI*: -0.10-0.45). Among males, the ASDR increased from 11.74 (95% UI: 1.11-25.41) in 1990 to 12.74 (95% UI: 1.35-28.48) in 2021, with an EAPC of 0.78 (95% *CI*: 0.50-1.07) (**Supplementary Figures S8**-**S12**). Detailed results for other BRICS countries are presented in **Tables 1**, **2**, **Supplementary Tables S1**, **S2**.

**Table 1:** The deaths and ASMR of liver cancer attributable to high BMI in BRICS countries in 1990 and 2021.

	1990		2021		EAPC (95% *CI*)
	Number (95% UI)	ASR (95% UI)	Number (95% UI)	ASR (95% UI)	
**Brazil**	135.13 (52.65-245.66)	0.45 (0.18-0.82)	726.21 (288.44-1252.69)	0.86 (0.34-1.48)	2.87 (2.65-3.09)
Sex					
Female	73.89 (27.81-136.59)	0.47 (0.18-0.88)	303.61 (120.13-517.22)	0.65 (0.26-1.11)	1.59 (1.37-1.81)
Male	61.24 (23.16-116.31)	0.43 (0.16-0.81)	422.6 (166.47-736.81)	1.11 (0.44-1.94)	3.78 (3.48-4.09)
Age					
40–44 years	6.24 (2.41-11.51)	0.08 (0.03-0.15)	17.26 (6.93-28.77)	0.1 (0.04-0.17)	1.27 (1.08-1.46)
45–49 years	9.8 (3.74-18.2)	0.16 (0.06-0.3)	28.99 (11.7-50.49)	0.2 (0.08-0.36)	1.43 (1.08-1.78)
50–54 years	15.34 (6.28-27.54)	0.3 (0.12-0.53)	57.53 (23.76-95.14)	0.45 (0.18-0.74)	1.96 (1.63-2.3)
55–59 years	17.98 (7.32-33.15)	0.42 (0.17-0.78)	91.36 (35.9-155.96)	0.78 (0.31-1.33)	2.61 (2.29-2.92)
60–64 years	22.28 (8.69-39.9)	0.62 (0.24-1.11)	115.81 (46.82-200.39)	1.18 (0.48-2.04)	2.77 (2.51-3.03)
65–69 years	21.29 (8.07-38.13)	0.79 (0.3-1.42)	121.55 (48.74-209.45)	1.58 (0.63-2.72)	2.78 (2.54-3.01)
70–74 years	18.24 (7.01-32.92)	0.96 (0.37-1.74)	106.96 (42.67-183.5)	1.86 (0.74-3.19)	2.84 (2.55-3.13)
75–79 years	13.96 (5.48-25.92)	1.09 (0.43-2.02)	79.05 (31.15-137.86)	2.09 (0.82-3.64)	2.72 (2.45-3)
80–84 years	6.36 (2.33-11.59)	0.94 (0.35-1.72)	56.08 (20.97-99.08)	2.28 (0.85-4.03)	3.57 (3.29-3.86)
85–89 years	2.68 (0.98-5.03)	0.92 (0.33-1.72)	32.32 (12.36-57.77)	2.49 (0.95-4.46)	3.81 (3.56-4.06)
90–94 years	0.78 (0.27-1.43)	0.93 (0.33-1.72)	14.65 (5.65-25.85)	2.62 (1.01-4.63)	3.61 (3.39-3.83)
95+ years	0.19 (0.06-0.35)	0.99 (0.35-1.87)	4.64 (1.79-8.43)	2.19 (0.84-3.97)	2.89 (2.52-3.27)
**China**	1612.87 (663.59-2682.13)	0.55 (0.23-0.92)	12087.08 (4602.01-21726.27)	1.68 (0.64-3.03)	3.98 (3.84-4.13)
Sex					
Female	527.79 (215.47-929.22)	0.37 (0.15-0.66)	4510.17 (1749.34-8278.5)	1.19 (0.46-2.19)	4.17 (4.02-4.33)
Male	1085.08 (441.04-1832.61)	0.73 (0.3-1.23)	7576.91 (2853.05-14384.84)	2.19 (0.83-4.15)	3.81 (3.66-3.95)
Age					
40–44 years	164.36 (68.33-272.81)	0.24 (0.1-0.41)	579.09 (219.62-1096.3)	0.63 (0.24-1.2)	2.74 (2.49-2.98)
45–49 years	190.3 (77.24-309.95)	0.37 (0.15-0.6)	1082.04 (412.95-2043.22)	0.98 (0.37-1.85)	3.36 (2.99-3.72)
50–54 years	217.59 (88.22-366.37)	0.46 (0.18-0.77)	1550.23 (575.75-2921.76)	1.28 (0.48-2.42)	3.79 (3.55-4.02)
55–59 years	239.78 (97.11-407.52)	0.55 (0.22-0.94)	1580.15 (613.76-2967.47)	1.44 (0.56-2.7)	3.31 (3.09-3.52)
60–64 years	242.95 (98.5-404.13)	0.69 (0.28-1.14)	1536.28 (570.66-2765.55)	2.1 (0.78-3.79)	4.03 (3.83-4.23)
65–69 years	225.32 (92.92-365.34)	0.83 (0.34-1.34)	2058.33 (785.35-3528.65)	2.68 (1.02-4.6)	4.21 (3.91-4.51)
70–74 years	170.49 (71.93-288.65)	0.91 (0.38-1.53)	1506.72 (572.35-2632.71)	2.83 (1.07-4.94)	3.61 (3.44-3.78)
75–79 years	99.37 (41.86-161.08)	0.87 (0.37-1.42)	984.77 (391.52-1650.5)	2.97 (1.18-4.98)	4.11 (3.93-4.28)
80–84 years	38.37 (17.13-65.29)	0.72 (0.32-1.23)	651.57 (250.6-1133.23)	3.29 (1.27-5.73)	6.24 (5.82-6.66)
85–89 years	20.14 (8.56-33.99)	1.19 (0.51-2.02)	394.78 (148.17-698.05)	4.14 (1.56-7.33)	4.64 (4.23-5.04)
90–94 years	3.95 (1.69-6.56)	1.29 (0.55-2.14)	141.71 (53.4-251.39)	4.83 (1.82-8.57)	4.39 (3.95-4.83)
95+ years	0.25 (0.11-0.43)	0.61 (0.26-1.05)	21.42 (7.88-37.43)	3.35 (1.23-5.86)	6.38 (5.78-6.99)
**India**	114.63 (42.94-210.99)	0.07 (0.03-0.13)	1414.44 (535.77-2503.29)	0.34 (0.13-0.61)	5.64 (5.49-5.78)
Sex					
Female	41.34 (13.9-85.36)	0.05 (0.02-0.11)	531.61 (185.49-1010.67)	0.26 (0.09-0.49)	5.46 (5.36-5.55)
Male	73.3 (27.06-146.09)	0.09 (0.03-0.17)	882.83 (317.95-1655.03)	0.44 (0.16-0.82)	5.68 (5.58-5.78)
Age					
40–44 years	6.87 (2.56-12.21)	0.02 (0.01-0.03)	64.54 (23.72-109.45)	0.07 (0.03-0.12)	5.2 (4.96-5.44)
45–49 years	11.96 (4.16-22.07)	0.03 (0.01-0.06)	105.12 (40.18-187.93)	0.13 (0.05-0.24)	4.71 (4.57-4.84)
50–54 years	15.39 (6-29.66)	0.05 (0.02-0.1)	152.37 (57.86-271.75)	0.23 (0.09-0.4)	5.1 (4.92-5.29)
55–59 years	21.26 (7.7-38.24)	0.08 (0.03-0.15)	232.31 (87.7-415.16)	0.42 (0.16-0.74)	5.53 (5.39-5.66)
60–64 years	18.83 (7.46-34.82)	0.09 (0.04-0.17)	228.07 (86.07-402.57)	0.48 (0.18-0.85)	5.71 (5.59-5.82)
65–69 years	16.1 (6.12-29.89)	0.12 (0.04-0.22)	235.1 (88.02-406.48)	0.62 (0.23-1.07)	5.71 (5.59-5.84)
70–74 years	12.3 (4.3-22.39)	0.14 (0.05-0.26)	181.92 (66.53-320.84)	0.67 (0.24-1.18)	5.4 (5.21-5.58)
75–79 years	8.52 (3.3-15.45)	0.17 (0.06-0.3)	131.82 (54.67-234.39)	0.78 (0.32-1.38)	5.44 (5.27-5.61)
80–84 years	2.64 (1.05-4.8)	0.09 (0.04-0.17)	53.54 (19.59-99.12)	0.55 (0.2-1.02)	6.38 (5.99-6.76)
85–89 years	0.66 (0.25-1.25)	0.07 (0.02-0.13)	22.05 (8.59-41.19)	0.53 (0.2-0.98)	7.63 (6.91-8.36)
90–94 years	0.1 (0.04-0.18)	0.04 (0.02-0.07)	6.84 (2.58-12.98)	0.54 (0.2-1.03)	9.38 (8.17-10.6)
95+ years	0.01 (0-0.01)	0.01 (0-0.02)	0.76 (0.27-1.42)	0.28 (0.1-0.52)	10.64 (9.21-12.08)
**Russian Federation**	352.32 (142.07-588.86)	0.58 (0.23-0.96)	1093.31 (439.19-1880.16)	1.36 (0.54-2.33)	3.52 (3.19-3.84)
Sex					
Female	200.07 (80.92-343.31)	0.5 (0.2-0.86)	529.41 (214.9-882.92)	1.03 (0.42-1.71)	3.01 (2.51-3.51)
Male	152.26 (59.32-256.48)	0.72 (0.28-1.21)	563.9 (223.02-1012.27)	1.84 (0.73-3.3)	3.57 (3.22-3.93)
Age					
40–44 years	7.86 (3.08-12.91)	0.08 (0.03-0.14)	19.01 (7.53-31.63)	0.17 (0.07-0.29)	3.21 (2.54-3.89)
45–49 years	11.02 (4.32-18.05)	0.17 (0.07-0.27)	35.25 (13.77-60.1)	0.35 (0.14-0.6)	3.01 (2.45-3.58)
50–54 years	35.4 (13.64-59.77)	0.34 (0.13-0.57)	58.75 (23.01-103.67)	0.67 (0.26-1.17)	3.09 (2.59-3.58)
55–59 years	45.9 (18.79-74.79)	0.58 (0.24-0.94)	120.53 (48.19-205.65)	1.28 (0.51-2.18)	2.95 (2.5-3.41)
60–64 years	76.35 (31.09-128.4)	0.87 (0.35-1.46)	184.35 (74.75-322.95)	1.79 (0.72-3.13)	2.96 (2.55-3.37)
65–69 years	58.92 (23.9-99.28)	1.15 (0.47-1.93)	220.92 (88.81-376.84)	2.62 (1.05-4.46)	3.12 (2.69-3.55)
70–74 years	41.58 (16.66-67.94)	1.21 (0.48-1.98)	149.76 (59.72-253.55)	2.34 (0.93-3.96)	3.15 (2.63-3.68)
75–79 years	43.76 (18.01-73.96)	1.26 (0.52-2.14)	93.84 (38.35-159.82)	3.71 (1.51-6.31)	4.18 (3.74-4.61)
80–84 years	21.09 (8.39-36)	1.16 (0.46-1.97)	124.02 (50.31-214.51)	3.89 (1.58-6.73)	4.61 (4.13-5.09)
85–89 years	7.96 (3.17-13.53)	1.09 (0.44-1.86)	51.47 (20.56-89.13)	3.72 (1.49-6.44)	4.92 (4.49-5.36)
90–94 years	2.08 (0.84-3.5)	1.24 (0.5-2.08)	30.86 (12.34-54.31)	4.7 (1.88-8.28)	4.77 (4.36-5.17)
95+ years	0.42 (0.17-0.71)	1.23 (0.48-2.09)	4.56 (1.85-8.02)	3.3 (1.33-5.79)	2.9 (2.33-3.48)
**South Africa**	98.05 (34.38-185.83)	1.42 (0.5-2.7)	502.54 (214.09-853.22)	3.29 (1.4-5.6)	2.16 (1.73-2.59)
Sex					
Female	63.54 (21.74-124.28)	1.62 (0.55-3.18)	236.29 (103.34-389.38)	2.68 (1.17-4.44)	1.38 (1.03-1.72)
Male	34.51 (9.64-78.5)	1.14 (0.32-2.61)	266.26 (103.51-479.6)	4.18 (1.61-7.59)	3.26 (2.35-4.19)
Age					
40–44 years	8.63 (3.15-15.45)	0.49 (0.18-0.89)	29.86 (12.99-51.16)	0.79 (0.34-1.35)	1.01 (0.42-1.61)
45–49 years	9.36 (3.48-17.03)	0.68 (0.25-1.23)	36.41 (15.54-61.86)	1.16 (0.5-1.98)	1.57 (1.24-1.9)
50–54 years	11.62 (4.07-21.31)	1 (0.35-1.84)	56.12 (23.34-92.55)	2.15 (0.9-3.55)	2.18 (1.62-2.74)
55–59 years	13.68 (4.86-26.63)	1.45 (0.52-2.82)	72.83 (32.35-124.02)	3.17 (1.41-5.4)	2.04 (1.38-2.7)
60–64 years	12.7 (4.35-23.97)	1.57 (0.54-2.95)	71.72 (30.96-120.77)	3.72 (1.61-6.26)	2.27 (1.65-2.89)
65–69 years	13.1 (4.4-25.62)	2.16 (0.72-4.22)	75.15 (32.31-126.44)	5.17 (2.22-8.7)	2.15 (1.5-2.8)
70–74 years	10.4 (3.45-20.37)	2.31 (0.77-4.53)	55.21 (23.28-95.07)	5.57 (2.35-9.6)	2.28 (1.61-2.95)
75–79 years	7.16 (2.5-13.54)	2.74 (0.96-5.18)	41.76 (17.22-71.68)	6.88 (2.84-11.82)	2.18 (1.54-2.83)
80–84 years	6.13 (2.26-11.77)	3.5 (1.29-6.71)	34.6 (14.22-60.29)	9.07 (3.73-15.8)	2.43 (1.84-3.02)
85–89 years	3.69 (1.3-7.13)	4.04 (1.42-7.8)	20.31 (8.42-34.36)	11.66 (4.83-19.72)	2.64 (2.02-3.26)
90–94 years	1.32 (0.47-2.52)	4.4 (1.56-8.41)	7.27 (2.93-12.75)	14.08 (5.68-24.68)	3.04 (2.5-3.59)
95+ years	0.26 (0.09-0.49)	3.98 (1.4-7.41)	1.31 (0.54-2.28)	16.68 (6.92-29.15)	4.11 (3.77-4.45)

**Table 2:** The DALYs and age-standardized DALYs rate (ASDR) of liver cancer attributable to high BMI in BRICS countries in 1990 and 2021.

	1990		2021		EAPC (95% *CI*)
	Number (95% UI)	ASR (95% UI)	Number (95% UI)	ASR (95% UI)	
**Brazil**	3802.29 (1489.67-6911.35)	11.9 (4.65-21.62)	18390.37 (7345.51-31561.06)	21.32 (8.51-36.61)	2.6 (2.42-2.79)
Sex					
Female	2000.15 (761.59-3664.32)	12.04 (4.57-22.11)	7259.18 (2887.88-12297.32)	15.55 (6.18-26.34)	1.39 (1.18-1.61)
Male	1802.14 (682.96-3424.58)	11.66 (4.42-22.16)	11131.19 (4404.88-19285.66)	28.05 (11.09-48.68)	3.48 (3.15-3.81)
Age					
40–44 years	300.26 (115.83-554.18)	3.88 (1.49-7.15)	830.79 (333.5-1384.16)	5.03 (2.02-8.38)	1.26 (1.07-1.45)
45–49 years	423.66 (161.8-786.58)	6.91 (2.64-12.82)	1254.61 (506.33-2184.23)	8.83 (3.56-15.37)	1.43 (1.08-1.77)
50–54 years	590.43 (241.87-1058.72)	11.45 (4.69-20.53)	2213.14 (913.55-3661.59)	17.17 (7.09-28.4)	1.96 (1.62-2.3)
55–59 years	606.52 (246.72-1118.15)	14.21 (5.78-26.19)	3086.67 (1212.24-5266.96)	26.34 (10.35-44.95)	2.61 (2.29-2.92)
60–64 years	648.5 (252.9-1160.75)	18.12 (7.07-32.43)	3375.39 (1363.48-5846.73)	34.35 (13.88-59.51)	2.78 (2.52-3.04)
65–69 years	522.95 (198.29-936.1)	19.52 (7.4-34.94)	2987.36 (1197.16-5149.46)	38.78 (15.54-66.85)	2.78 (2.54-3.01)
70–74 years	368.37 (141.41-664.57)	19.42 (7.45-35.03)	2163.05 (862.57-3709.61)	37.66 (15.02-64.58)	2.84 (2.55-3.12)
75–79 years	225.73 (88.64-419.31)	17.55 (6.89-32.6)	1278.64 (503.82-2229.93)	33.73 (13.29-58.83)	2.72 (2.44-2.99)
80–84 years	80.48 (29.48-146.95)	11.96 (4.38-21.84)	709.88 (264.92-1255.03)	28.85 (10.77-51)	3.56 (3.27-3.85)
85–89 years	27.07 (9.82-50.68)	9.26 (3.36-17.35)	325.01 (124.26-579.74)	25.08 (9.59-44.73)	3.79 (3.54-4.04)
90–94 years	6.8 (2.39-12.49)	8.18 (2.88-15.03)	128.21 (49.27-225.46)	22.97 (8.83-40.39)	3.6 (3.38-3.82)
95+ years	1.52 (0.53-2.87)	8.15 (2.83-15.38)	37.62 (14.42-68.16)	17.71 (6.79-32.09)	2.82 (2.43-3.21)
**China**	50938.51 (20869.96-84729.71)	16.31 (6.69-27.13)	342661.04 (130068.63-623835.41)	47.35 (17.97-86.32)	3.71 (3.6-3.83)
Sex					
Female	14747.16 (5999.68-26043.4)	9.85 (4.01-17.38)	112331.18 (43837.99-209253.84)	29.67 (11.57-55.35)	3.92 (3.79-4.05)
Male	36191.35 (14666.03-61042.93)	22.32 (9.06-37.64)	230329.86 (86508.64-441438.03)	64.92 (24.41-124.21)	3.65 (3.5-3.81)
Age					
40–44 years	7910.99 (3288.64-13123.89)	11.79 (4.9-19.56)	27874.24 (10576.6-52777.62)	30.45 (11.55-57.66)	2.73 (2.49-2.97)
45–49 years	8221.4 (3339.49-13412.71)	15.93 (6.47-25.98)	46676.2 (17794.75-87994.3)	42.31 (16.13-79.76)	3.35 (2.99-3.72)
50–54 years	8346.5 (3379.88-14052.98)	17.49 (7.08-29.45)	59579.33 (22107.38-112169.25)	49.3 (18.29-92.81)	3.8 (3.56-4.03)
55–59 years	8093.67 (3275.59-13759.12)	18.66 (7.55-31.73)	53466.72 (20751.05-100420.8)	48.63 (18.87-91.34)	3.31 (3.09-3.53)
60–64 years	7070.12 (2866.11-11755.82)	20.01 (8.11-33.27)	44638.89 (16562.54-80278.2)	61.14 (22.69-109.96)	4.03 (3.83-4.23)
65–69 years	5517.7 (2272.12-8943.69)	20.22 (8.33-32.78)	50411.17 (19216.91-86489.04)	65.72 (25.05-112.76)	4.22 (3.92-4.53)
70–74 years	3439.33 (1450.53-5835.95)	18.28 (7.71-31.01)	30460.68 (11563.21-53153.39)	57.15 (21.7-99.73)	3.61 (3.44-3.78)
75–79 years	1610.37 (677.79-2608.59)	14.15 (5.96-22.92)	15917.35 (6311.62-26675.3)	48.06 (19.06-80.54)	4.09 (3.91-4.26)
80–84 years	487.41 (217.39-830.11)	9.2 (4.1-15.67)	8235.21 (3156.32-14317.13)	41.61 (15.95-72.34)	6.22 (5.8-6.64)
85–89 years	204.28 (86.75-345.72)	12.11 (5.14-20.49)	3981.66 (1495.72-7044.27)	41.8 (15.7-73.95)	4.63 (4.22-5.03)
90–94 years	34.68 (14.78-57.54)	11.3 (4.82-18.75)	1242.75 (467.23-2206.96)	42.39 (15.94-75.27)	4.39 (3.95-4.83)
95+ years	2.06 (0.88-3.58)	5.09 (2.17-8.84)	176.83 (65.31-309.16)	27.67 (10.22-48.37)	6.34 (5.73-6.96)
**India**	3528.9 (1318.32-6499.31)	1.98 (0.74-3.64)	40555.55 (15327.37-71680.54)	9.45 (3.57-16.71)	5.45 (5.37-5.53)
Sex					
Female	1237.51 (418.27-2521.94)	1.48 (0.5-3.03)	14444.8 (5048.39-27177.82)	6.7 (2.34-12.64)	5.23 (5.14-5.31)
Male	2291.4 (844.03-4578.23)	2.44 (0.9-4.86)	26110.76 (9410.81-48733.46)	12.24 (4.41-22.9)	5.61 (5.49-5.73)
Age					
40–44 years	330.14 (123.2-587.52)	0.76 (0.28-1.35)	3105.86 (1141.21-5270.31)	3.4 (1.25-5.77)	5.2 (4.96-5.44)
45–49 years	516.95 (180.06-953.9)	1.44 (0.5-2.65)	4548.39 (1737.88-8134.77)	5.77 (2.2-10.32)	4.71 (4.57-4.84)
50–54 years	591.89 (230.82-1140.38)	2.01 (0.78-3.87)	5857.97 (2225.08-10453.95)	8.69 (3.3-15.52)	5.1 (4.92-5.29)
55–59 years	717.21 (259.76-1290.68)	2.86 (1.04-5.15)	7842.44 (2961.42-14018.14)	14.02 (5.3-25.07)	5.53 (5.39-5.67)
60–64 years	548.43 (217.46-1013.93)	2.75 (1.09-5.08)	6641.14 (2504.49-11729.85)	13.94 (5.26-24.63)	5.71 (5.59-5.82)
65–69 years	395.91 (150.34-733.69)	2.9 (1.1-5.37)	5768.07 (2164.56-9978.13)	15.22 (5.71-26.32)	5.7 (5.58-5.83)
70–74 years	249.18 (86.93-453.57)	2.85 (0.99-5.18)	3683.57 (1344.49-6487.28)	13.55 (4.95-23.86)	5.39 (5.21-5.58)
75–79 years	137.93 (53.58-250.07)	2.68 (1.04-4.86)	2138.58 (886.92-3805.54)	12.63 (5.24-22.47)	5.43 (5.26-5.59)
80–84 years	33.62 (13.33-61.22)	1.19 (0.47-2.17)	680.48 (249.63-1261.56)	6.99 (2.56-12.96)	6.37 (5.99-6.75)
85–89 years	6.74 (2.49-12.69)	0.68 (0.25-1.29)	222.78 (86.84-415.42)	5.32 (2.07-9.91)	7.63 (6.9-8.36)
90–94 years	0.84 (0.34-1.57)	0.34 (0.14-0.64)	59.93 (22.57-113.77)	4.77 (1.79-9.05)	9.38 (8.18-10.6)
95+ years	0.05 (0.02-0.1)	0.11 (0.04-0.2)	6.35 (2.28-11.82)	2.31 (0.83-4.31)	10.64 (9.2-12.1)
**Russian Federation**	9344 (3754.12-15583.35)	15.03 (6.03-25.03)	26494.39 (10614.84-45527.97)	33.54 (13.42-57.62)	3.28 (2.95-3.62)
Sex					
Female	4976.42 (2017.29-8532.42)	12.87 (5.21-22.05)	11539.95 (4712.48-19124)	23.87 (9.75-39.54)	2.63 (2.13-3.13)
Male	4367.58 (1702.45-7370.92)	18.59 (7.25-31.34)	14954.43 (5873.34-26798.26)	46.75 (18.36-83.68)	3.6 (3.2-4.01)
Age					
40–44 years	379.68 (148.67-624.15)	4.03 (1.58-6.63)	915.27 (362.72-1521.51)	8.38 (3.32-13.93)	3.21 (2.54-3.89)
45–49 years	471.04 (184.74-771.02)	7.1 (2.78-11.62)	1525.89 (596.05-2602.5)	15.2 (5.94-25.93)	3.01 (2.45-3.58)
50–54 years	1363.21 (524.96-2301.3)	12.98 (5-21.91)	2262.53 (885.29-3988.78)	25.61 (10.02-45.15)	3.09 (2.6-3.57)
55–59 years	1540.37 (629.63-2510.77)	19.31 (7.89-31.47)	4055.95 (1620.67-6920.64)	42.94 (17.16-73.27)	2.95 (2.49-3.41)
60–64 years	2222.06 (904.02-3738.29)	25.28 (10.28-42.52)	5369.2 (2176.18-9394.78)	52.07 (21.1-91.11)	2.98 (2.55-3.4)
65–69 years	1455.62 (589.84-2451.44)	28.33 (11.48-47.71)	5434.72 (2184.77-9258.5)	64.39 (25.88-109.69)	3.12 (2.7-3.54)
70–74 years	836.65 (334.8-1365.98)	24.32 (9.73-39.71)	3032.71 (1209.01-5126.77)	47.31 (18.86-79.98)	3.15 (2.64-3.66)
75–79 years	705.54 (290.59-1189.8)	20.37 (8.39-34.35)	1505.51 (615.01-2564.4)	59.44 (24.28-101.25)	4.14 (3.72-4.57)
80–84 years	267.68 (106.3-457.27)	14.69 (5.83-25.09)	1565.39 (634.46-2708.61)	49.14 (19.92-85.02)	4.61 (4.14-5.09)
85–89 years	80.43 (31.87-136.72)	11.06 (4.38-18.8)	519.84 (207.53-901.88)	37.57 (15-65.18)	4.91 (4.46-5.35)
90–94 years	18.21 (7.32-30.69)	10.82 (4.35-18.23)	270.11 (108.06-474.41)	41.16 (16.47-72.29)	4.77 (4.36-5.17)
95+ years	3.51 (1.38-5.94)	10.28 (4.03-17.4)	37.28 (15.09-65.17)	26.93 (10.9-47.09)	2.83 (2.26-3.41)
**South Africa**	2876.83 (1014.82-5407.79)	38.79 (13.65-73.18)	14099.8 (6035.52-23867.91)	84.81 (36.21-143.71)	2.04 (1.66-2.43)
Sex					
Female	1814.52 (628.75-3479.35)	44.13 (15.25-85.02)	6410.16 (2811.78-10511.04)	69 (30.25-113.3)	1.33 (1.04-1.63)
Male	1062.31 (296.71-2401.48)	31.99 (8.92-72.65)	7689.64 (3009.55-13790.32)	106.2 (41.3-191.06)	2.93 (2.02-3.85)
Age					
40–44 years	415.89 (151.82-744.91)	23.83 (8.7-42.69)	1440.3 (626.61-2464.22)	38.11 (16.58-65.2)	1.01 (0.42-1.61)
45–49 years	405.12 (150.97-736.61)	29.24 (10.9-53.16)	1577.46 (673.53-2677.72)	50.44 (21.54-85.63)	1.57 (1.24-1.89)
50–54 years	448.05 (156.83-821.79)	38.7 (13.55-70.98)	2161.44 (898.22-3565.59)	82.91 (34.46-136.78)	2.17 (1.61-2.74)
55–59 years	461.91 (164.16-898.72)	49 (17.41-95.34)	2461.49 (1092.48-4191.59)	107.25 (47.6-182.62)	2.04 (1.38-2.7)
60–64 years	370.12 (126.61-699.42)	45.62 (15.61-86.2)	2091.69 (901.98-3520.89)	108.5 (46.79-182.64)	2.27 (1.65-2.89)
65–69 years	320.81 (107.66-627.36)	52.86 (17.74-103.38)	1850.65 (795.17-3117.62)	127.36 (54.72-214.54)	2.16 (1.51-2.81)
70–74 years	211.95 (70.25-415.89)	47.11 (15.62-92.44)	1121.11 (472.8-1927.48)	113.17 (47.73-194.57)	2.28 (1.61-2.95)
75–79 years	115.09 (40.1-217.36)	44.04 (15.34-83.18)	676.56 (279.16-1161.85)	111.55 (46.03-191.57)	2.19 (1.54-2.83)
80–84 years	77.02 (28.46-147.62)	43.9 (16.22-84.16)	439.31 (180.52-763.65)	115.1 (47.3-200.08)	2.44 (1.84-3.04)
85–89 years	37.16 (13.11-71.89)	40.67 (14.35-78.68)	205.05 (84.8-346.2)	117.71 (48.68-198.73)	2.65 (2.03-3.28)
90–94 years	11.55 (4.09-22.18)	38.5 (13.62-73.94)	63.77 (25.69-111.89)	123.46 (49.74-216.63)	3.05 (2.51-3.59)
95+ years	2.16 (0.76-4.04)	32.85 (11.55-61.27)	10.98 (4.56-19.21)	140.26 (58.29-245.46)	4.14 (3.79-4.49)

### 3.3 Age-specific burden

In 2021, for liver cancer attributable to high BMI, the ASMR and ASDR in most BRICS countries rose with age before declining after a certain threshold. In contrast, in South Africa, both indicators continued to increase with age (**Supplementary Figure S13**). For liver cancer associated with HFPG, a similar age-related pattern was observed across all BRICS countries, with ASMR and ASDR increasing initially and then decreasing after a certain age (**Supplementary Figure S14**).

Between 1990 and 2021, for liver cancer associated with high BMI, the ASMR and ASDR rose across all age group. Notably, the rate of increase was amplified with advancing age, indicating that older age groups experienced faster growth in both ASMR and ASDR. For instance, in China, the steepest rise in ASMR was observed in the 95+ age group (EAPC = 6.38, 95%CI: 5.78-6.99), while the most pronounced increase in ASDR occurred in the same age group (EAPC = 6.34, 95% *CI*: 5.73-6.96) (**Supplementary Figures S15**-**S19**). For liver cancer attributable to HFPG, a comparable trend was identified. In China, the 95+ age group again showed the fastest growth, with ASMR rising at an EAPC of 3.66 (95%CI: 3.15–4.17) and ASDR at an EAPC of 3.63 (95%CI: 3.11-4.15) (**Supplementary Figures S20**-**S24**). Detailed results for other BRICS countries are provided in **Tables 1**, **2**, **Supplementary Tables S1**, **S2**.

### 3.4 Decomposition analysis

**Figure 5:**
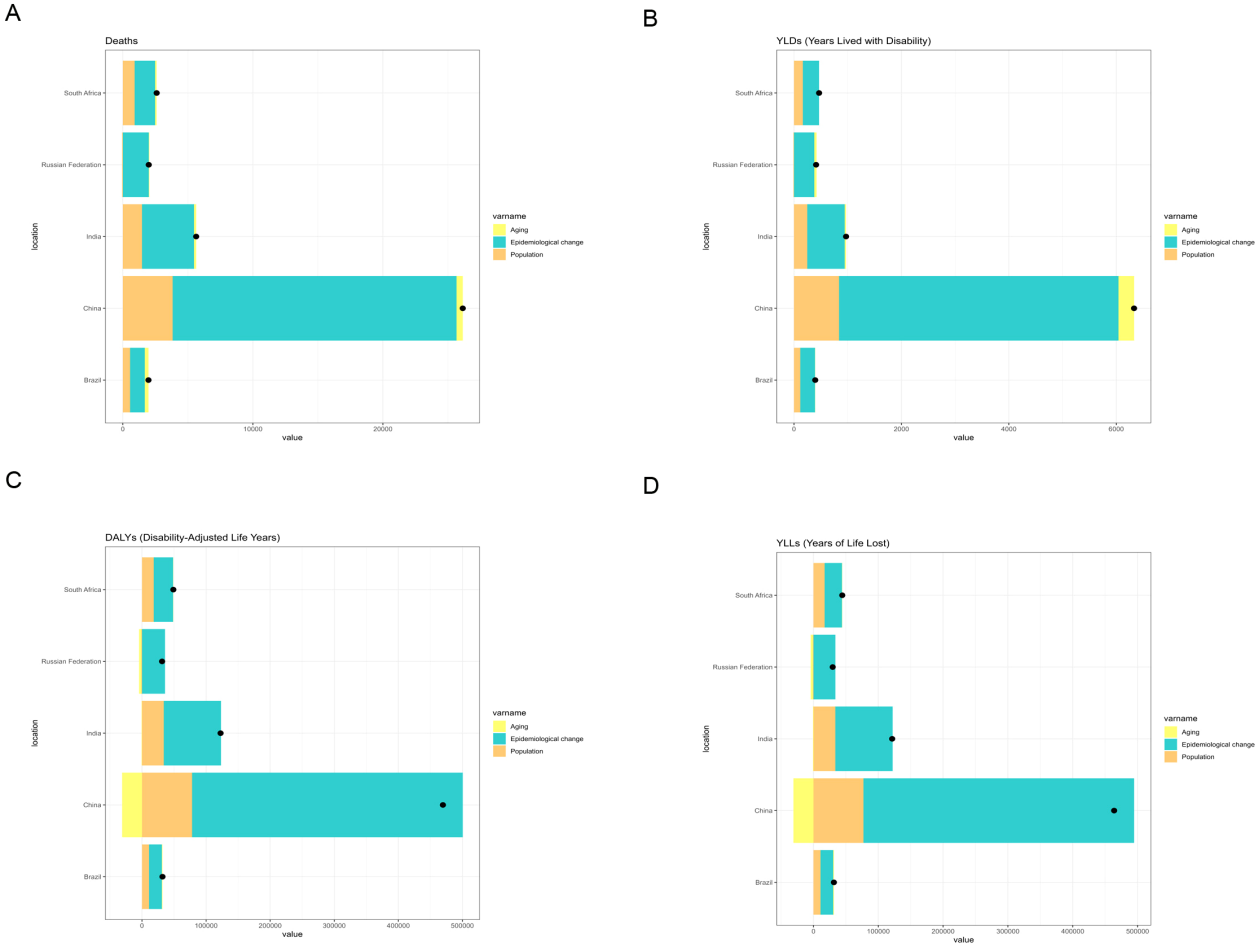
Changes in deaths, DALYs, YLDs, and YLLs from liver cancer attributable to high BMI according to population-level determinants (population growth, population ageing, and epidemiological change) in BRICS countries (1990–2021). **(A)** Changes in deaths. **(B)** Changes in YLDs. **(C)** Changes in DALYs. **(D)** Changes in YLLs.

**Figure 6:**
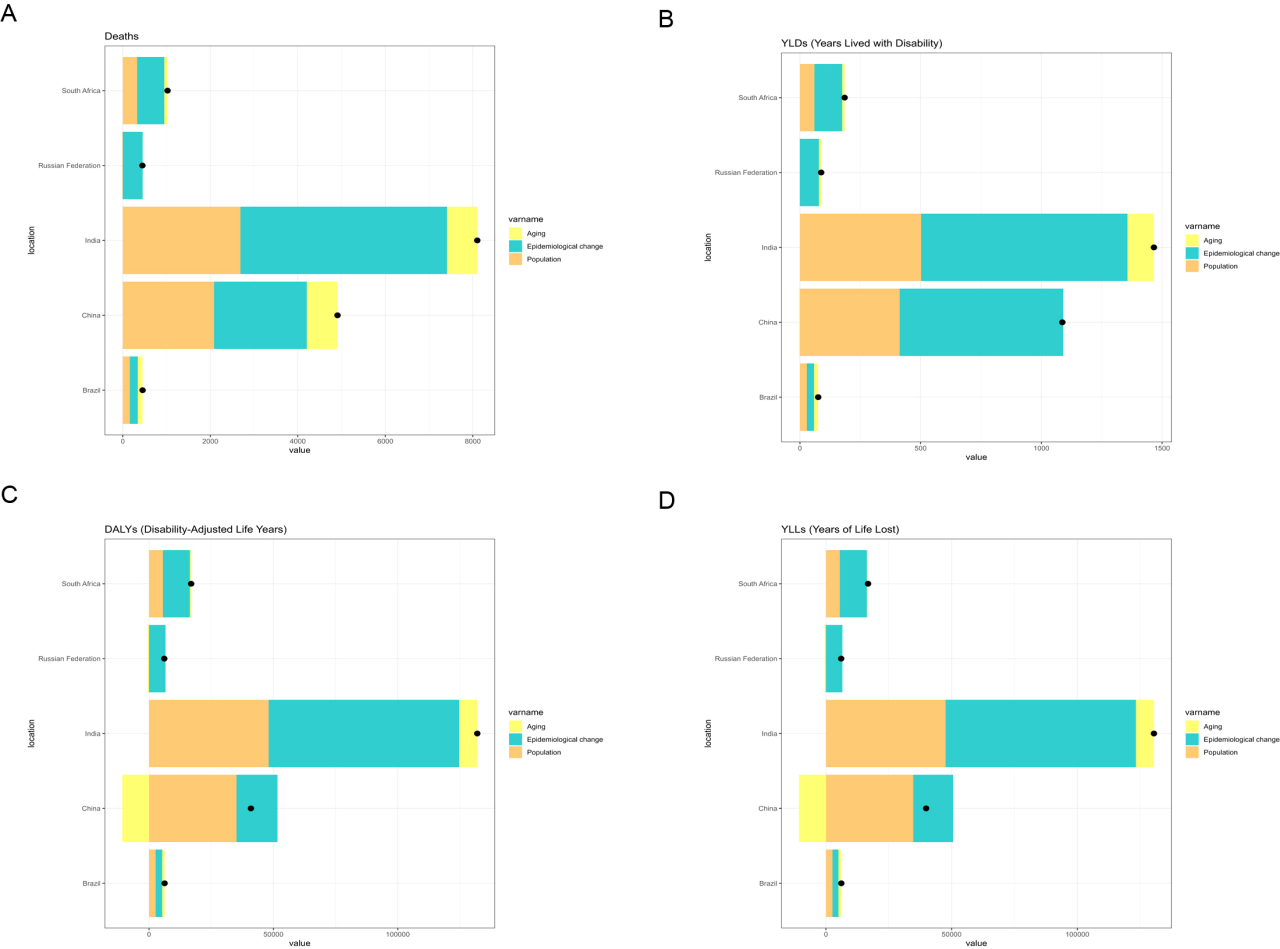
Changes in deaths, YLDs, DALYs, and YLLs from liver cancer associated with HFPG, according to population-level determinants (population growth, population ageing, and epidemiological change) in BRICS countries (1990–2021). **(A)** Changes in deaths. **(B)** Changes in YLDs. **(C)** Changes in DALYs. **(D)** Changes in YLLs.

For liver cancer attributable to high BMI, China exhibited the greatest increase in deaths and DALYs among BRICS countries between 1990 and 2021. Epidemiological change accounted for the largest share of this increase. In China, epidemiological change contributed 77.8% and 88.3% to the rise in deaths and DALYs, respectively, during this period (**Figure 5**). For liver cancer attributable to HFPG, India experienced the greatest increase in deaths and DALYs from 1990 to 2021, followed by China. In India, epidemiological change, population growth, and population ageing contributed 58.21%, 32.50% and 9.29%, respectively, to the increase in deaths. Correspondingly, epidemiological change, population growth, and population ageing contributed 58.04%, 35.72%, and 6.24%, respectively, to the increase in DALYs during the same period (**Figure 6**).

### 3.5 Projections to 2050

According to the ARIMA model, for liver cancer attributable to high BMI, deaths and DALYs in both females and males across BRICS countries are projected to increase from 2022 to 2050, with ASMR and ASDR expected to rise linearly in both sexes (**Supplementary Figures S25**-**S29**). For liver cancer attributable to HFPG, deaths and DALYs for both sexes in most BRICS countries are also projected to increase over this period, with ASMR and ASDR expected to increase linearly. However, the ASMR for both sexes is expected to remain stable, while ASDR is projected to remain stable in males but to fluctuate around a certain value in females (**Supplementary Figures S30**-**S34**).

According to the ES model, for liver cancer attributable to high BMI, deaths and DALYs in both females and males across BRICS countries are projected to increase from 2022 to 2050, with ASMR and ASDR expected to show gradual increases (**Supplementary Figures S35**-**S39**). For liver cancer attributable to HFPG, deaths and DALYs for both sexes in most BRICS countries are also projected to increase, with ASMR and ASDR expected to increase gradually. However, the ASMR for both sexes is expected to remain stable, while the ASDR for both sexes is projected to decline markedly compared with levels observed between 1990 and 2021 (**Supplementary Figures S40**-**S44**).

## 4 Discussion

Global health communities have expressed growing concern regarding the threat of liver cancer associated with metabolic risk factors such as high BMI and HFPG (22), particularly in rapidly developing countries. In BRICS countries (Brazil, Russia, India, China, and South Africa), the liver cancer burden attributable to these factors has shown a consistent upward trend, creating major public health challenges and necessitating urgent intervention. This study evaluates the liver cancer burden attributable to high BMI and HFPG in BRICS countries between 1990 and 2021, stratified by age and sex, thereby providing critical evidence to inform cancer prevention strategies in these emerging economies.

In 2021, South Africa bore the highest liver cancer burden among BRICS countries, particularly from high BMI-related cases, with an ASMR of 3.29 (95%UI: 1.4-5.6) and an ASDR of 84.81 (95%UI: 36.21–143.71) (**Figure 1**). Liver cancer attributable to HFPG also reached its highest values in South Africa, with an ASMR of 1.00 (95%UI: 0.09-2.12) and an ASDR of 20.07 (95%UI: 1.87-42.64) (**Figure 2**). These findings indicate that South Africa’s liver cancer burden is strongly shaped by high BMI and HFPG, a pattern likely linked to the country’s elevated prevalence of obesity and diabetes, together with relatively limited healthcare resources and insufficient screening coverage (23).

Between 1990 and 2021, India and Russia recorded the most rapid increases in liver cancer burden. In India, high BMI-related liver cancer exhibited the steepest rise in ASMR, with an EAPC of 5.64 (95%CI: 5.49-5.78), while the ASDR increased at an average annual rate of 5.45 (95%CI: 5.37-5.53) (**Figure 3**). This trend reflects the escalating prevalence of obesity, diabetes, and other metabolic disorders in India, driven by rapid economic development and urbanization, combined with insufficient early screening and intervention, leading to a sustained increase in metabolic-related liver cancer burden. Similarly, in Russia, a marked increase in liver cancer burden attributable to HFPG was observed, with an EAPC of 4.99 (95%CI: 4.55-5.42) for ASMR and 4.62 (95%CI: 4.20-5.05) for ASDR (**Figure 4**). This trend in Russia is likely linked to long-standing problems of obesity (24), entrenched patterns of alcohol consumption (25), and limited public health measures, which collectively intensified the impact of metabolic disorders on liver cancer burden.

The trends observed in South Africa, India, and Russia emphasize the regional heterogeneity within BRICS countries regarding the liver cancer burden attributable to high BMI and HFPG. South Africa bears the highest burden, likely linked to its complex socio-economic context and the fragility of its public health system (26, 27). The rapid increases documented in India and Russia reflect the acceleration of lifestyle transitions and the spread of metabolic disorders during economic development. These findings highlight the urgent need for targeted public health measures aimed at mitigating the liver cancer burden attributable to metabolic factors, particularly through strengthened obesity prevention, improved diabetes management, and expanded liver cancer screening coverage.

Additionally, pronounced sex disparities were identified in the liver cancer burden attributable to high BMI across BRICS countries. Both ASMR and ASDR were consistently higher in males than in females, with the disparity particularly marked in liver cancer linked to high BMI (**Supplementary Figure S1**). Comparable differences were also evident in liver cancer attributable to HFPG, with males generally presenting higher ASMR and ASDR (**Supplementary Figure S2**). These patterns may be explained by higher rates of obesity, greater prevalence of metabolic syndrome, and lifestyle-related factors in male populations.

From 1990 to 2021, the liver cancer burden attributable to high BMI in China increased steadily. The ASMR for females rose from 0.37 (95%UI: 0.15-0.66) to 1.19 (95%UI: 0.46-2.19), with an EAPC of 4.17 (95%CI: 4.02-4.33), while in males it increased from 0.73 (95%UI: 0.30-1.23) to 2.19 (95%UI: 0.83-4.15), with an EAPC of 3.81 (95%CI: 3.66-3.95). A comparable upward trend was noted for ASDR: in females it rose from 9.85 (95%UI: 4.01-17.38) to 29.67 (95%UI: 11.57–55.35), with an EAPC of 3.92 (95%CI: 3.79-4.05), while in males it increased from 22.32 (95%UI: 9.06–37.64) to 64.92 (95%UI: 24.41–124.21), with an EAPC of 3.65 (95%CI: 3.53–3.81), indicating a growing burden, particularly among males (**Supplementary Figure S3**). This trend illustrates the challenges China faces in addressing obesity-related liver cancer as obesity prevalence continues to rise (28). In contrast, the liver cancer burden attributable to HFPG declined slightly among females, with both ASMR and ASDR showing marginal decreases and EAPCs approaching zero or negative values. By comparison, in males the trend was upward: ASMR increased from 0.48 (95%UI: 0.05-1.03) to 0.55 (95%UI: 0.06-1.23), with an EAPC of 1.08 (95%CI: 0.76–1.40), and ASDR rose from 11.74 (95%UI: 1.11–25.41) to 12.74 (95%UI: 1.35-28.48), with an EAPC of 0.78 (95%CI: 0.52-1.07). This trend may reflect the cumulative impact of metabolic risks and lifestyle-related factors (**Supplementary Figure S4**).

For liver cancer attributable to high BMI, ASMR and ASDR generally increase with age in most BRICS countries, but decline after reaching advanced age. This pattern may be associated with age-related changes in physiology, immune function, and metabolism (29). Although the risk of liver cancer rises with age, survival in the older adult may be influenced by comorbid chronic diseases and other age-related conditions, resulting in stabilization or decline of mortality rates in the oldest age groups. In contrast, South Africa displays a divergent pattern, with both ASMR and ASDR continuing to rise with age. This atypical trend may reflect systemic challenges in health management, public health resources, and healthcare infrastructure. Compared with other BRICS countries, the liver cancer burden linked to high BMI and HFPG in South Africa appears closely associated with inadequate chronic disease management, limited early screening, and insufficient health education.

From 1990 to 2021, ASMR and ASDR for liver cancer attributable to high BMI increased annually across all age groups, reflecting the substantial global impact of obesity on liver cancer burden. In China, the most pronounced increases were observed in individuals aged over 95 years, with EAPCs of 6.38 and 6.34 for ASMR and ASDR, respectively. This sharp rise may reflect the cumulative effects of obesity and diabetes-related chronic conditions on liver cancer risk in this age group. Moreover, with the progression of population ageing, older cohorts may accumulate liver cancer risk over longer lifespans, resulting in a more rapid escalation of mortality in these groups.

A comparable trend was identified in the liver cancer burden attributable to HFPG. In the Chinese population aged ≥95 years, both ASMR and ASDR showed rapid increases (EAPCs of 3.66 and 3.63, respectively), emphasizing the considerable contribution of diabetes and metabolic syndrome to liver cancer progression. This pattern indicates that hepatocarcinogenesis in ageing populations is not solely associated with obesity but is intrinsically related to prolonged metabolic dysregulation and diabetic pathophysiology. With the acceleration of population ageing in BRICS nations, particularly in China and South Africa, where liver cancer burdens are rising, the implementation of effective health management strategies for older adult populations has become increasingly imperative. Within these cohorts, obesity and diabetes are emerging as pivotal determinants for liver cancer control in these regions.

Decomposition analysis demonstrated substantial changes in the liver cancer burden attributable to high BMI and HFPG across BRICS nations between 1990 and 2021. China and India, in particular, displayed divergent trends and underlying drivers in liver cancer-related deaths and DALYs associated with these metabolic risk factors.

For liver cancer attributable to high BMI, China exhibited the most pronounced increases in deaths and DALYs among BRICS countries. Epidemiological changes contributed 77.8% and 88.3% to the growth in deaths and DALYs, respectively, between 1990 and 2021. These findings indicate the escalating health risks associated with high BMI, particularly its contribution to liver carcinogenesis, within the context of China’s demographic shifts, including population ageing and rapid urban expansion. This trend may be linked to China’s accelerated economic development, urbanization, and dietary transitions, such as the widespread consumption of high-calorie and high-fat foods, which have markedly increased the prevalence of high BMI (30).

In contrast, India recorded the greatest increase in HFPG-attributable liver cancer among BRICS nations, followed by China. In India, epidemiological changes accounted for 58.21% of the rise in HFPG-related deaths and 58.04% of the increase in DALYs, whereas population growth and ageing contributed 32.5% and 35.72%, respectively. These patterns are consistent with India’s rapid urbanization, lifestyle transitions, and the rising prevalence of metabolic disorders such as diabetes. Nevertheless, systemic deficiencies in public health infrastructure and restricted access to healthcare (31, 32) have impeded the effective management of the dual burden imposed by hyperglycemia and liver cancer. With the acceleration of population ageing, India is likely to encounter escalating risks of a further increase in the liver cancer burden.

According to projections from the ARIMA and ES models, deaths and DALYs attributable to high BMI-related liver cancer in BRICS countries are anticipated to rise steadily between 2022 and 2050. Both ASMR and ASDR for women and men are predicted to follow a linear upward trend, suggesting that, despite heterogeneity in health policies and control strategies across BRICS nations, the global escalation of obesity will continue to drive an increasing liver cancer burden. For liver cancer attributable to HFPG, both models similarly forecast future increases in deaths and DALYs. In most BRICS countries, ASMR and ASDR in both sexes are expected to increase linearly. Nonetheless, the ASMR is projected to remain relatively stable, whereas the ASDR is predicted to exhibit fluctuations, with female ASDR potentially oscillating around a fixed range. These patterns may reflect variations in lifestyle behaviors, dietary practices, and the effectiveness of disease management among women.

## 5 Limitations

1. Data availability and completeness: The analysis was based on publicly accessible epidemiological data and forecasting models, which may be constrained by incomplete datasets or regional disparities.
2. Model assumptions and predictive uncertainty: Variations in liver cancer burden are influenced not only by high BMI and HFPG but also by broader socioeconomic, cultural, and environmental determinants that may not be fully incorporated within the ARIMA and ES models. Therefore, an element of uncertainty is inherent in the projections.
3. Exclusion of additional risk factors: The development of liver cancer is affected by a spectrum of determinants, including genetic predisposition, viral hepatitis, and alcohol consumption. The present study focused primarily on the contributions of high BMI and HFPG, without accounting for all potential risk factors, which may limit the comprehensiveness of the findings.
4. Temporal scope: The analysis encompassed data from 1990 to 2021. With ongoing lifestyle transitions and the introduction of novel public health interventions, the future trend of liver cancer burden may diverge from these estimates.

## 6 Conclusion

The liver cancer burden attributable to high BMI and HFPG in BRICS countries is projected to continue rising in the coming decades, particularly in relation to deaths and DALYs. Effective response requires the reinforcement of prevention, early screening, and treatment strategies, with particular emphasis on the management of obesity, diabetes, and metabolic syndrome. Furthermore, tailored public health policies should be formulated in accordance with sex-specific and national characteristics to more effectively address the escalating burden of liver cancer.

## Data Availability

The original contributions presented in the study are included in the article/**Supplementary Material**. Further inquiries can be directed to the corresponding authors.

## References

[1] Bray F Laversanne M Sung H Ferlay J Siegel RL Soerjomataram I . Global cancer statistics 2022: GLOBOCAN estimates of incidence and mortality worldwide for 36 cancers in 185 countries. CA Cancer J Clin. (2024) 74:229–63. doi: 10.3322/caac.21834, PMID: 38572751

[2] Luo C Liang J Sharabi K Hatting M Perry EA Tavares CDJ . Obesity/type 2 diabetes-associated liver tumors are sensitive to cyclin D1 deficiency. Cancer Res. (2020) 80:3215–21. doi: 10.1158/0008-5472.CAN-20-0106, PMID: 32606000 PMC7442681

[3] Trombetta M Spiazzi G Zoppini G Muggeo M . Review article: type 2 diabetes and chronic liver disease in the Verona diabetes study. Aliment Pharmacol Ther. (2005) 22 Suppl 2:24–7. doi: 10.1111/j.1365-2036.2005.02590.x, PMID: 16225467

[4] Xie J Zhang X Shao H Jing S Shan T Shi Y . An affordable approach to classifying type 2 diabetes based on fasting plasma glucose, TyG index and BMI: a retrospective cohort study of NHANES Data from 1988 to 2014. Diabetol Metab Syndr. (2022) 14:113. doi: 10.1186/s13098-022-00883-0, PMID: 35948978 PMC9364489

[5] Shukla A Kumar K Singh A . Association between obesity and selected morbidities: a study of BRICS countries. PloS One. (2014) 9:e94433. doi: 10.1371/journal.pone.0094433, PMID: 24718033 PMC3981786

[6] Alpízar Salazar M Olguín Reyes SE Medina Estévez A Saturno Lobos JA De Aldecoa Castillo JM Carrera Aguas JC . Natural history of metabolic dysfunction-associated steatotic liver disease: from metabolic syndrome to hepatocellular carcinoma. Med (Kaunas). (2025) 61. doi: 10.3390/medicina61010088, PMID: 39859069 PMC11766802

[7] Baffy G . Hepatocellular carcinoma in non-alcoholic fatty liver disease: epidemiology, pathogenesis, and prevention. J Clin Transl Hepatol. (2013) 1:131–7. doi: 10.14218/JCTH.2013.00005, PMID: 26355775 PMC4521282

[8] Marengo A Rosso C Bugianesi E . Liver cancer: connections with obesity, fatty liver, and cirrhosis. Annu Rev Med. (2016) 67:103–17. doi: 10.1146/annurev-med-090514-013832, PMID: 26473416

[9] Sohn W Lee HW Lee S Lim JH Lee MW Park CH . Obesity and the risk of primary liver cancer: A systematic review and meta-analysis. Clin Mol Hepatol. (2021) 27:157–74. doi: 10.3350/cmh.2020.0176, PMID: 33238333 PMC7820201

[10] Stella L Santopaolo F Gasbarrini A Pompili M Ponziani FR . Viral hepatitis and hepatocellular carcinoma: From molecular pathways to the role of clinical surveillance and antiviral treatment. World J Gastroenterol. (2022) 28:2251–81. doi: 10.3748/wjg.v28.i21.2251, PMID: 35800182 PMC9185215

[11] Alqahtani SA Colombo M . Treatment for viral hepatitis as secondary prevention for hepatocellular carcinoma. Cells. (2021) 10. doi: 10.3390/cells10113091, PMID: 34831314 PMC8619578

[12] Blumberg BS . Hepatitis B virus, the vaccine, and the control of primary cancer of the liver. Proc Natl Acad Sci U S A. (1997) 94:7121–5. doi: 10.1073/pnas.94.14.7121, PMID: 9207053 PMC33676

[13] Broeckhoven E Dallmeier K . Mission 2030: Toward universal hepatitis B immunization. Hum Vaccin Immunother. (2025) 21:2473222. doi: 10.1080/21645515.2025.2473222, PMID: 40023933 PMC11875460

[14] GBD 2021 Risk Factors Collaborators . Global burden and strength of evidence for 88 risk factors in 204 countries and 811 subnational locations, 1990-2021: a systematic analysis for the Global Burden of Disease Study 2021. Lancet. (2024) 403:2162–203. doi: 10.1016/S0140-6736(24)00933-4, PMID: 38762324 PMC11120204

[15] GBD 2021 Diseases and Injuries Collaborators . Global incidence, prevalence, years lived with disability (YLDs), disability-adjusted life-years (DALYs), and healthy life expectancy (HALE) for 371 diseases and injuries in 204 countries and territories and 811 subnational locations, 1990-2021: a systematic analysis for the Global Burden of Disease Study 2021. Lancet. (2024) 403:2133–61. doi: 10.1016/S0140-6736(24)00757-8, PMID: 38642570 PMC11122111

[16] Evaluation ifHMa. In: GBD results tool. Seattle, WA: Institute for Health Metrics and Evaluation (IHME). Available online at: https://ghdx.healthdata.org/gbd-results-tool (Accessed February 27, 2025).

[17] Zhang M Yuan L Cui M Chen J Jia J Zhao M . Analysis the burden of breast cancer among adolescents and young adults using the global burden of disease 2021. Ann Surg Oncol. (2025) 32:2056–69. doi: 10.1245/s10434-024-16648-0, PMID: 39668310 PMC11811250

[18] GBD 2021 Causes of Death Collaborators . Global burden of 288 causes of death and life expectancy decomposition in 204 countries and territories and 811 subnational locations, 1990-2021: a systematic analysis for the Global Burden of Disease Study 2021. Lancet. (2024) 403:2100–32. doi: 10.1016/S0140-6736(24)00367-2, PMID: 38582094 PMC11126520

[19] Zi H He SH Leng XY Xu XF Huang Q Weng H . Global, regional, and national burden of kidney, bladder, and prostate cancers and their attributab le risk factors, 1990-2019. Mil Med Res. (2021) 8:60. doi: 10.1186/s40779-021-00354-z, PMID: 34819142 PMC8611255

[20] Xie Y Bowe B Mokdad AH Xian H Yan Y Li T . Analysis of the Global Burden of Disease study highlights the global, regional, and national trends of chronic kidney disease epidemiology from 1990 to 2016. Kidney Int. (2018) 94:567–81. doi: 10.1016/j.kint.2018.04.011, PMID: 30078514

[21] Huang P Zhang J . Global leukemia burden and trends: a comprehensive analysis of temporal and spatial variations from 1990–2021 using GBD (Global Burden of Disease) data. BMC Public Health. (2025) 25:262. doi: 10.1186/s12889-025-21428-w, PMID: 39838416 PMC11753064

[22] Mao D Lau ESH Wu H Yang A Fan B Shi M . Risk associations of glycemic burden and obesity with liver cancer-A 10-year analysis of 15,280 patients with type 2 diabetes. Hepatol Commun. (2022) 6:1350–60. doi: 10.1002/hep4.1891, PMID: 35044101 PMC9134801

[23] van der Merwe MT Pepper MS . Obesity in South Africa. Obes Rev. (2006) 7:315–22. doi: 10.1111/j.1467-789X.2006.00237.x, PMID: 17038125

[24] Martinchik AN Baturin AK Keshabyants EE Peskova EV . Gender and age characteristics and the trends in prevalence of obesity in the adult population in Russia during the 1994–2012 period. Vopr Pitan. (2015) 84:50–7., PMID: 26863806

[25] Jargin S . Alcohol consumption in Russia and some aspects of public health. Int J High Risk Behav Addict. (2016) 5:e26617. doi: 10.5812/ijhrba.26617, PMID: 27162763 PMC4859937

[26] Younger DS . Health care in South Africa. Neurol Clin. (2016) 34:1127–36. doi: 10.1016/j.ncl.2016.06.004, PMID: 27719994

[27] Malakoane B Heunis JC Chikobvu P Kigozi NG Kruger WH . Public health system challenges in the Free State, South Africa: a situation appraisal to inform health system strengthening. BMC Health Serv Res. (2020) 20:58. doi: 10.1186/s12913-019-4862-y, PMID: 31973740 PMC6979387

[28] In China T Hu SS . Report on cardiovascular health and diseases in China 2021: an updated summary. J Geriatr Cardiol. (2023) 20:399–430. doi: 10.26599/1671-5411.2023.06.001, PMID: 37416519 PMC10320777

[29] Moulias S . Nutrition and immunity in the older adult. Ann Med Interne (Paris). (2002) 153:446–9. 12598830

[30] Pan XF Wang L Pan A . Epidemiology and determinants of obesity in China. Lancet Diabetes Endocrinol. (2021) 9:373–92. doi: 10.1016/S2213-8587(21)00045-0, PMID: 34022156

[31] Mistry N Venkateswaran S Baru R Patel V . Editorial: Realizing universal health coverage in India. Front Public Health. (2023) 11:1243676. doi: 10.3389/fpubh.2023.1243676, PMID: 37575104 PMC10421653

[32] Sarin SK Prasad M Ramalingam A Kapil U . Integration of public health measures for NAFLD into India’s national programme for NCDs. Lancet Gastroenterol Hepatol. (2021) 6:777–8. doi: 10.1016/S2468-1253(21)00264-8, PMID: 34509189

