# Supplementary material for: Liver cancer burden attributable to high body mass index and high fasting plasma glucose in BRICS countries (1990–2021): a Global Burden of Disease 2021 analysis with projections to 2050
Source: Front Oncol. 2025 Nov 7;15:1627282. doi: 10.3389/fonc.2025.1627282 (PMC12634373; doi:10.3389/fonc.2025.1627282)
Supplement: Supplementary file 1 [file DataSheet1.docx]

Supplementary Material

# Supplementary Figures

**
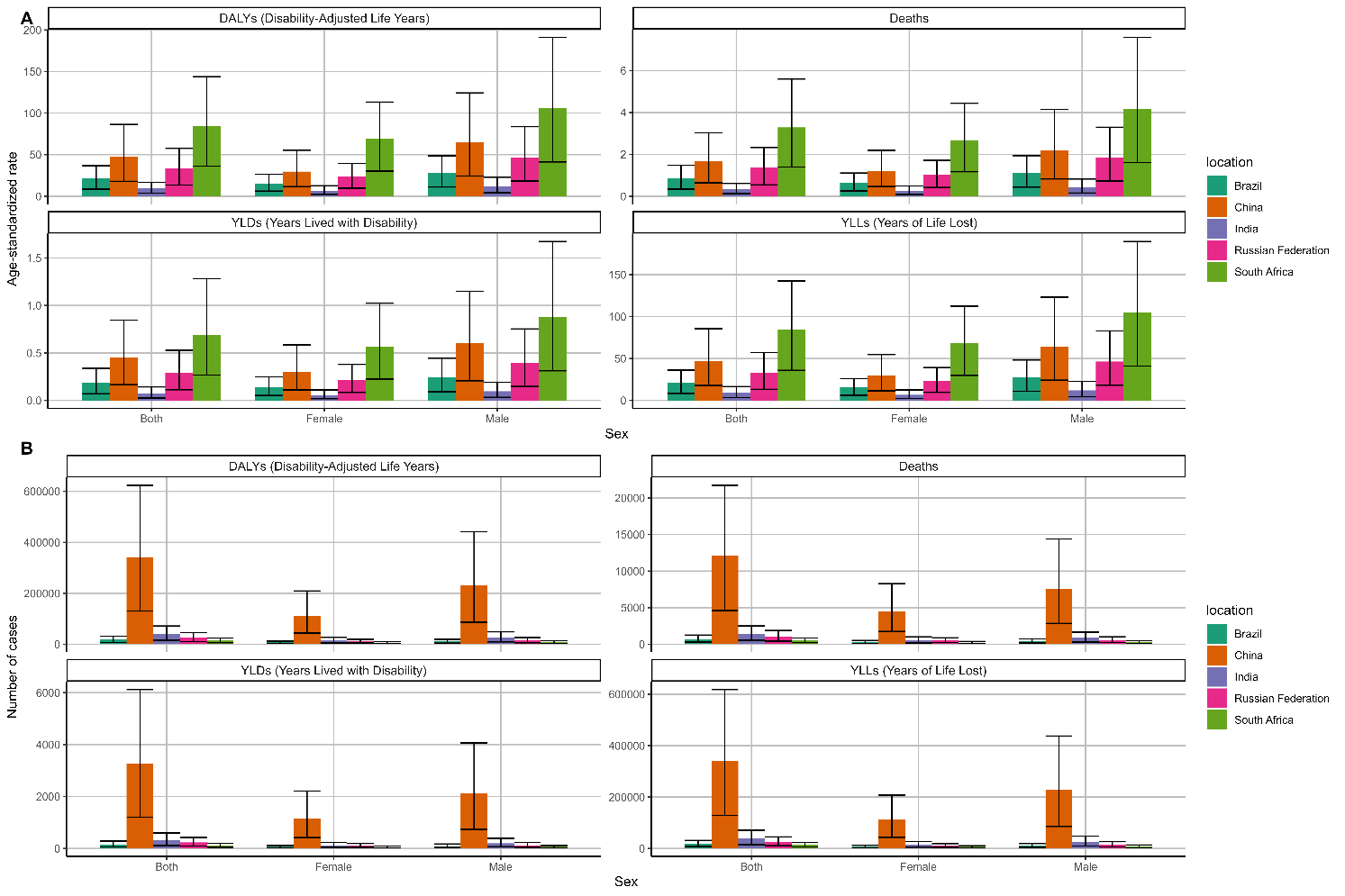
Supplementary Figure 1.** The disease burden of liver cancer attributable to high body mass index (HBMI) in BRICS countries by sex in 2021. (A) age-standardized rate, (B) number

**
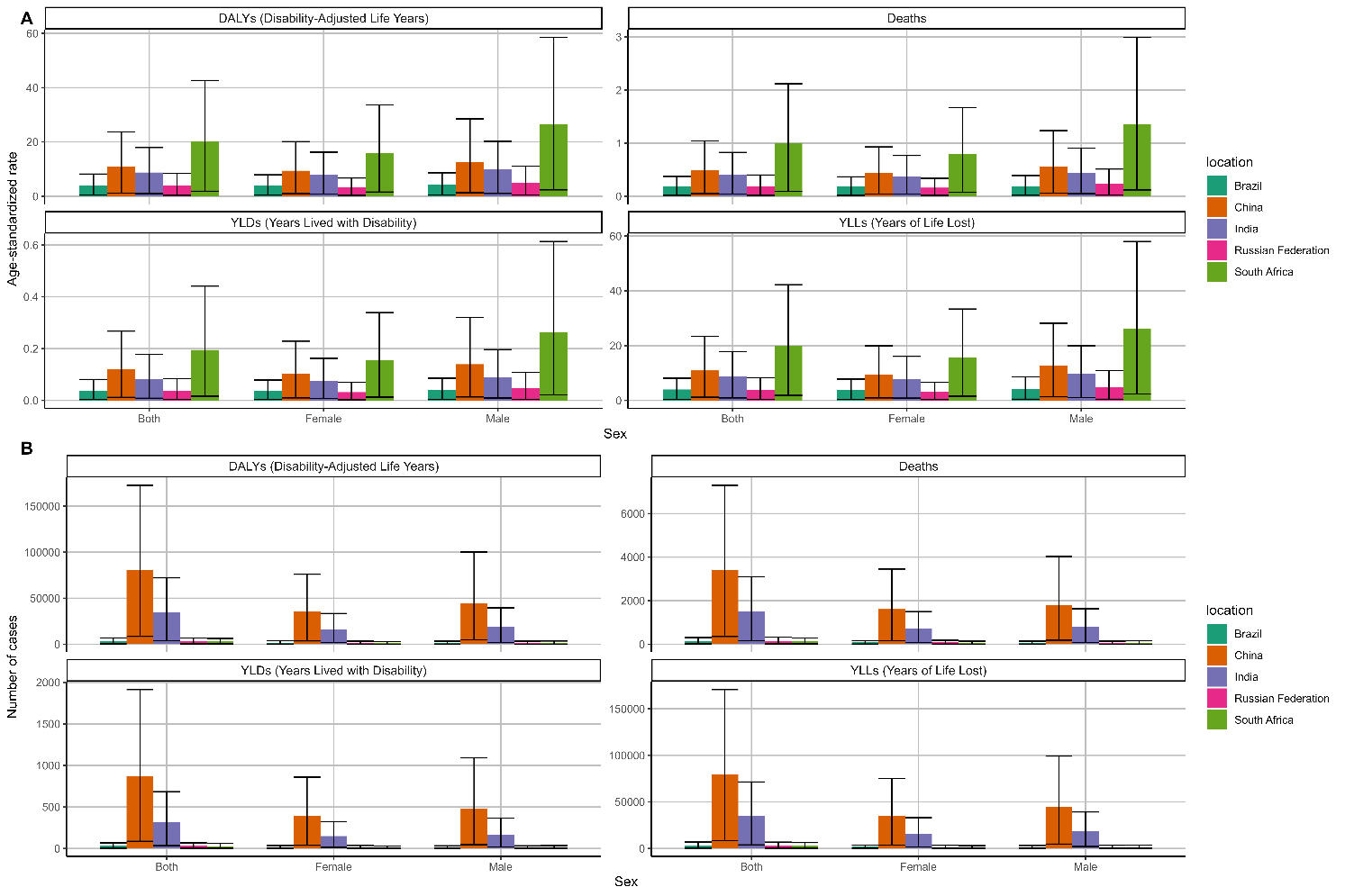
Supplementary Figure 2.** The disease burden of liver cancer attributable to high fasting plasma glucose (HFPG) in BRICS countries by sex in 2021. (A) age-standardized rate, (B) number

**
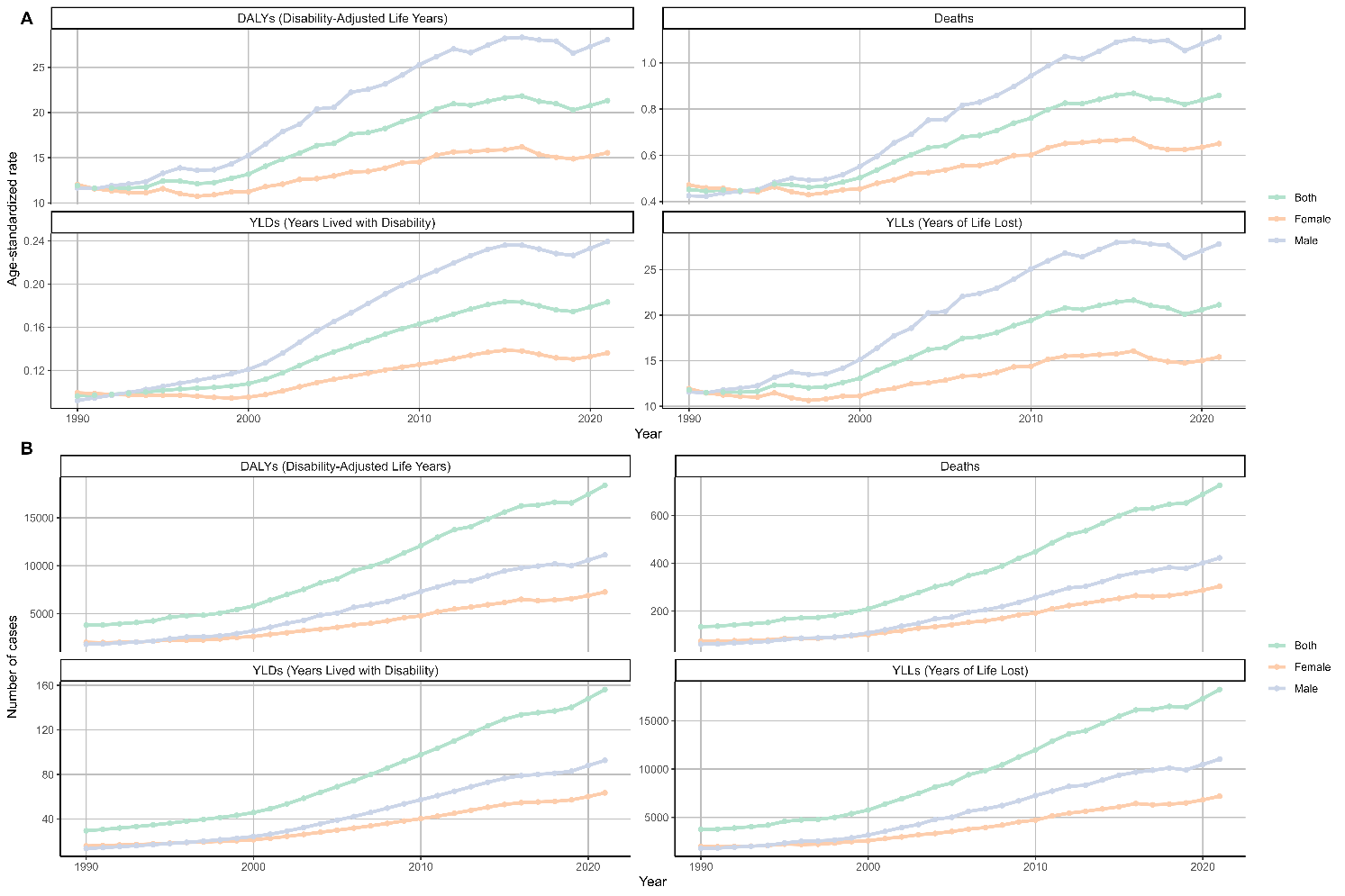
Supplementary Figure 3.** Trends in the disease burden of liver cancer attributable to high body mass index (HBMI) in Brazil by sex from 1990 to 2021.

**
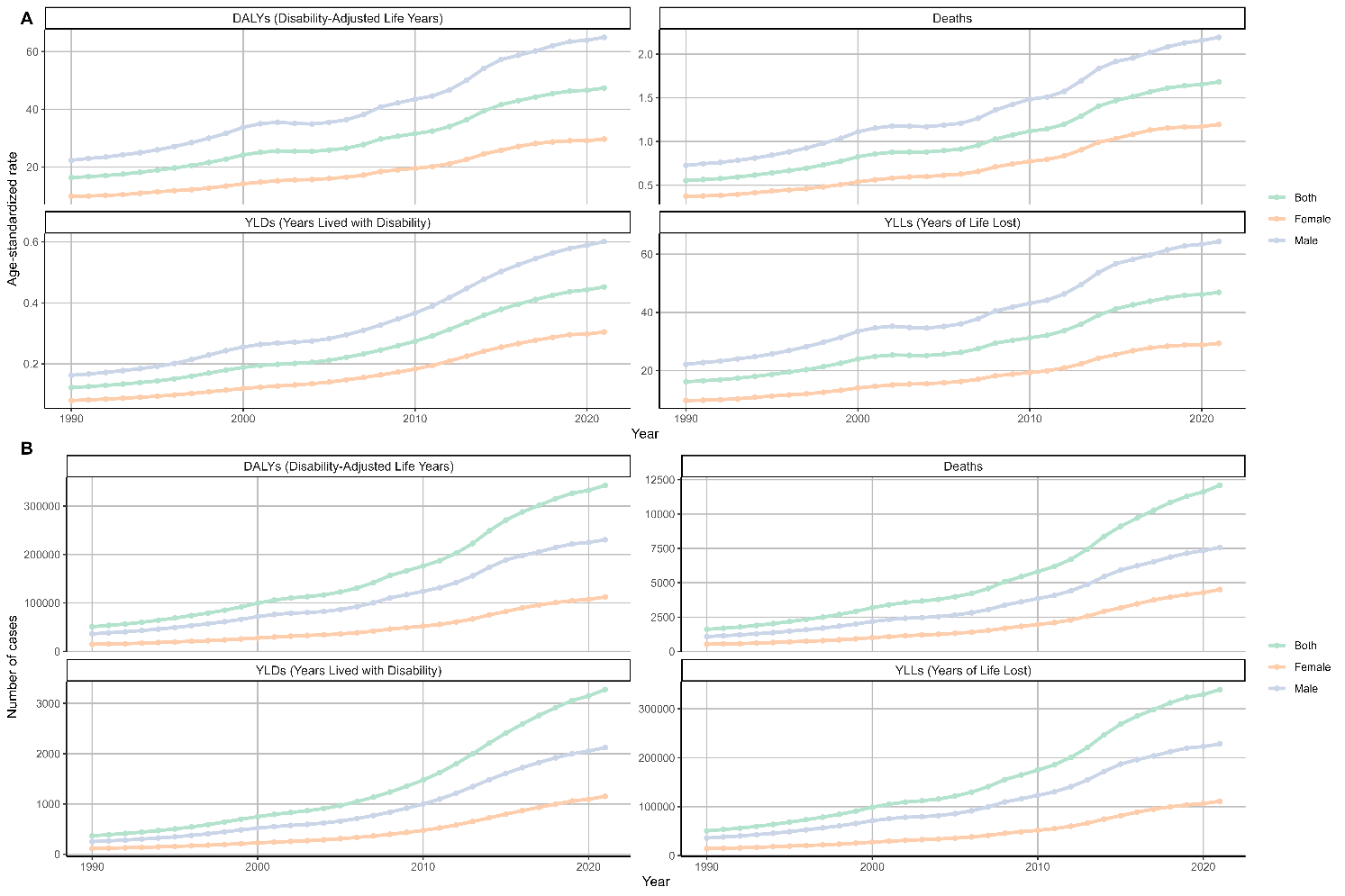
Supplementary Figure 4.** Trends in the disease burden of liver cancer attributable to high body mass index (HBMI) in China by sex from 1990 to 2021.

**
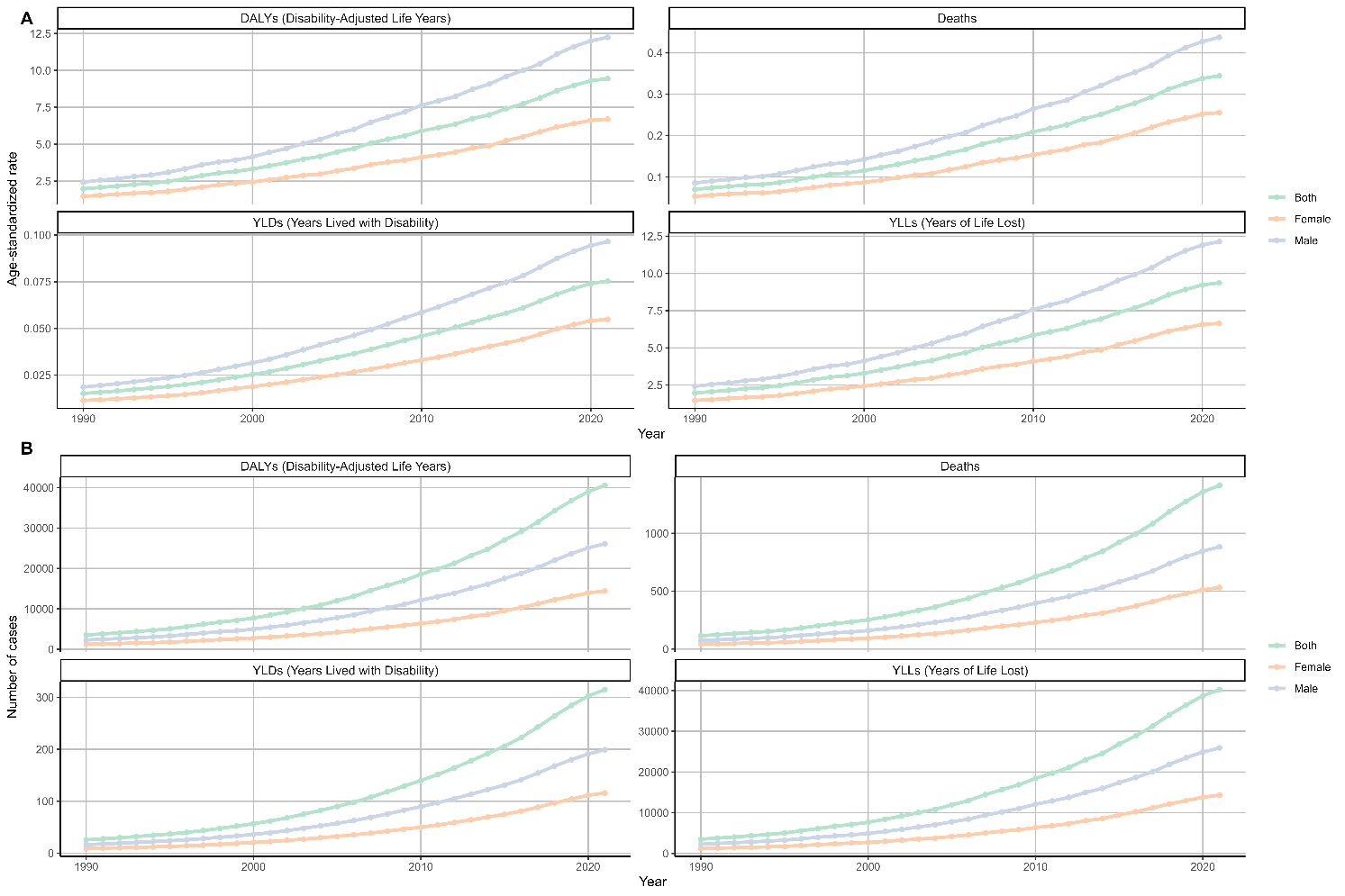
Supplementary Figure 5.** Trends in the disease burden of liver cancer attributable to high body mass index (HBMI) in India by sex from 1990 to 2021.

**
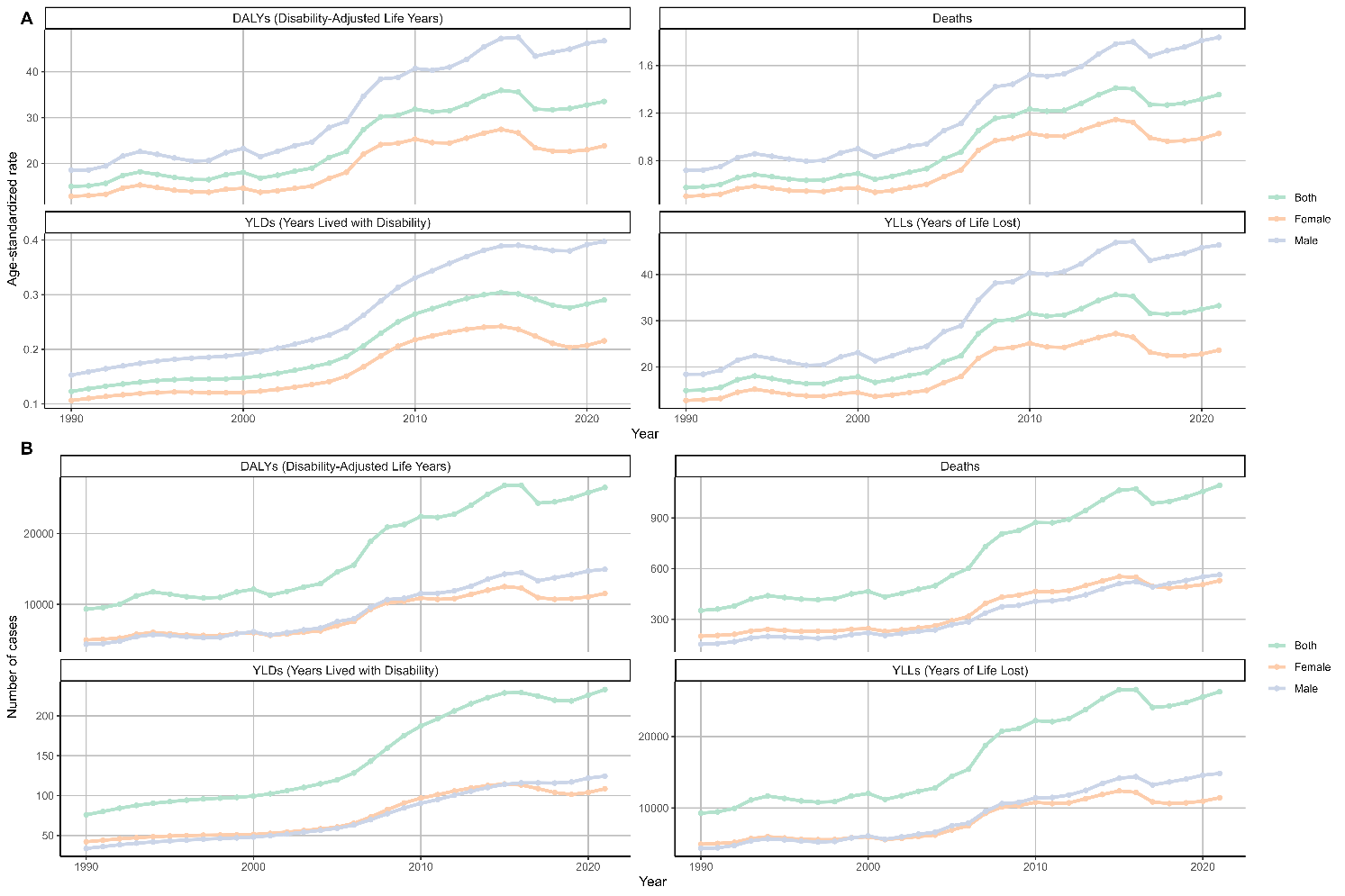
Supplementary Figure 6.** Trends in the disease burden of liver cancer attributable to high body mass index (HBMI) in Russian by sex from 1990 to 2021.

**
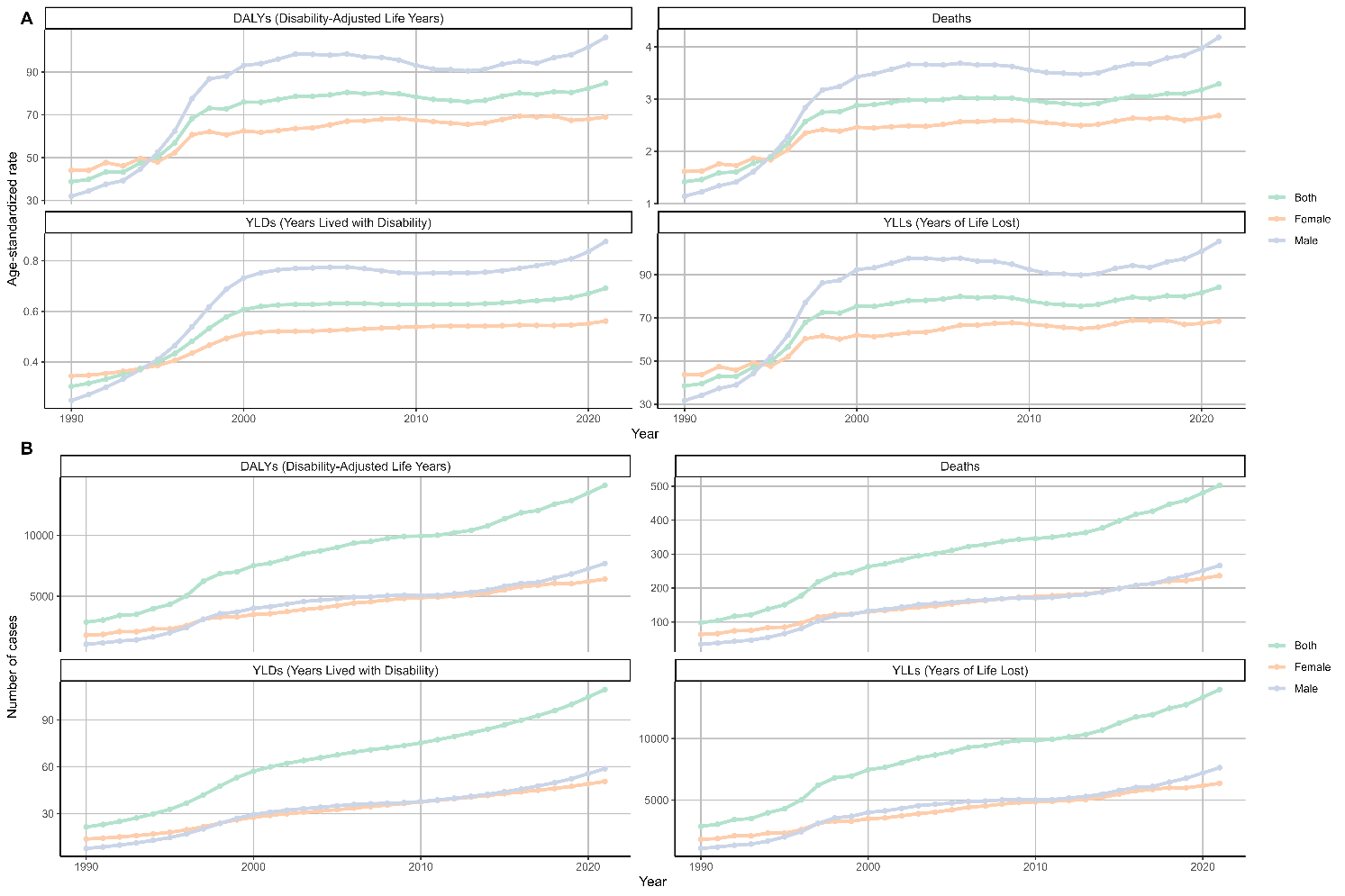
Supplementary Figure 7.** Trends in the disease burden of liver cancer attributable to high body mass index (HBMI) in South Africa by sex from 1990 to 2021.

**
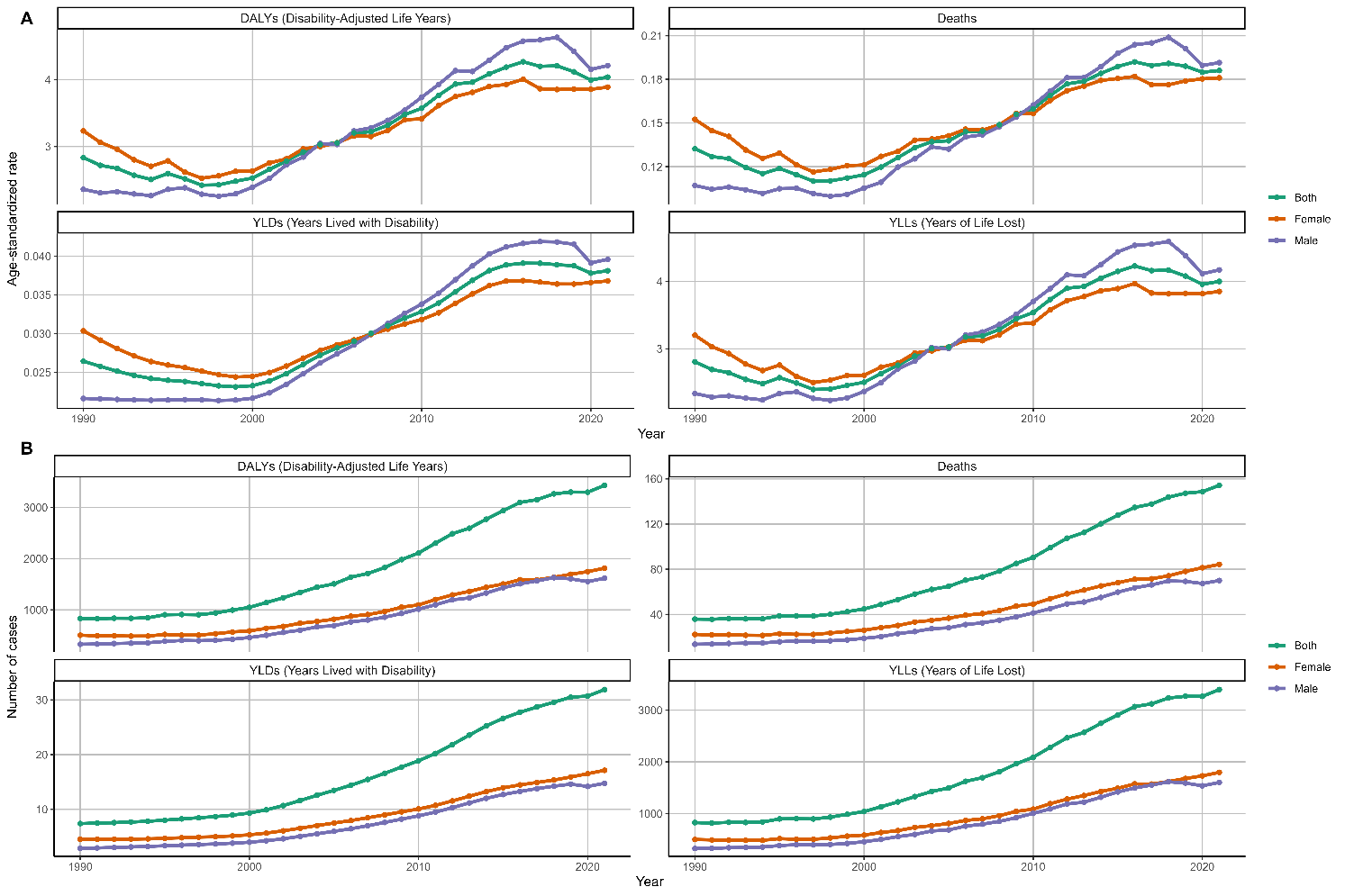
Supplementary Figure 8.** Trends in the disease burden of liver cancer attributable to high fasting plasma glucose (HFPG) in Brazil by sex from 1990 to 2021.

**
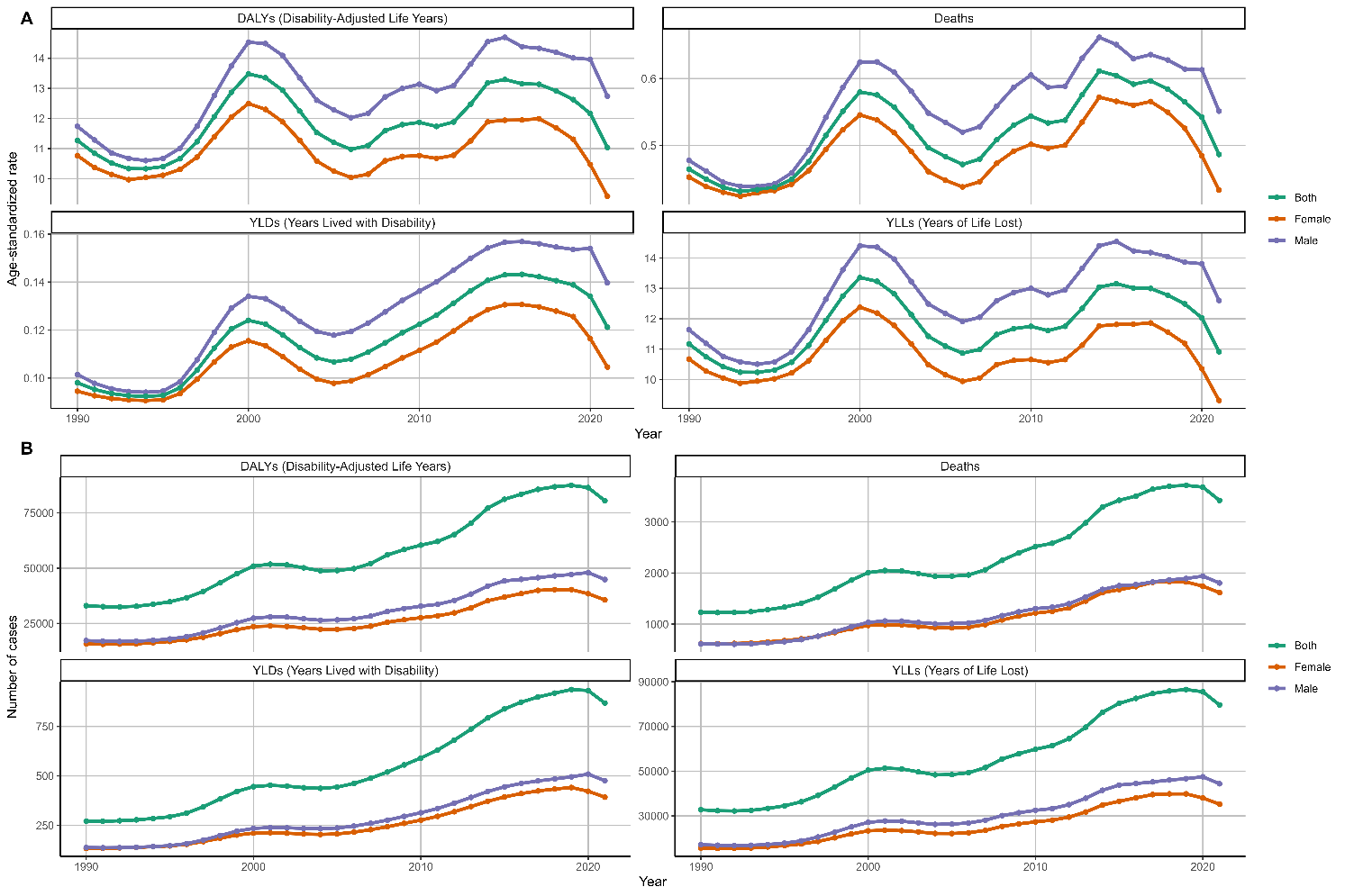
Supplementary Figure 9.** Trends in the disease burden of liver cancer attributable to high fasting plasma glucose (HFPG) in China by sex from 1990 to 2021.

**
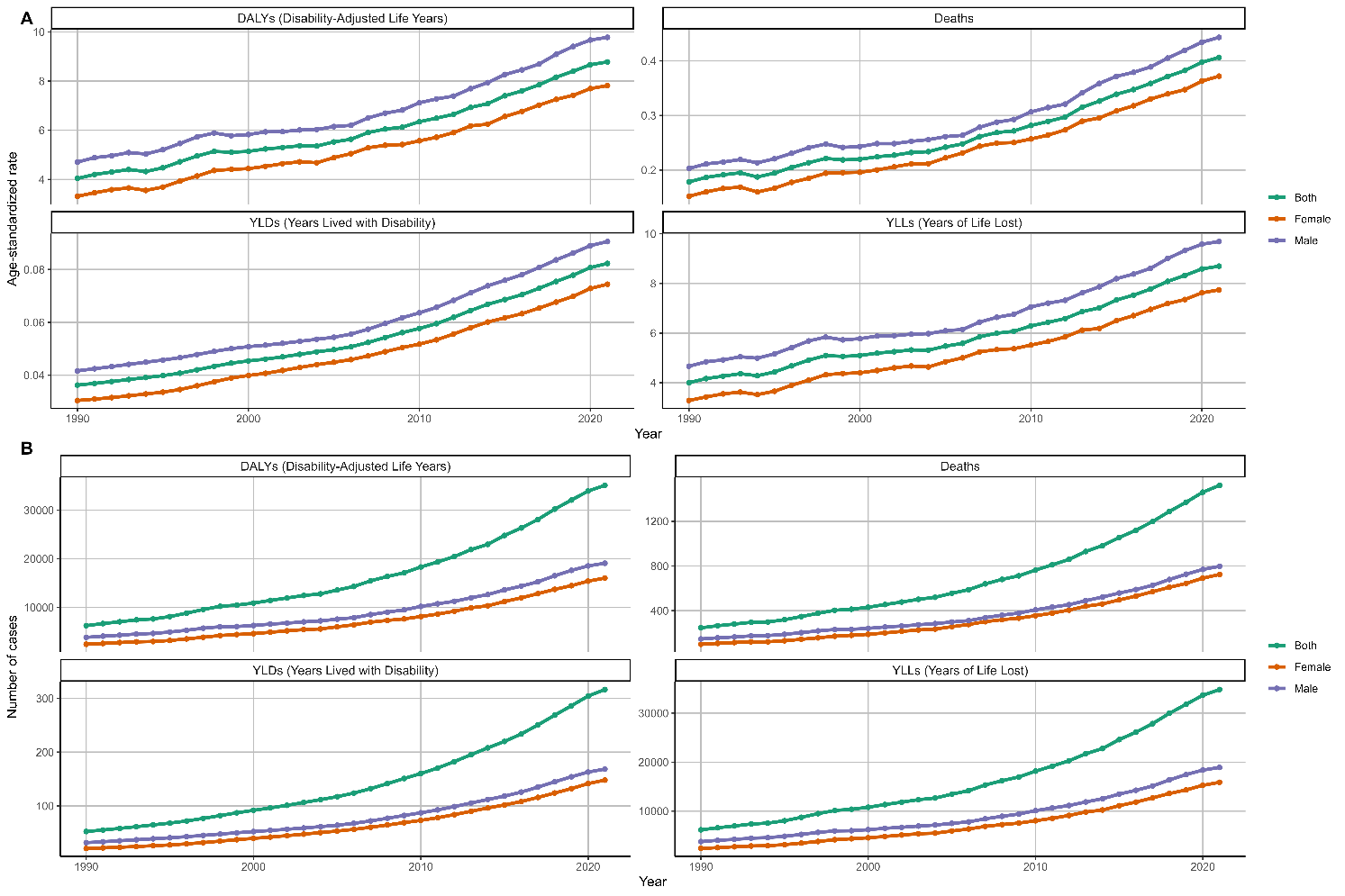
Supplementary Figure 10.** Trends in the disease burden of liver cancer attributable to high fasting plasma glucose (HFPG) in India by sex from 1990 to 2021.

**
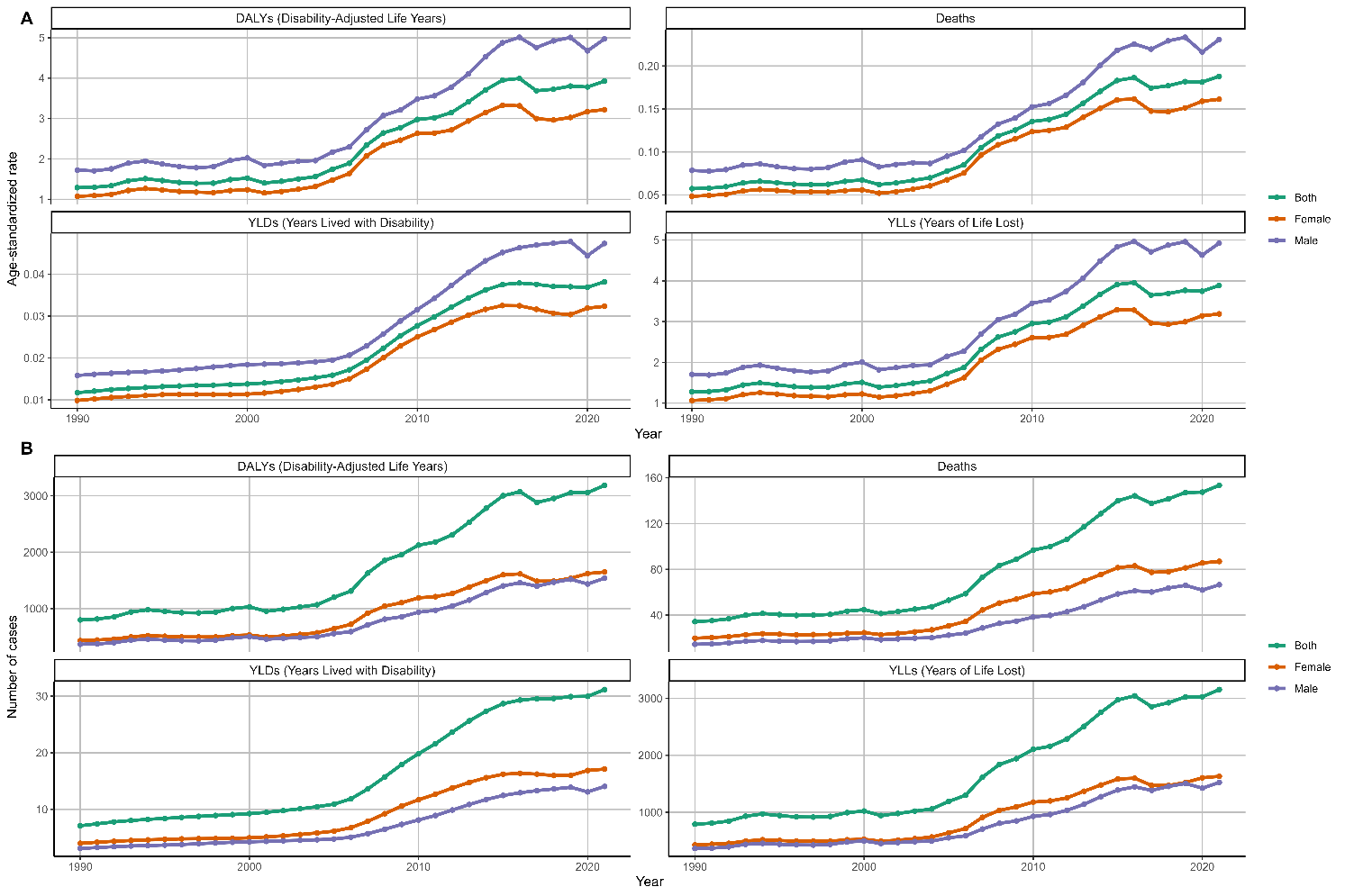
Supplementary Figure 11.** Trends in the disease burden of liver cancer attributable to high fasting plasma glucose (HFPG) in Russian by sex from 1990 to 2021.

**
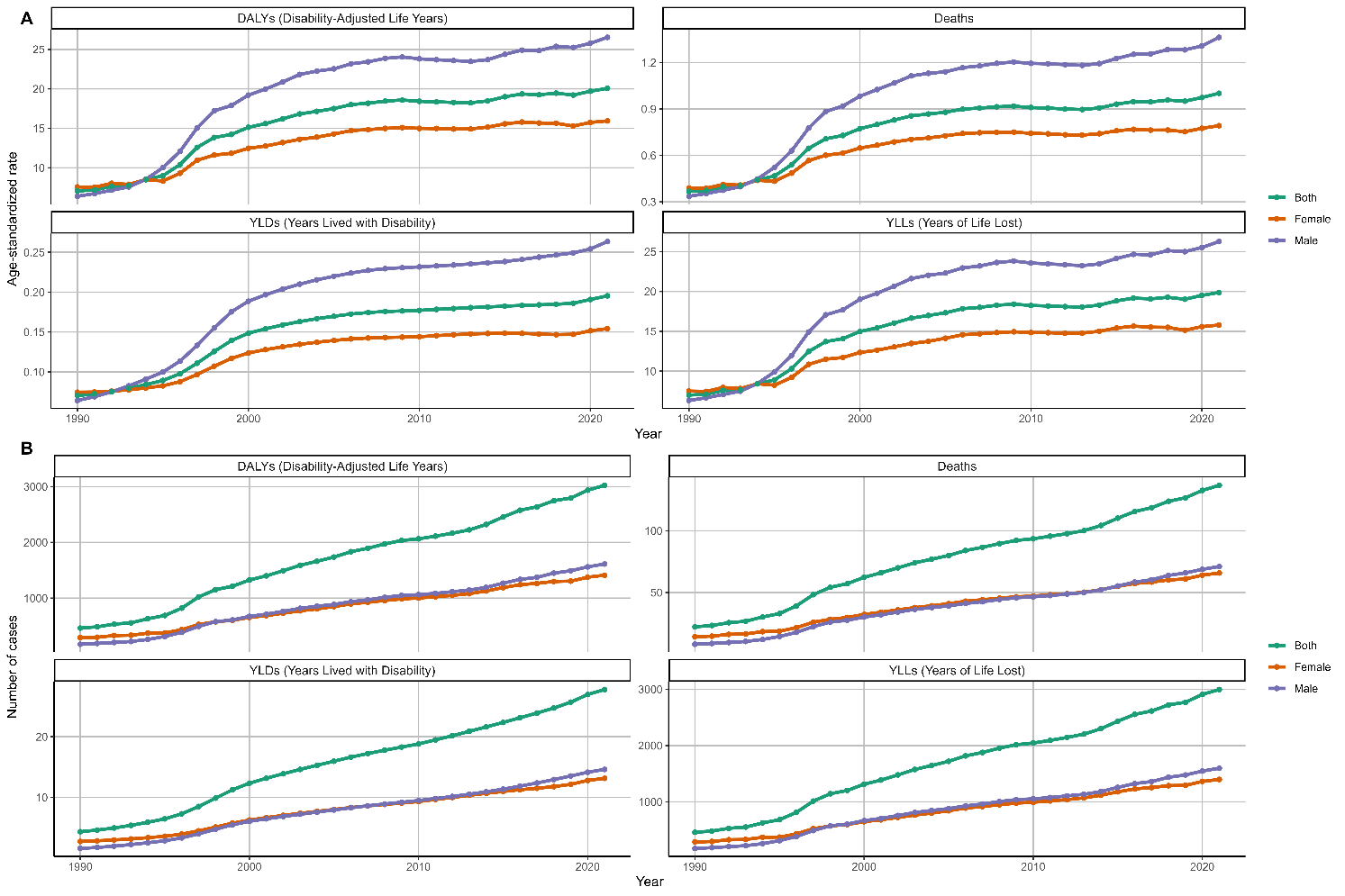
Supplementary Figure 12.** Trends in the disease burden of liver cancer attributable to high fasting plasma glucose (HFPG) in South Africa by sex from 1990 to 2021.

**
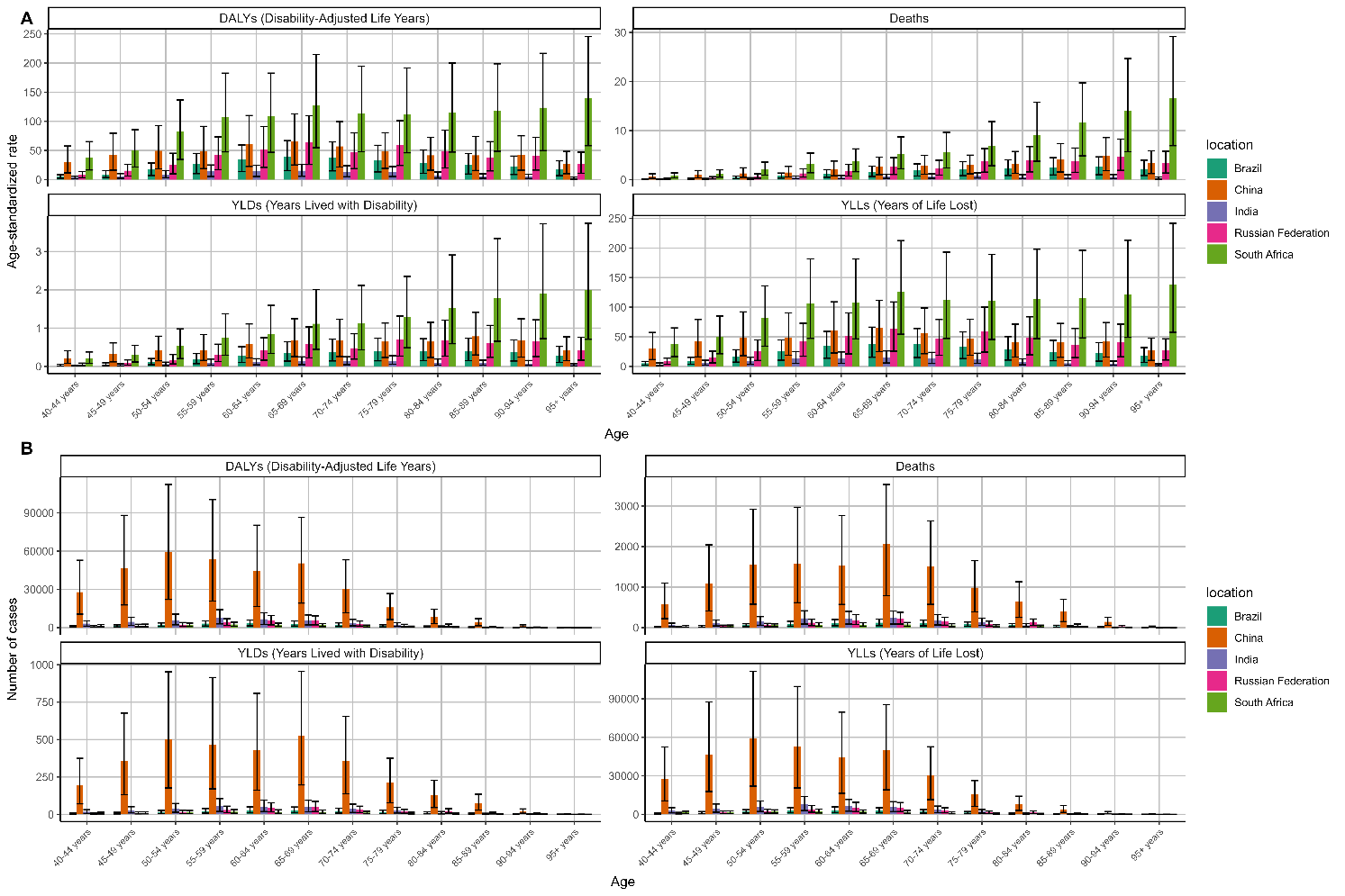
Supplementary Figure 13.** The disease burden of liver cancer attributable to high body mass index (HBMI) in BRICS countries by age in 2021. (A) age-standardized rate, (B) number

**
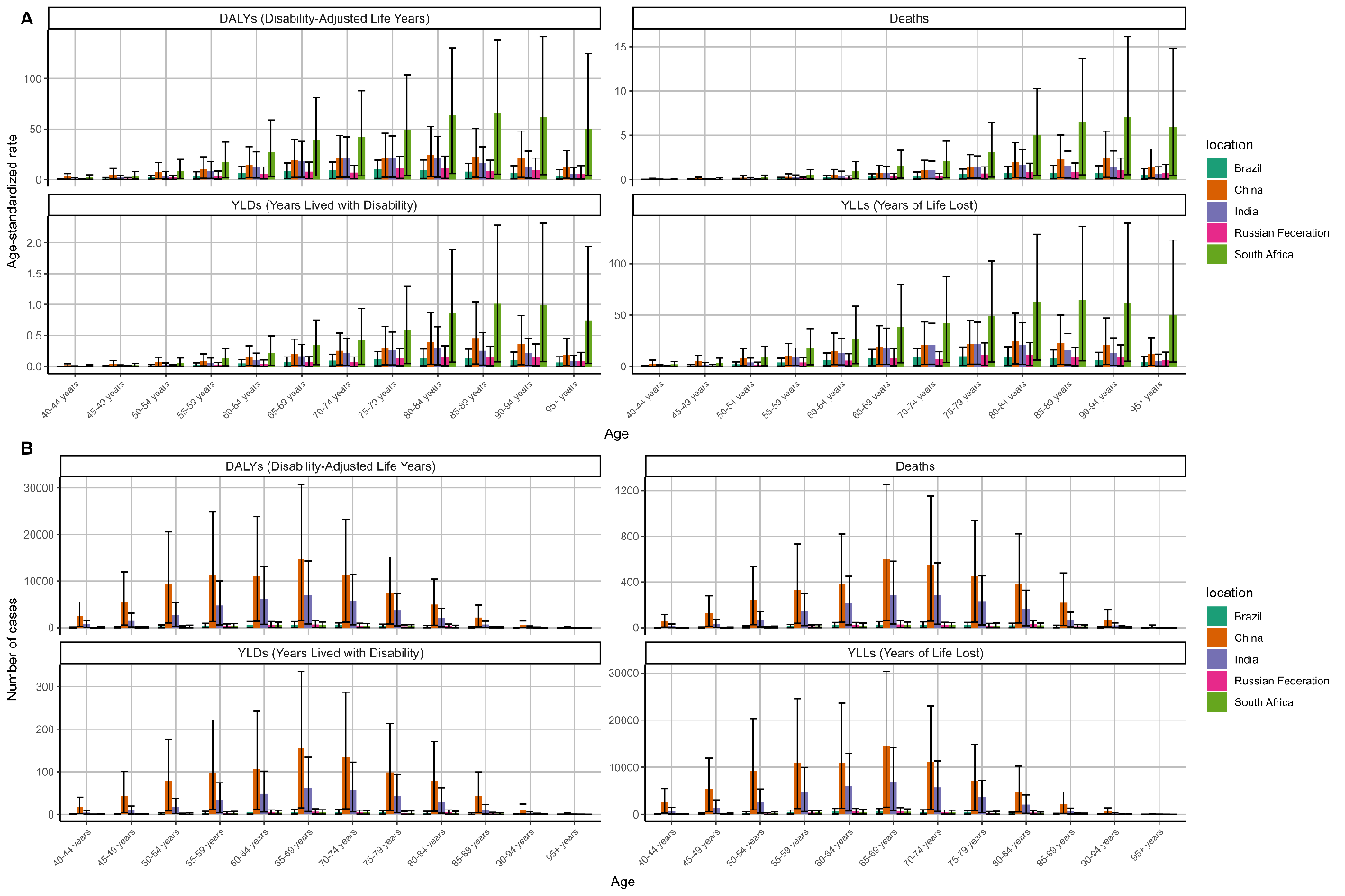
Supplementary Figure 14.** The disease burden of liver cancer attributable to high fasting plasma glucose (HFPG) in BRICS countries by age in 2021. (A) age-standardized rate, (B) number

**
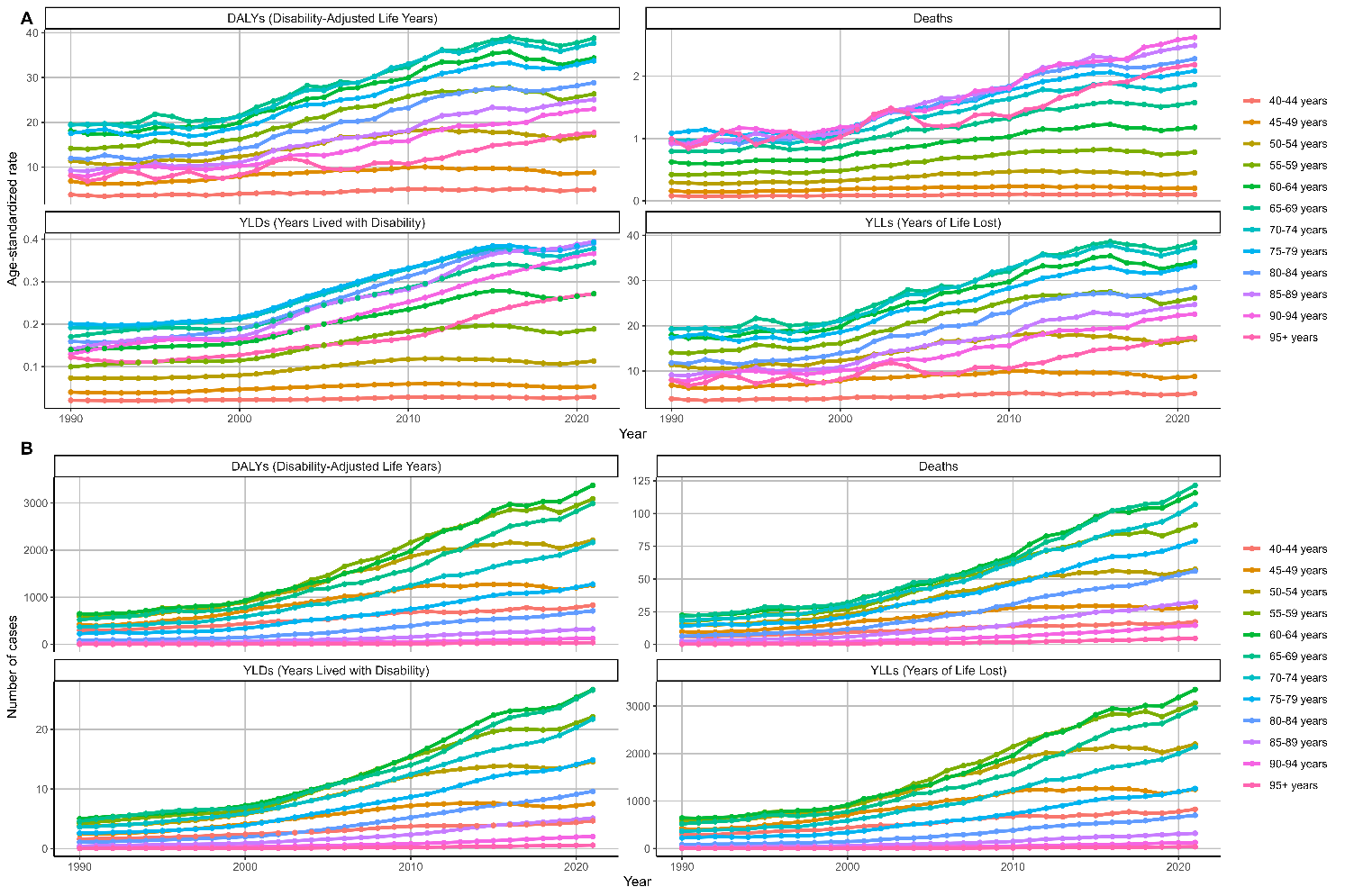
Supplementary Figure 15.** Trends in the disease burden of liver cancer attributable to high body mass index (HBMI) in Brazil by age from 1990 to 2021.

**
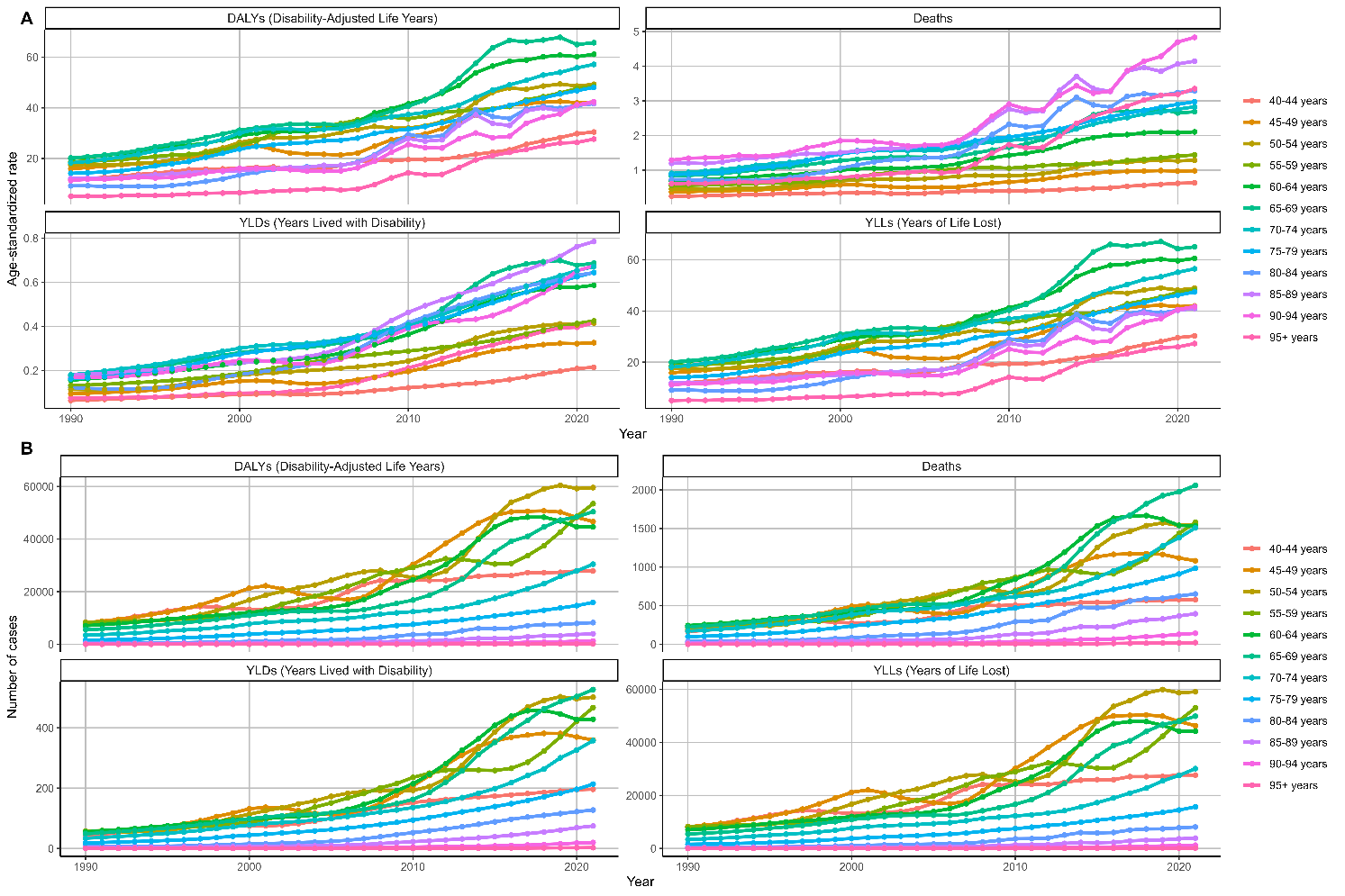
Supplementary Figure 16.** Trends in the disease burden of liver cancer attributable to high body mass index (HBMI) in China by age from 1990 to 2021.

**
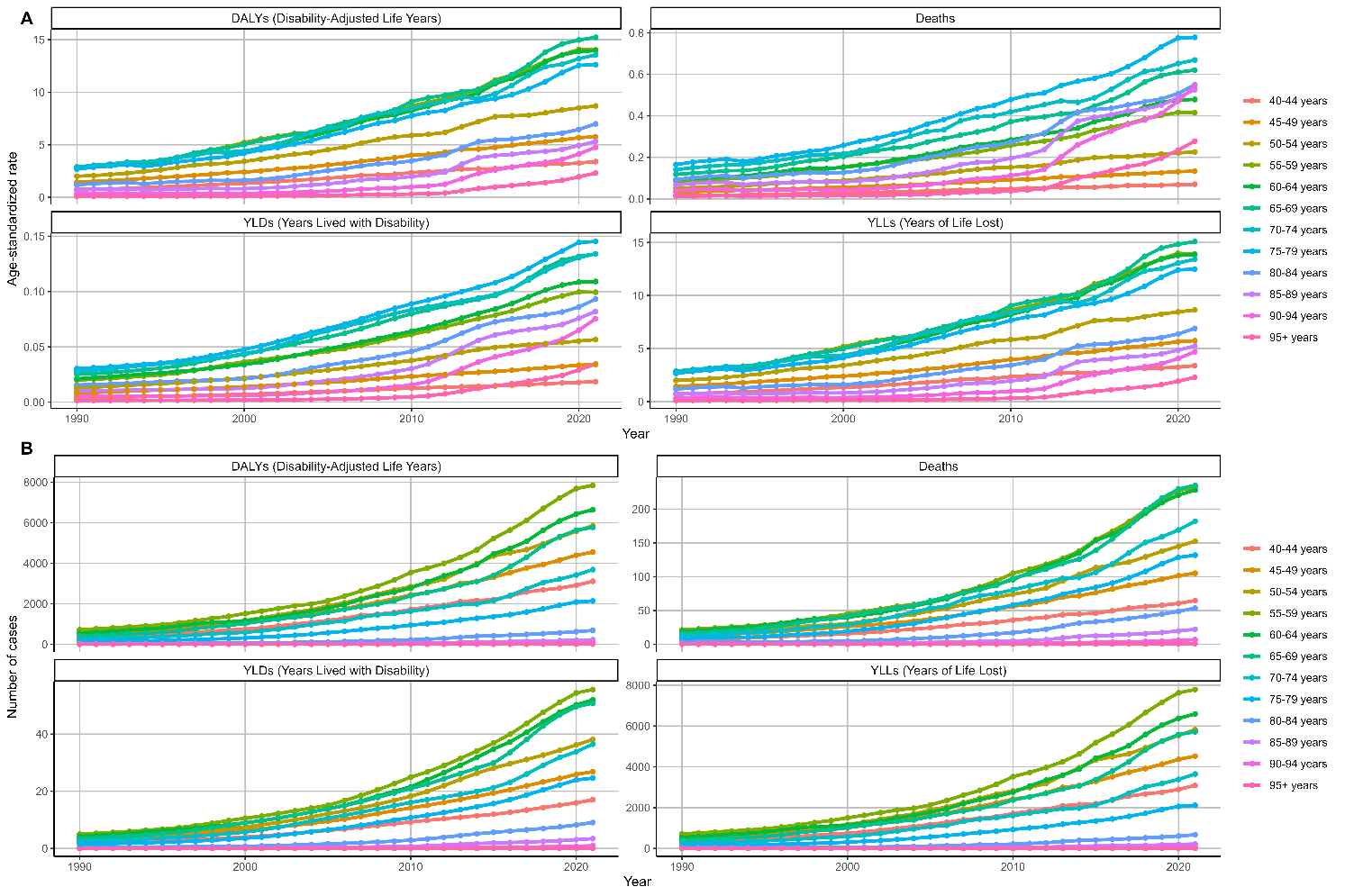
Supplementary Figure 17.** Trends in the disease burden of liver cancer attributable to high body mass index (HBMI) in India by age from 1990 to 2021.

**
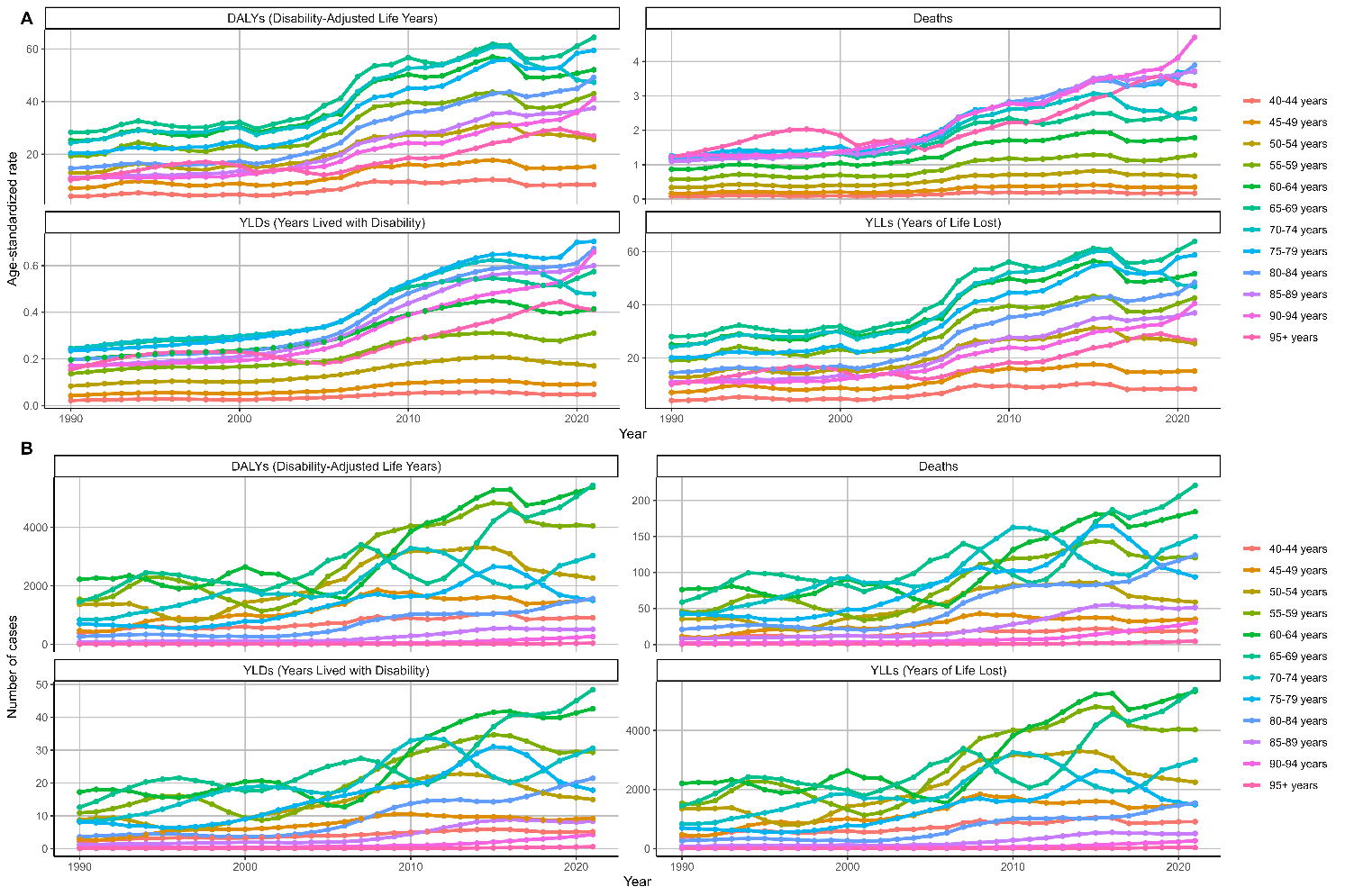
Supplementary Figure 18.** Trends in the disease burden of liver cancer attributable to high body mass index (HBMI) in Russian by age from 1990 to 2021.

**
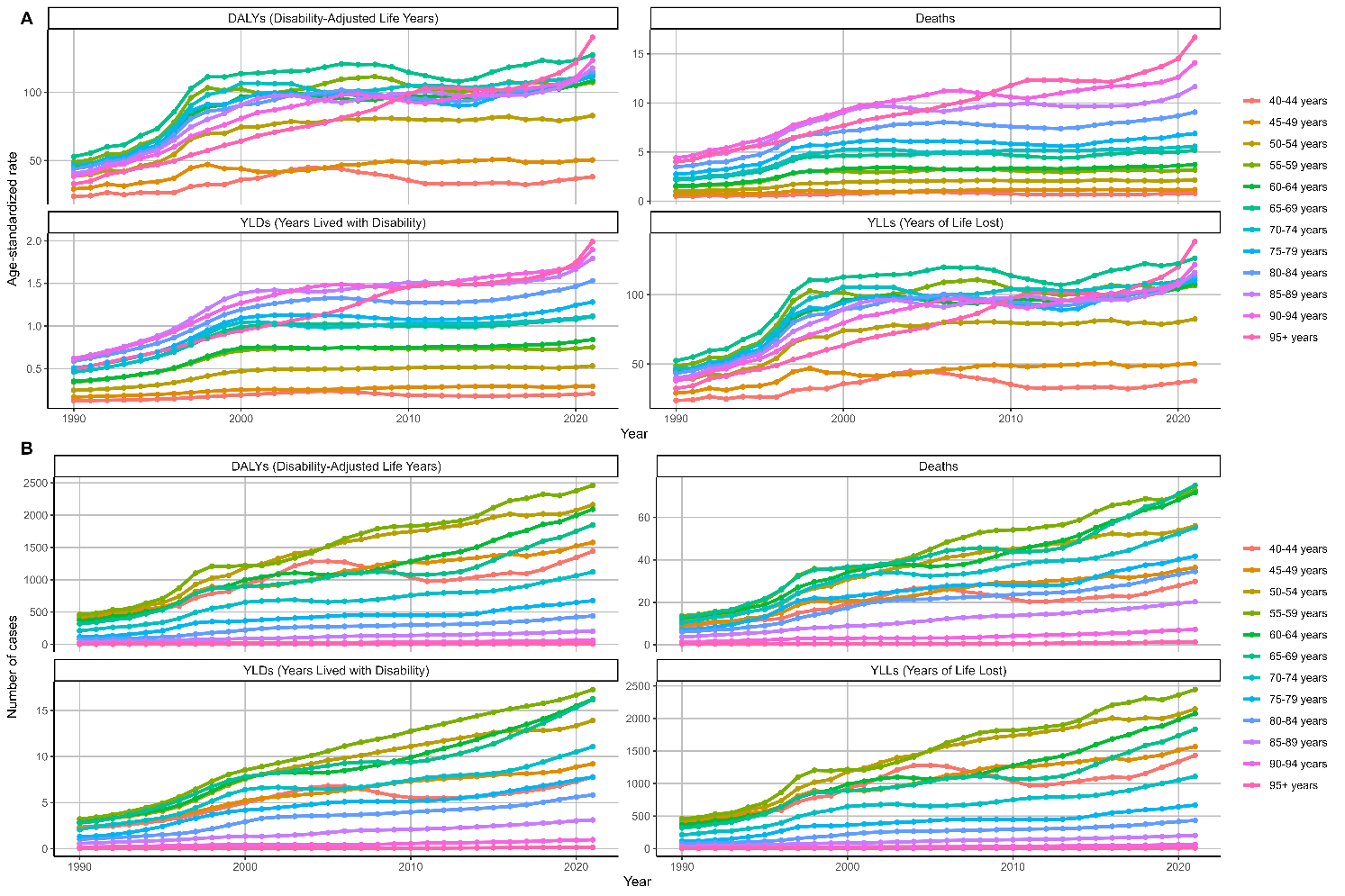
Supplementary Figure 19.** Trends in the disease burden of liver cancer attributable to high body mass index (HBMI) in South Africa by age from 1990 to 2021.

**
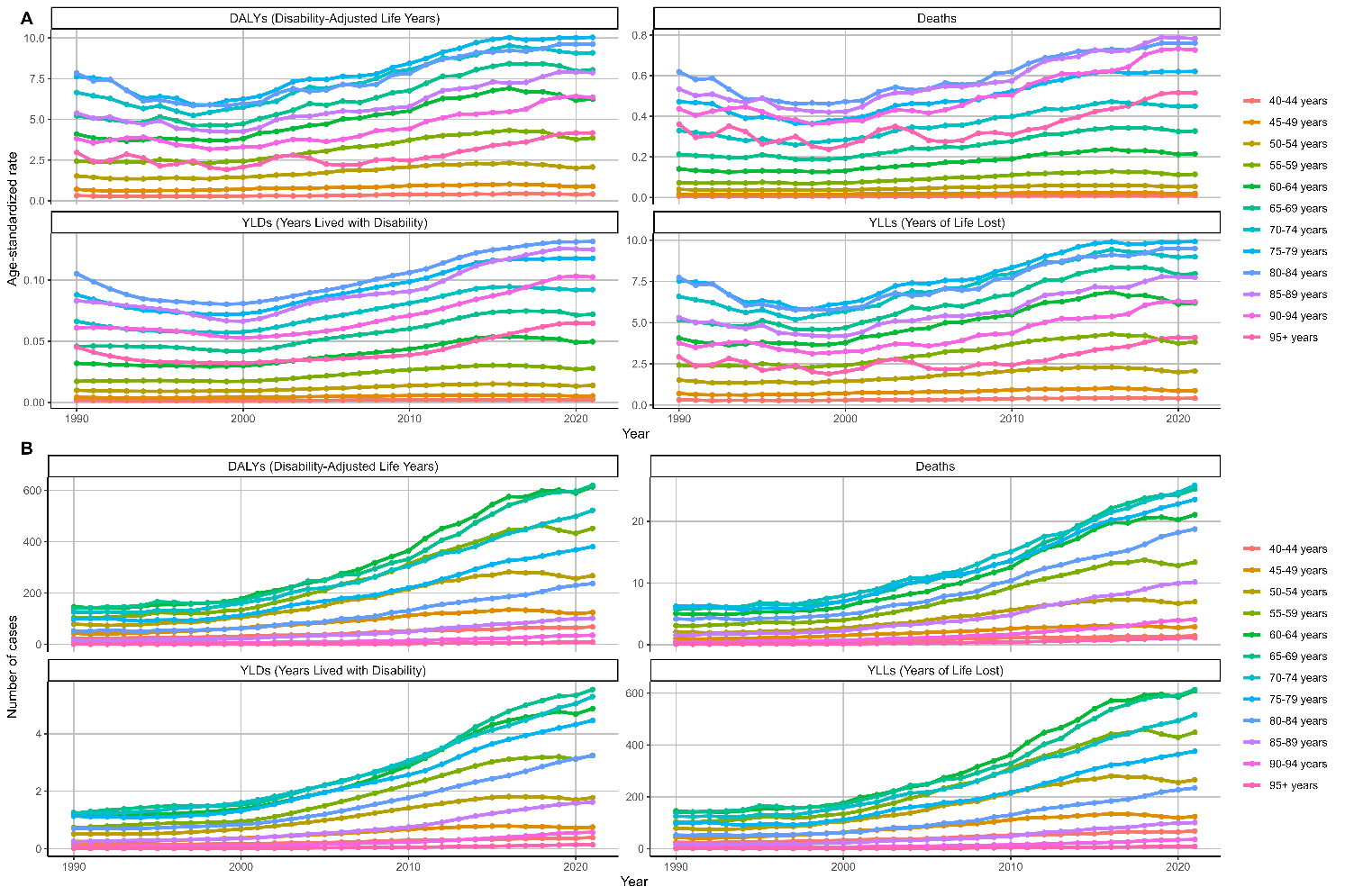
Supplementary Figure 20.** Trends in the disease burden of liver cancer attributable to high fasting plasma glucose (HFPG) in Brazil by age from 1990 to 2021.

**
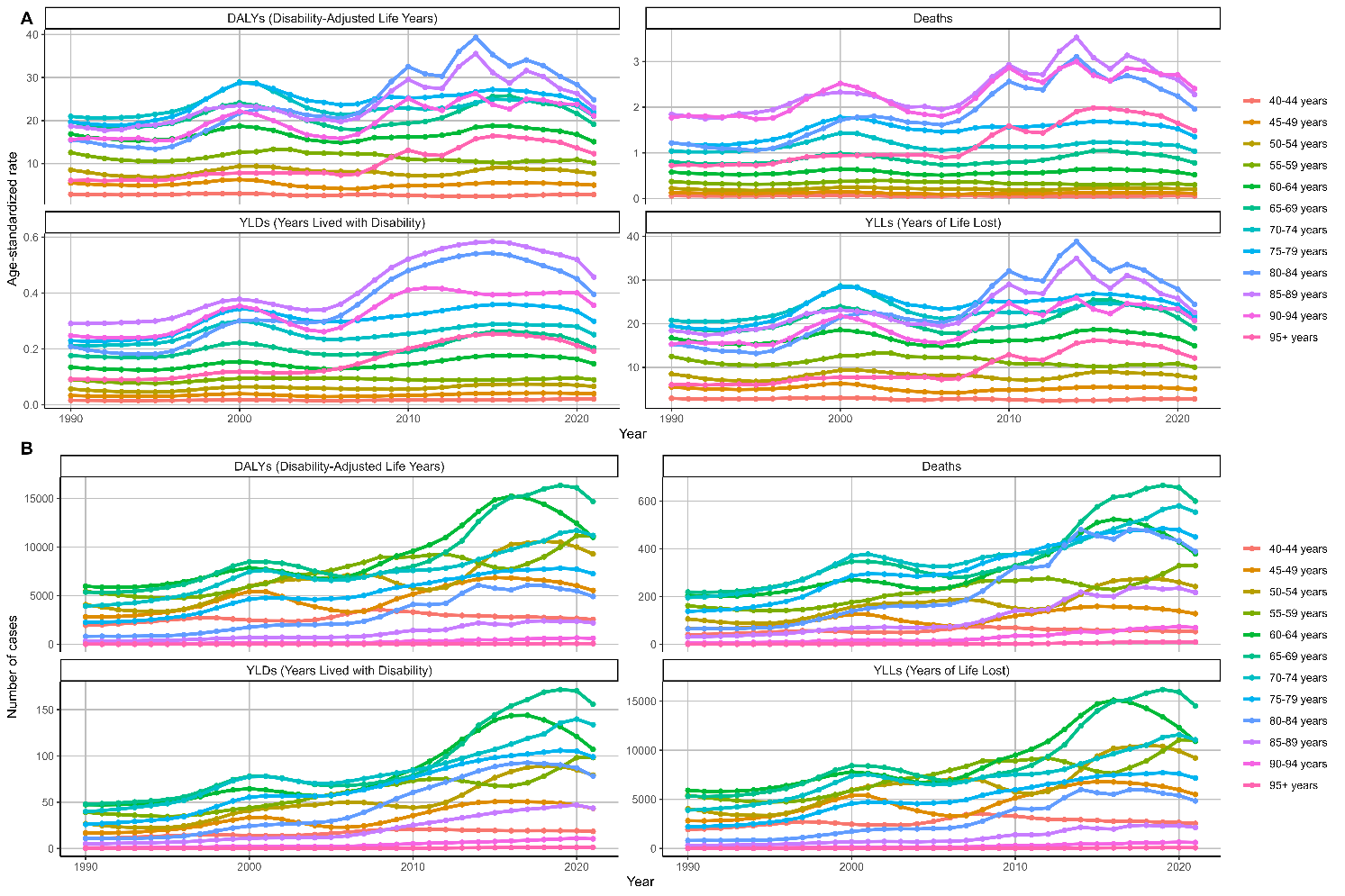
Supplementary Figure 21.** Trends in the disease burden of liver cancer attributable to high fasting plasma glucose (HFPG) in China by age from 1990 to 2021.

**
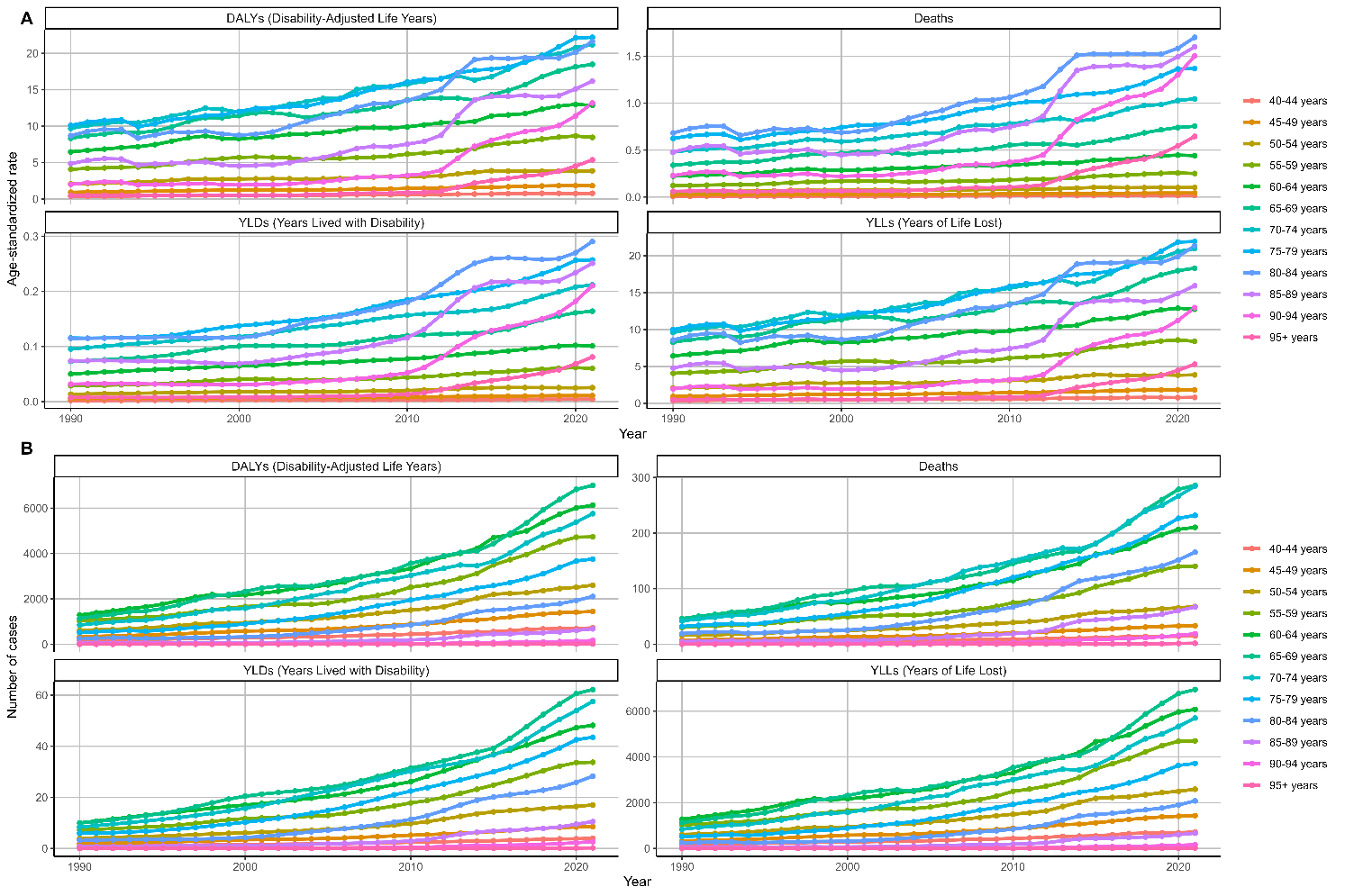
Supplementary Figure 22.** Trends in the disease burden of liver cancer attributable to high fasting plasma glucose (HFPG) in India by age from 1990 to 2021.

**
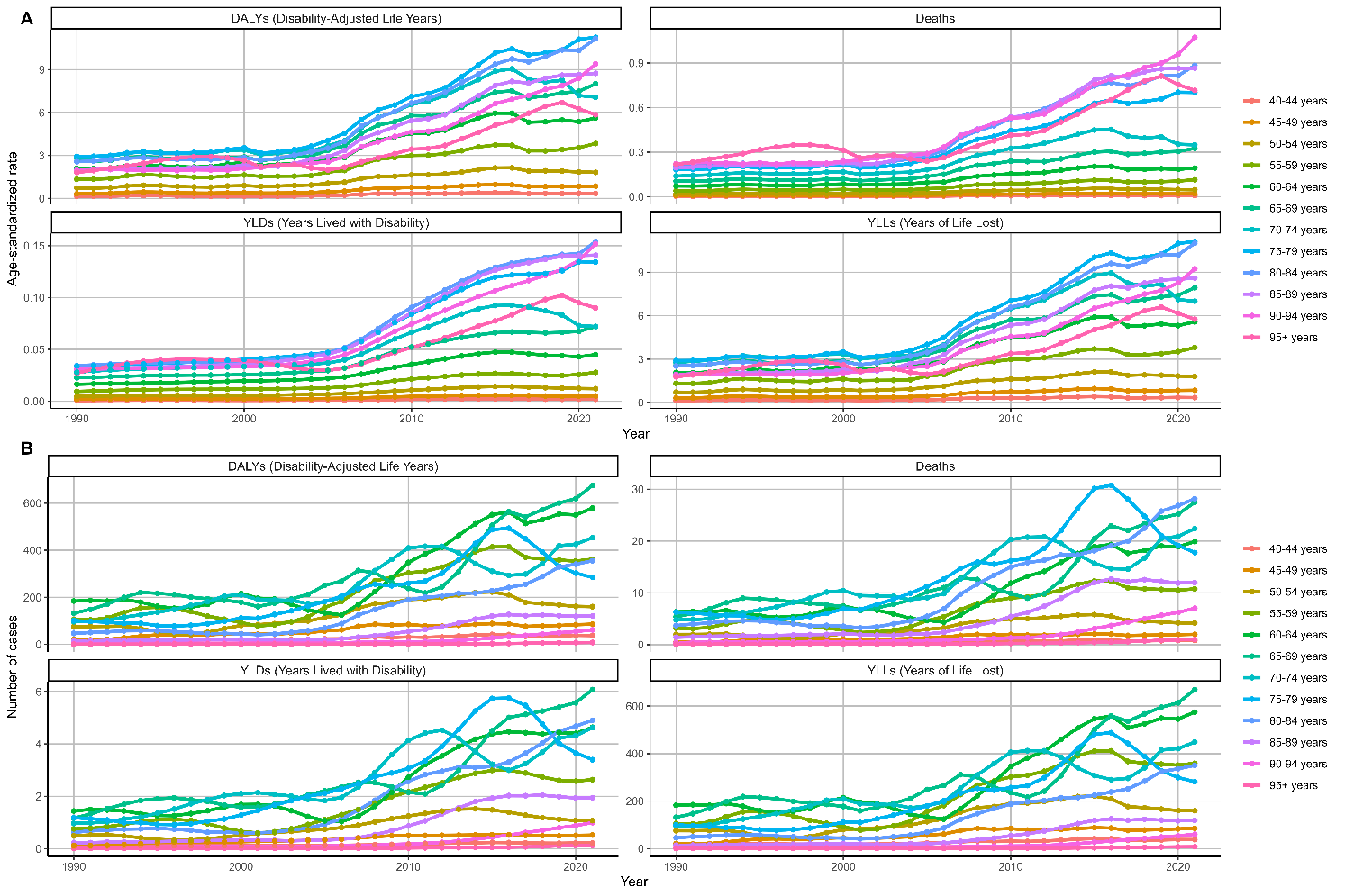
Supplementary Figure 23.** Trends in the disease burden of liver cancer attributable to high fasting plasma glucose (HFPG) in Russian by age from 1990 to 2021.

**
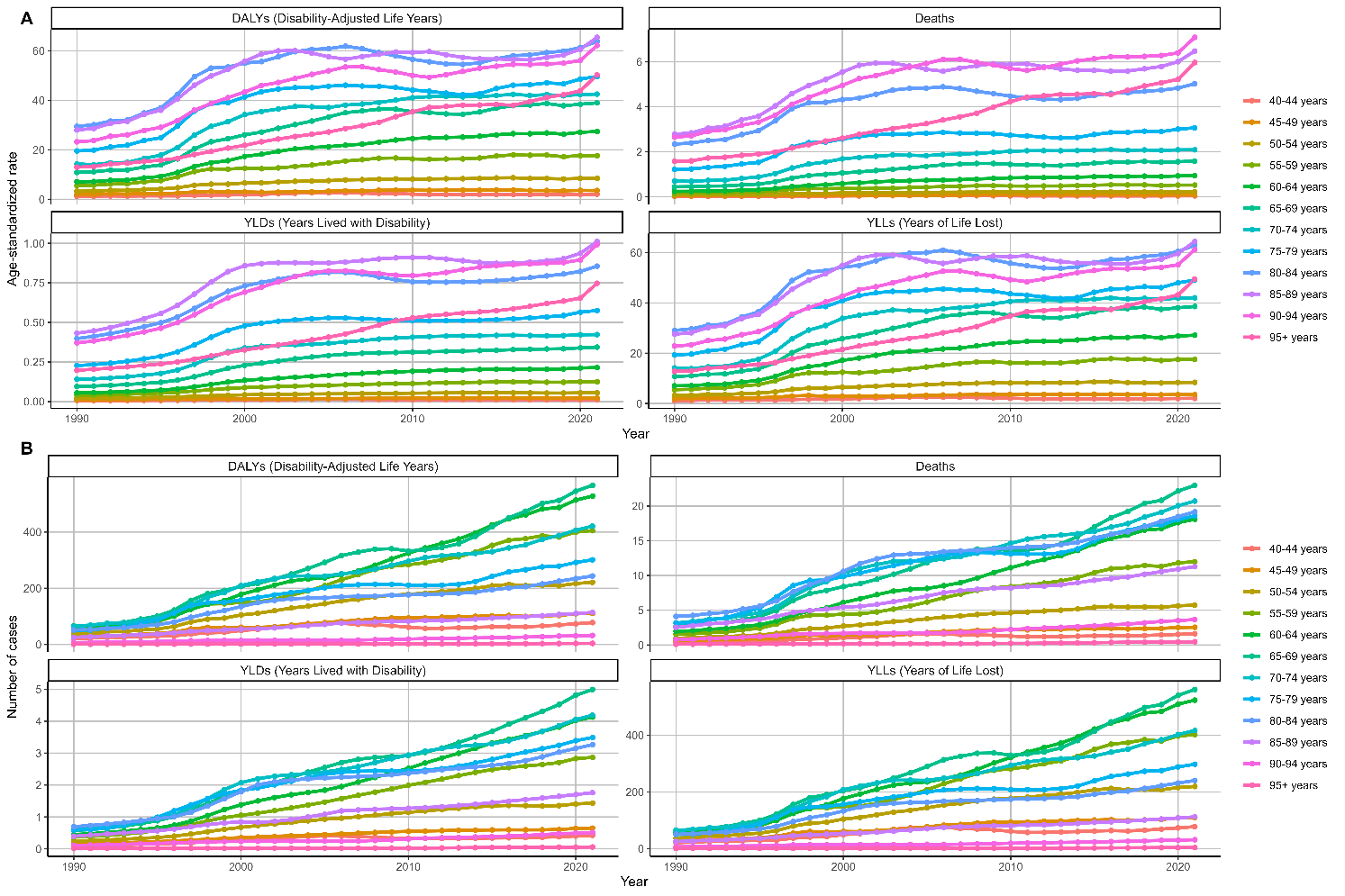
Supplementary Figure 24.** Trends in the disease burden of liver cancer attributable to high fasting plasma glucose (HFPG) in South Africa by age from 1990 to 2021.

**
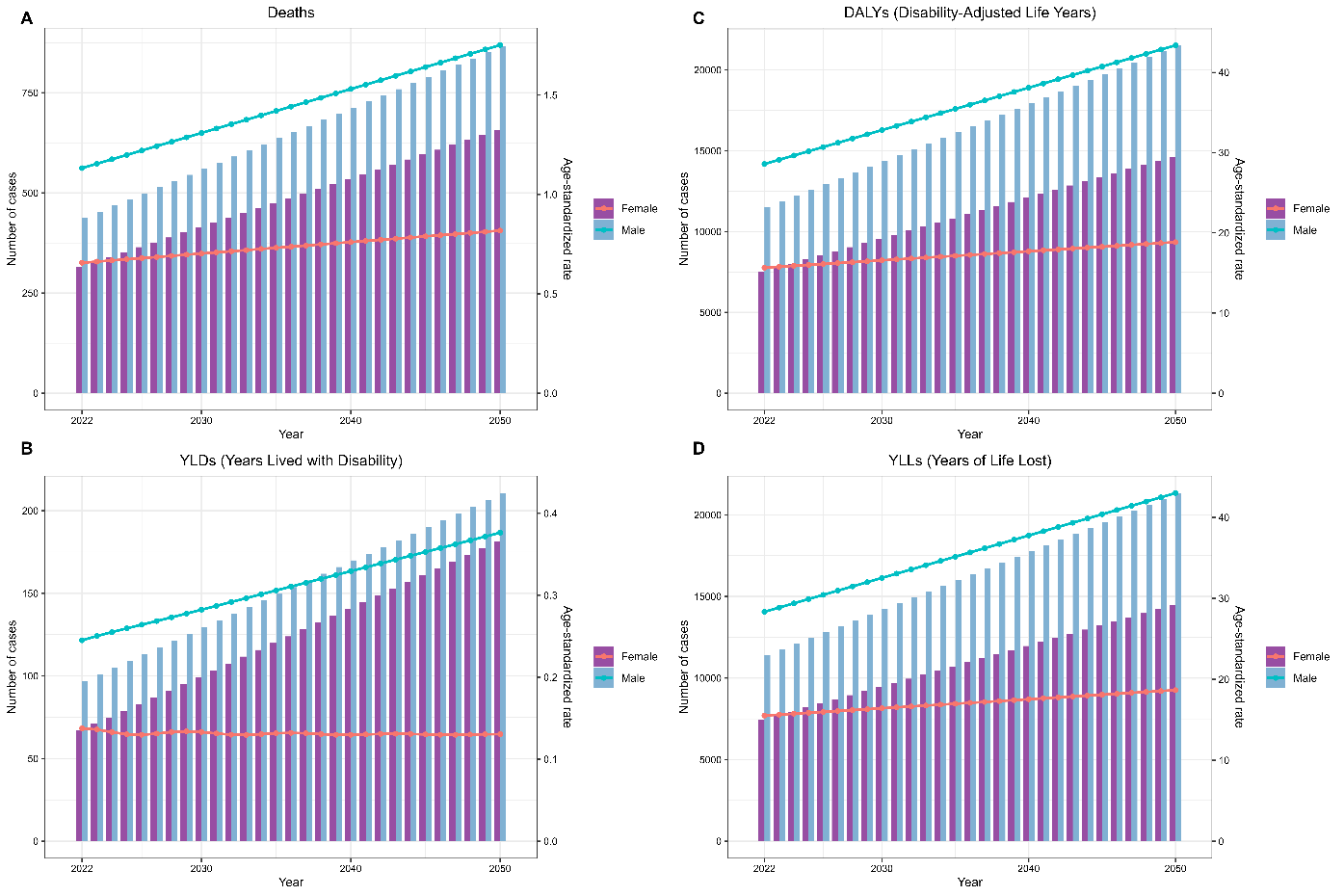
**

**Supplementary Figure 25.** Projections to 2050 of the disease burden of liver cancer attributable to high body mass index (HBMI) in Brazil performed using the Autoregressive Integrated Moving Average (ARIMA) Model.

**
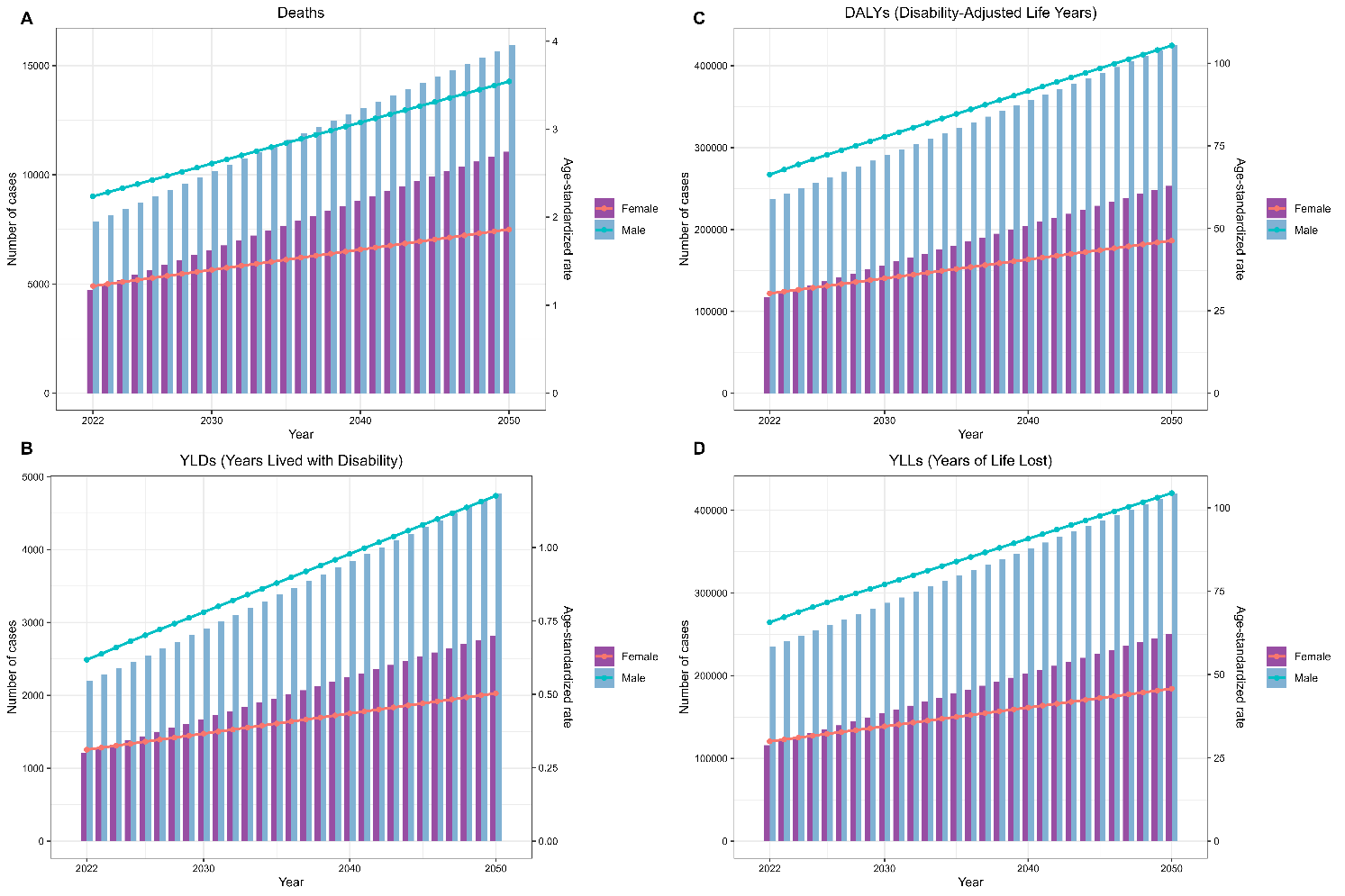
**

**Supplementary Figure 26.** Projections to 2050 of the disease burden of liver cancer attributable to high body mass index (HBMI) in China performed using the Autoregressive Integrated Moving Average (ARIMA) Model.

**
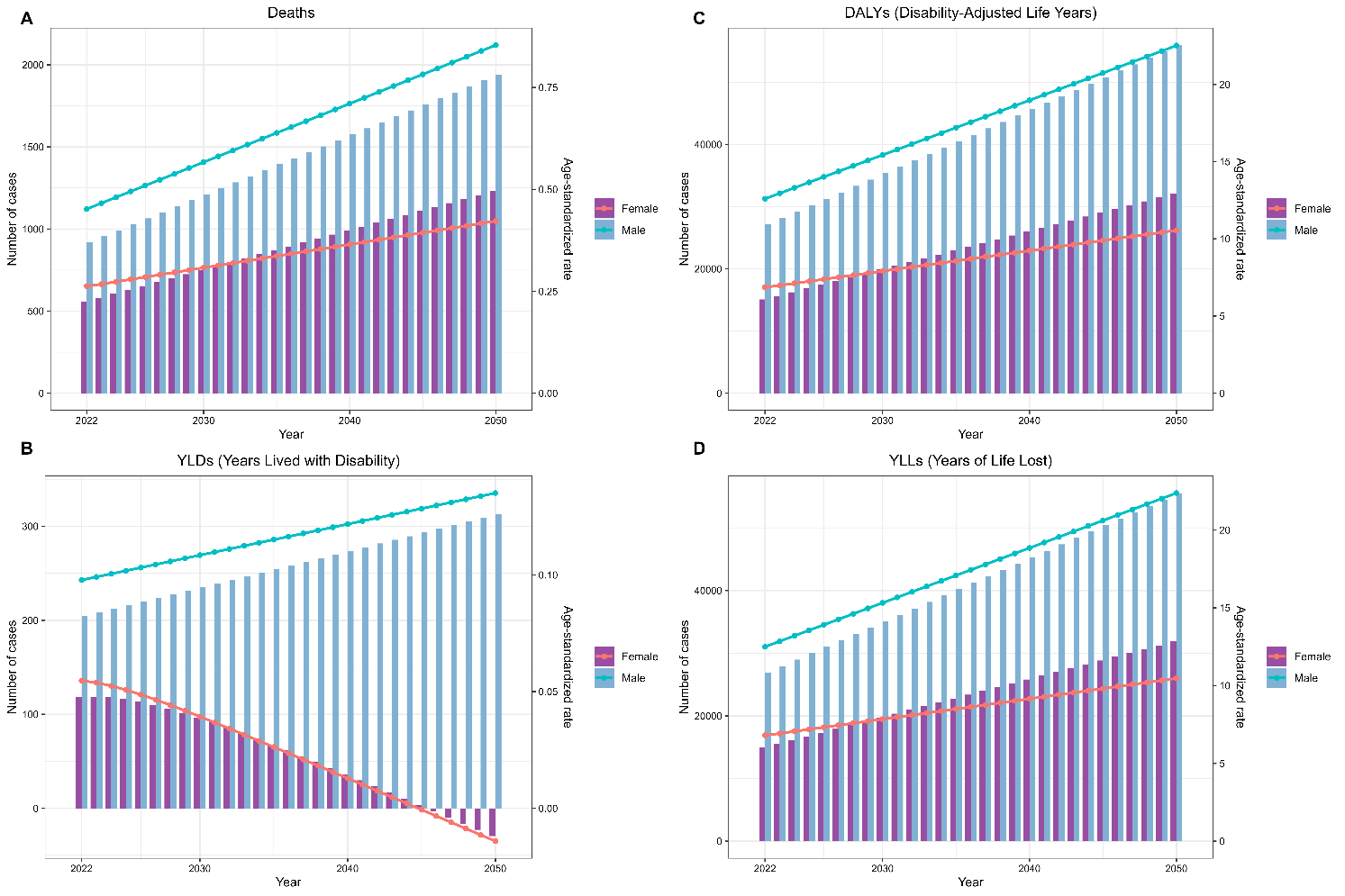
**

**Supplementary Figure 27.** Projections to 2050 of the disease burden of liver cancer attributable to high body mass index (HBMI) in India performed using the Autoregressive Integrated Moving Average (ARIMA) Model.

**
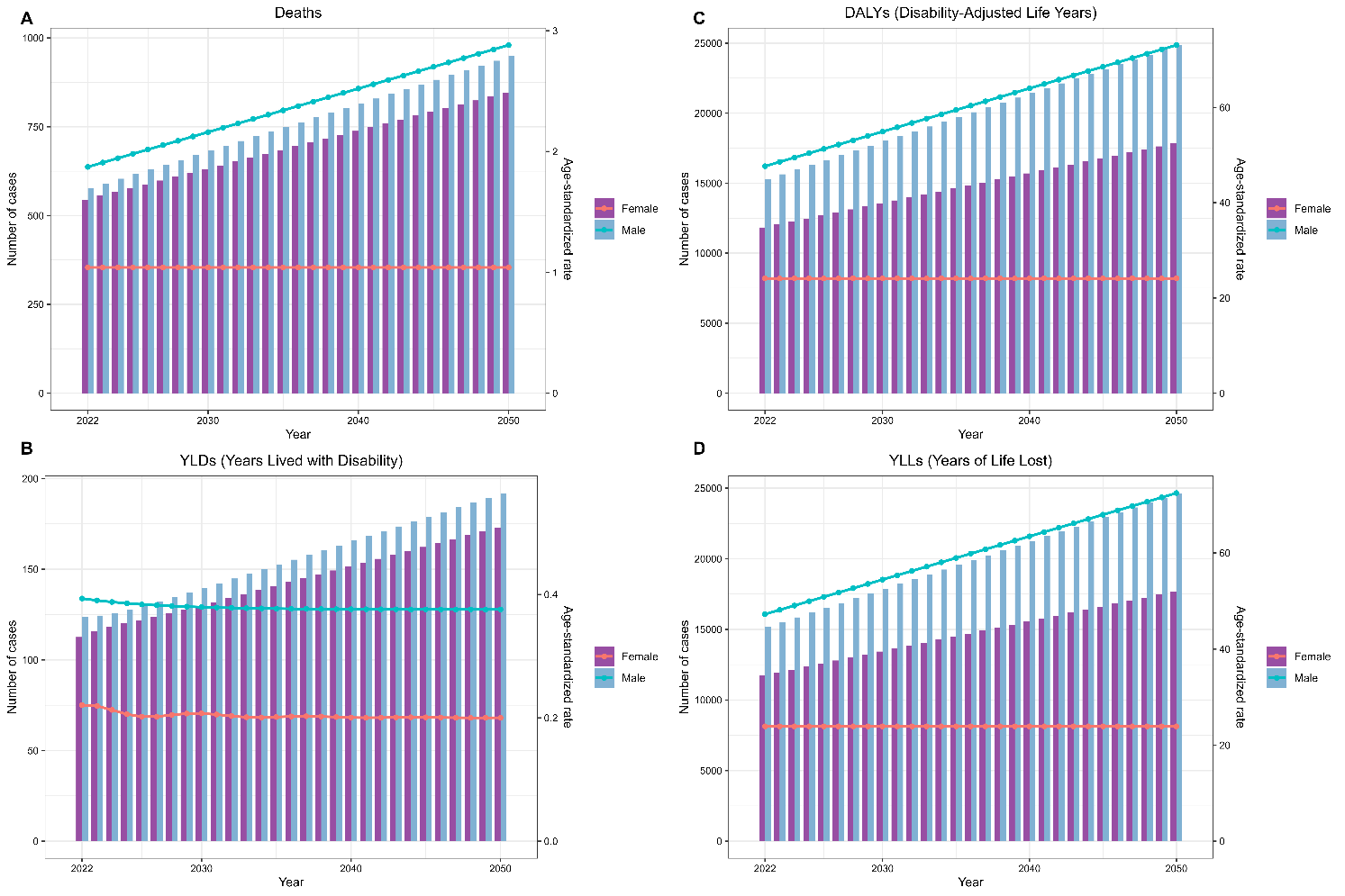
**

**Supplementary Figure 28.** Projections to 2050 of the disease burden of liver cancer attributable to high body mass index (HBMI) in Russian performed using the Autoregressive Integrated Moving Average (ARIMA) Model.

**
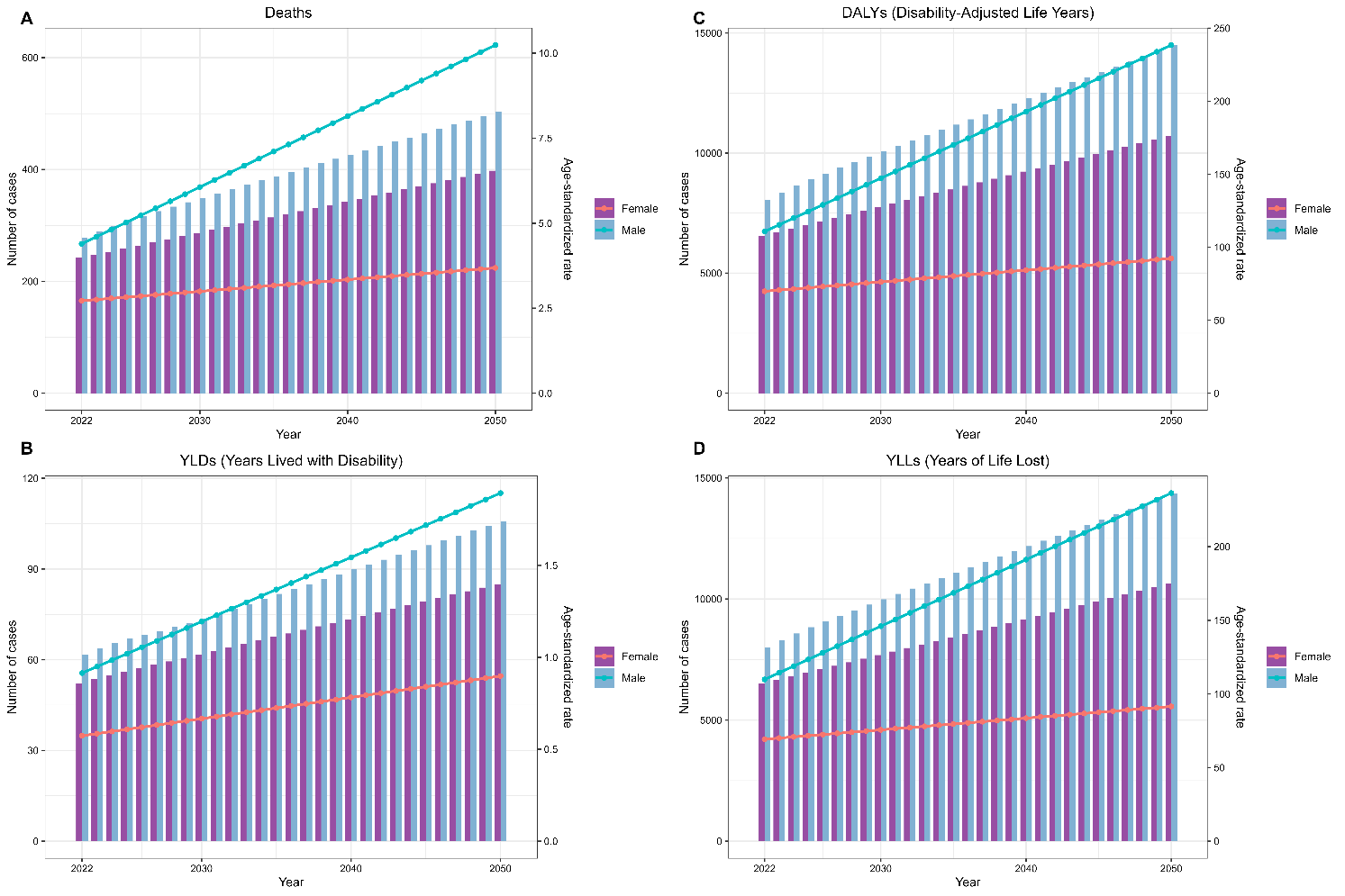
**

**Supplementary Figure 29.** Projections to 2050 of the disease burden of liver cancer attributable to high body mass index (HBMI) in South Africa performed using the Autoregressive Integrated Moving Average (ARIMA) Model.

**
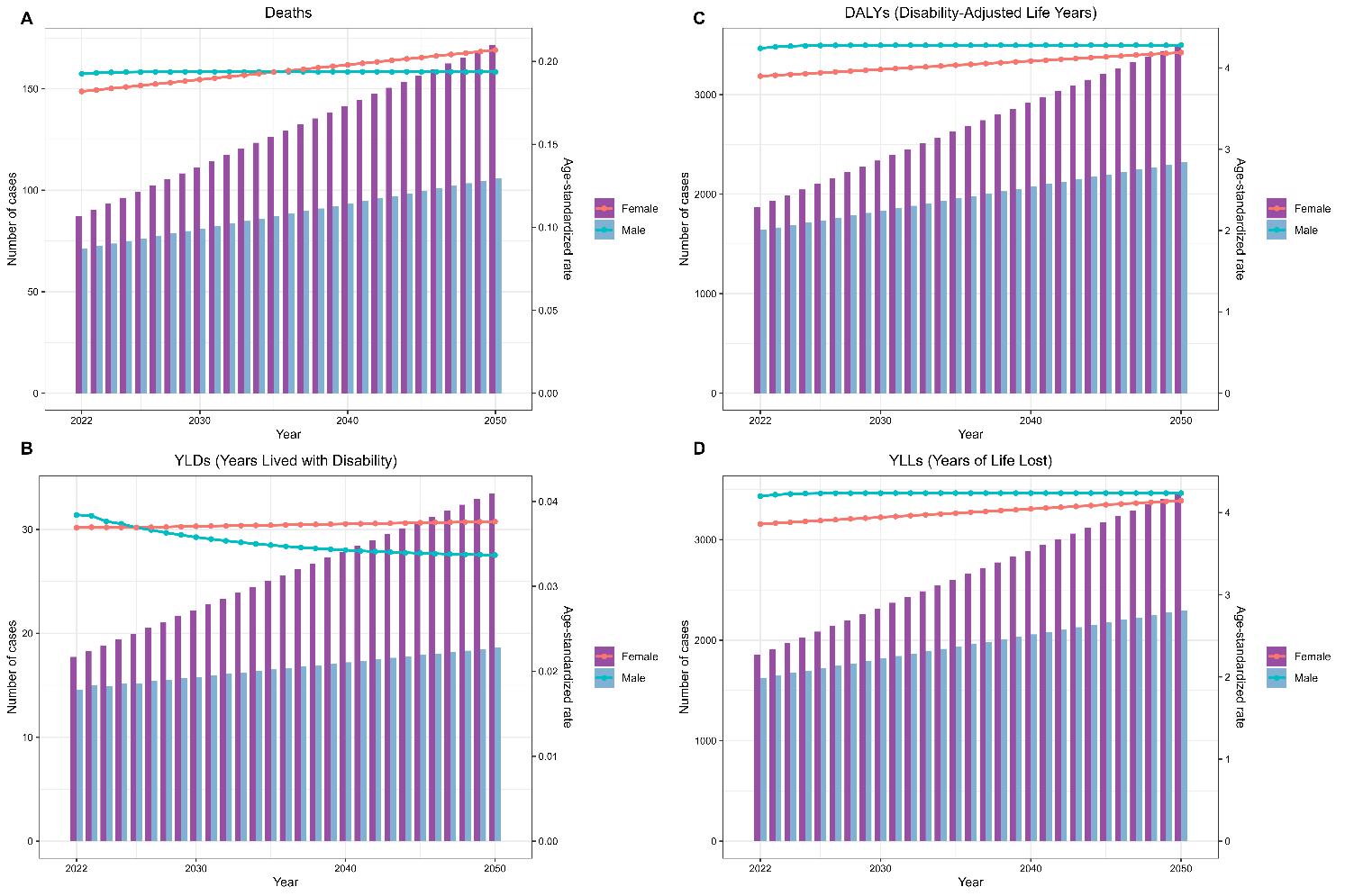
**

**Supplementary Figure 30.** Projections to 2050 of the disease burden of liver cancer attributable to high fasting plasma glucose (HFPG) in Brazil performed using the Autoregressive Integrated Moving Average (ARIMA) Model.

**
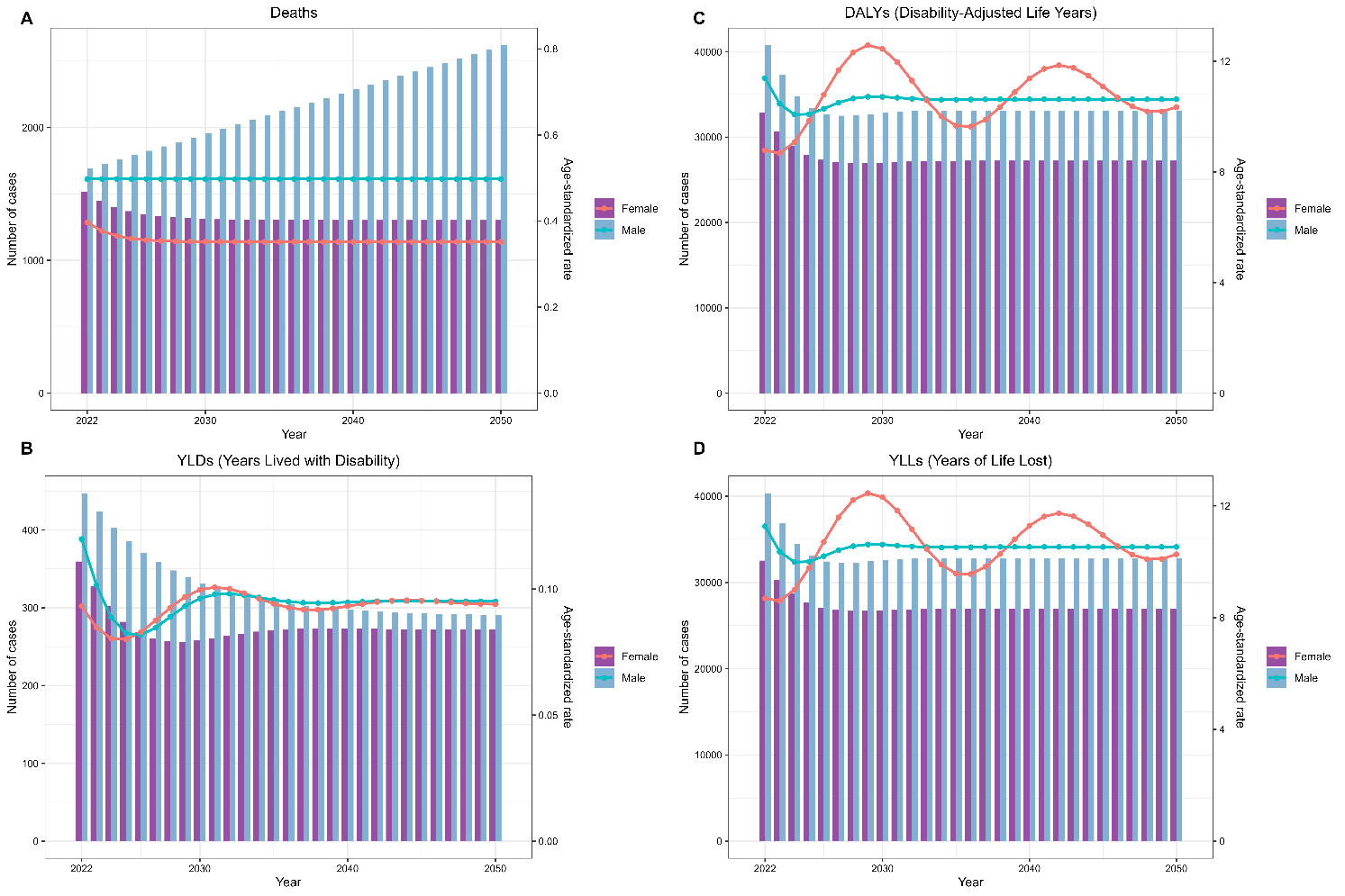
**

**Supplementary Figure 31.** Projections to 2050 of the disease burden of liver cancer attributable to high fasting plasma glucose (HFPG) in China performed using the Autoregressive Integrated Moving Average (ARIMA) Model.

**
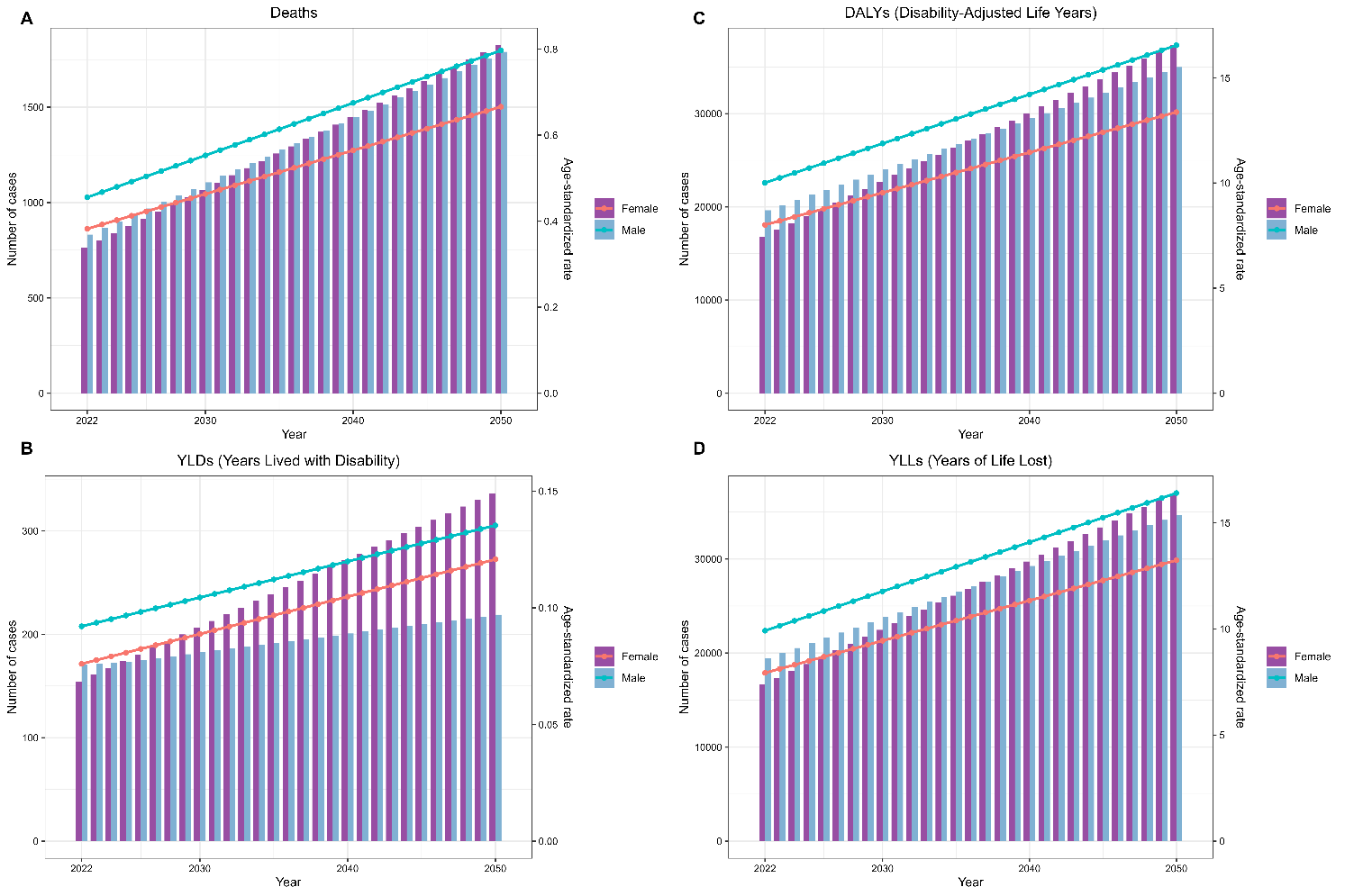
**

**Supplementary Figure 32.** Projections to 2050 of the disease burden of liver cancer attributable to high fasting plasma glucose (HFPG) in India performed using the Autoregressive Integrated Moving Average (ARIMA) Model.

**
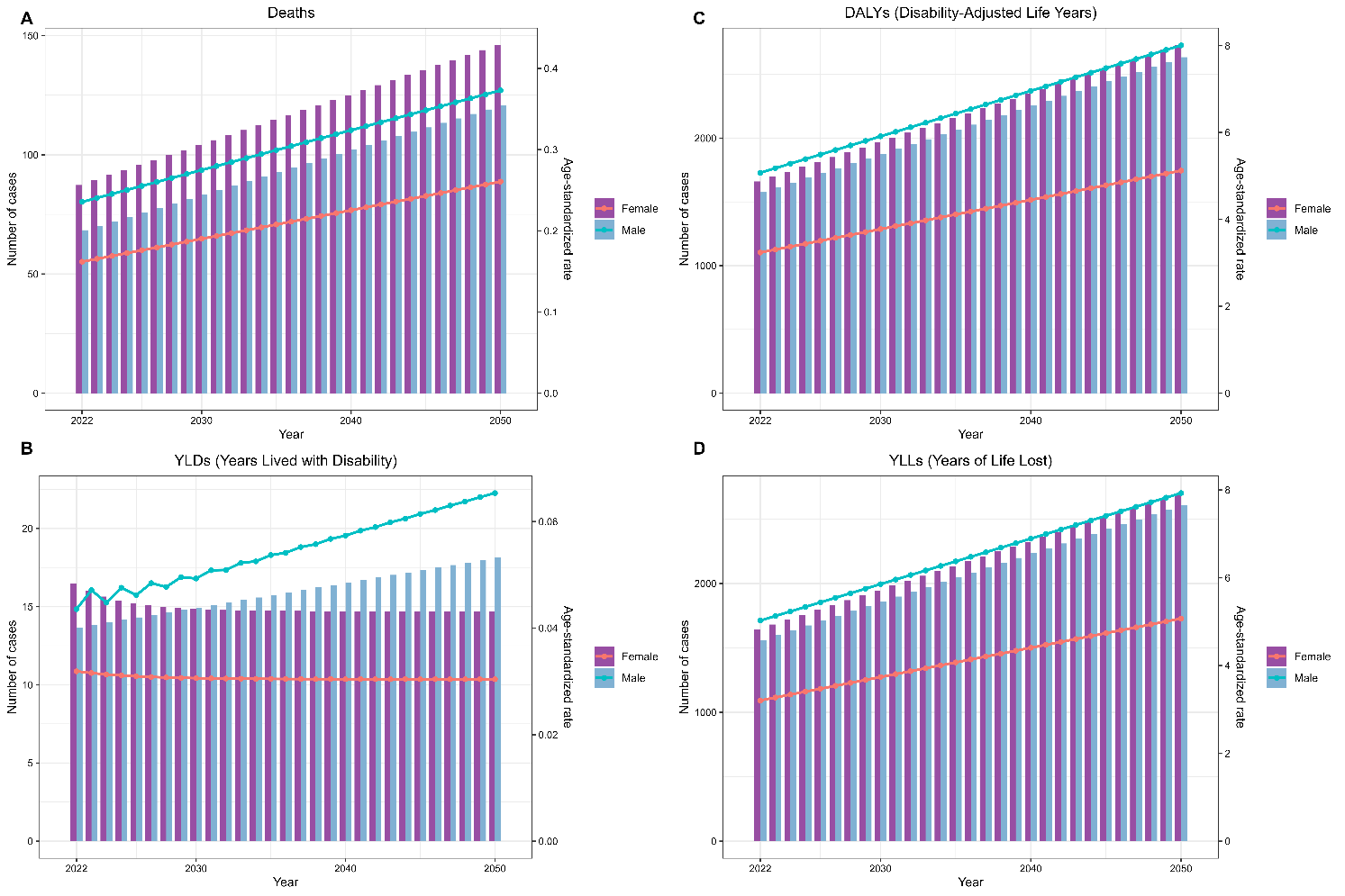
**

**Supplementary Figure 33.** Projections to 2050 of the disease burden of liver cancer attributable to high fasting plasma glucose (HFPG) in Russian performed using the Autoregressive Integrated Moving Average (ARIMA) Model.

**
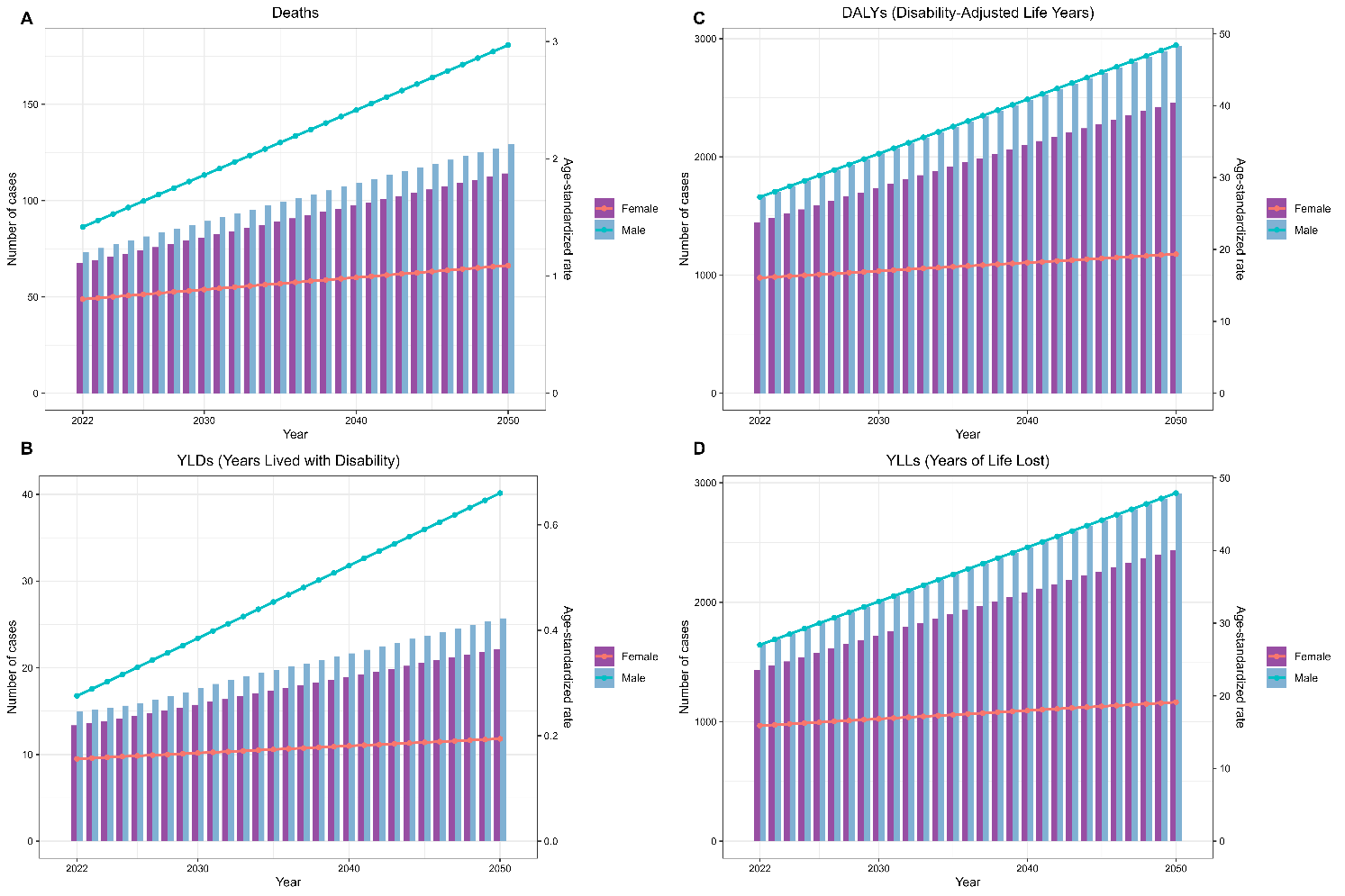
**

**Supplementary Figure 34.** Projections to 2050 of the disease burden of liver cancer attributable to high fasting plasma glucose (HFPG) in South Africa performed using the Autoregressive Integrated Moving Average (ARIMA) Model.

**
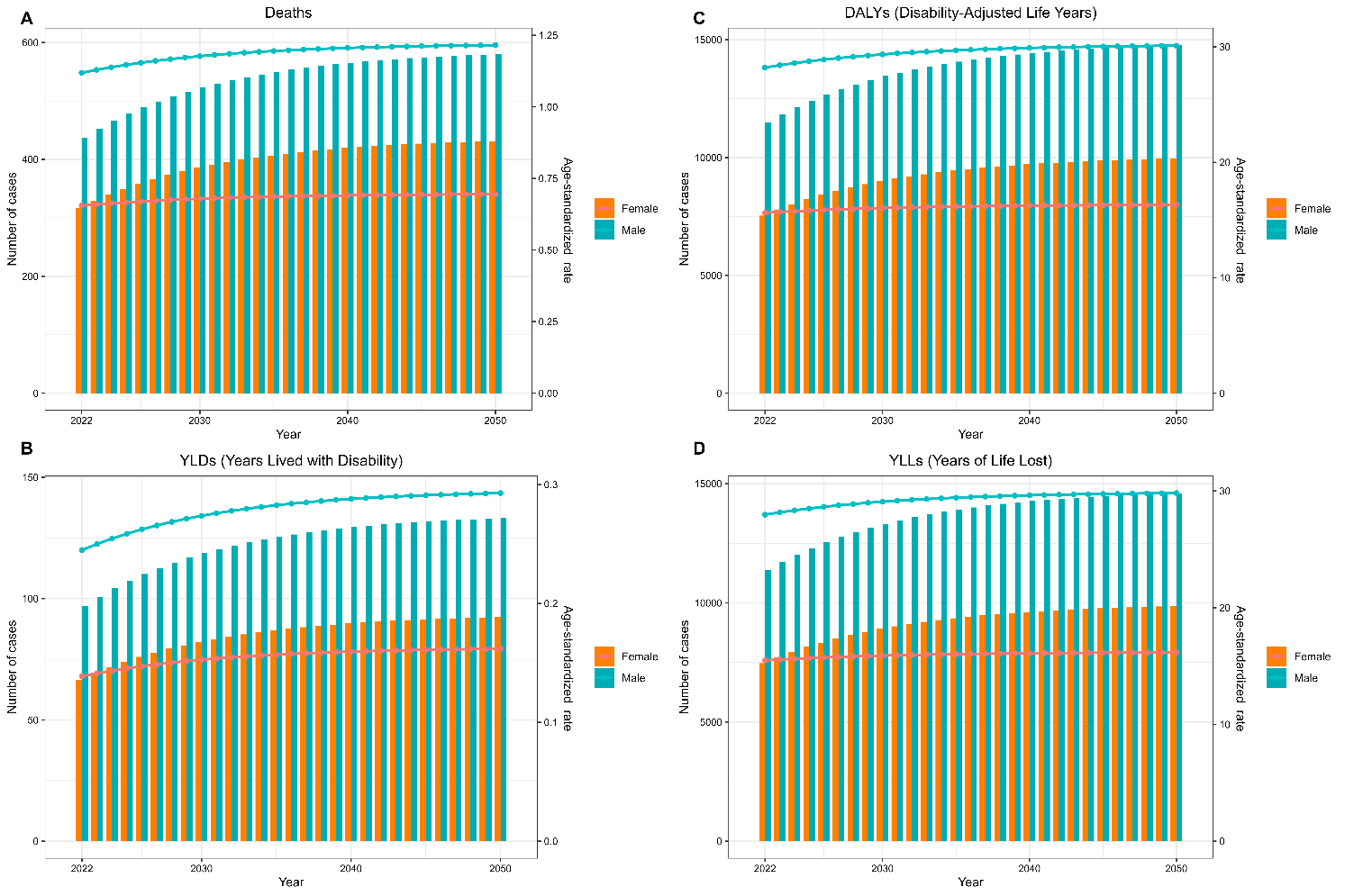
Supplementary Figure 35.** Projections to 2050 of the global burden of liver cancer attributable to high body mass index (HBMI) in Brazil performed using the Exponential Smoothing (ES) Model.

**
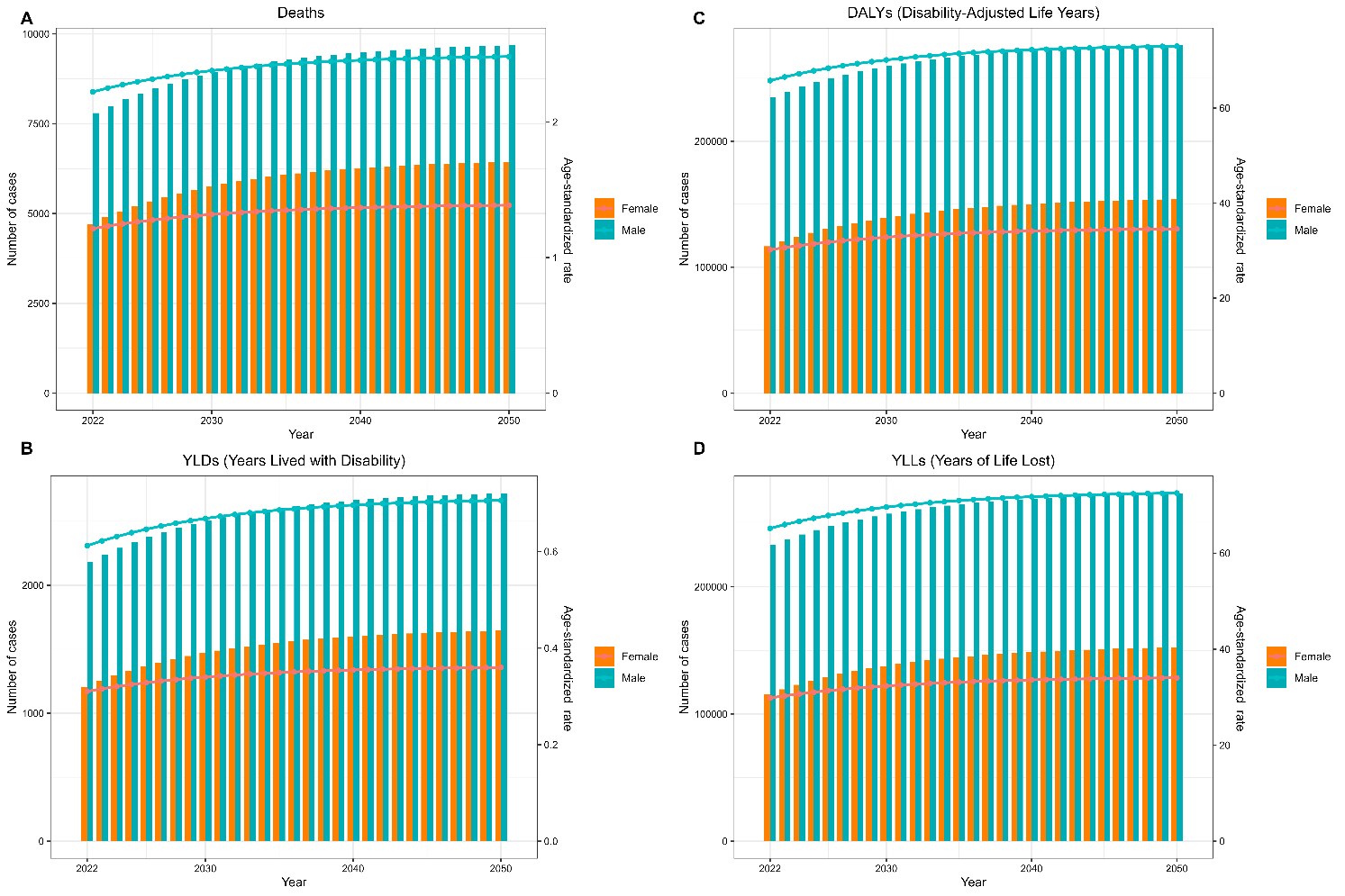
Supplementary Figure 36.** Projections to 2050 of the global burden of liver cancer attributable to high body mass index (HBMI) in China performed using the Exponential Smoothing (ES) Model.

**
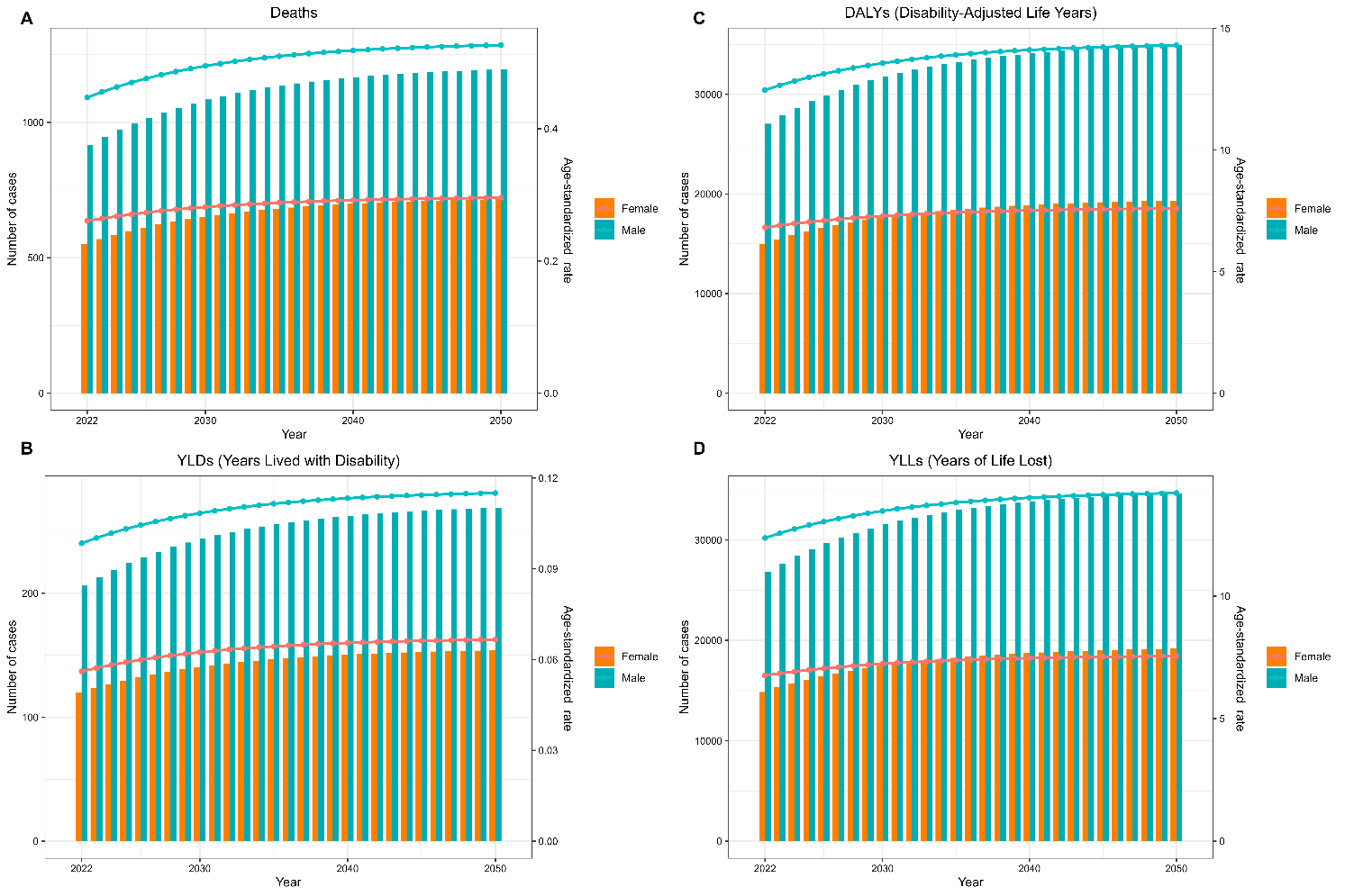
Supplementary Figure 37.** Projections to 2050 of the global burden of liver cancer attributable to high body mass index (HBMI) in India performed using the Exponential Smoothing (ES) Model.

**
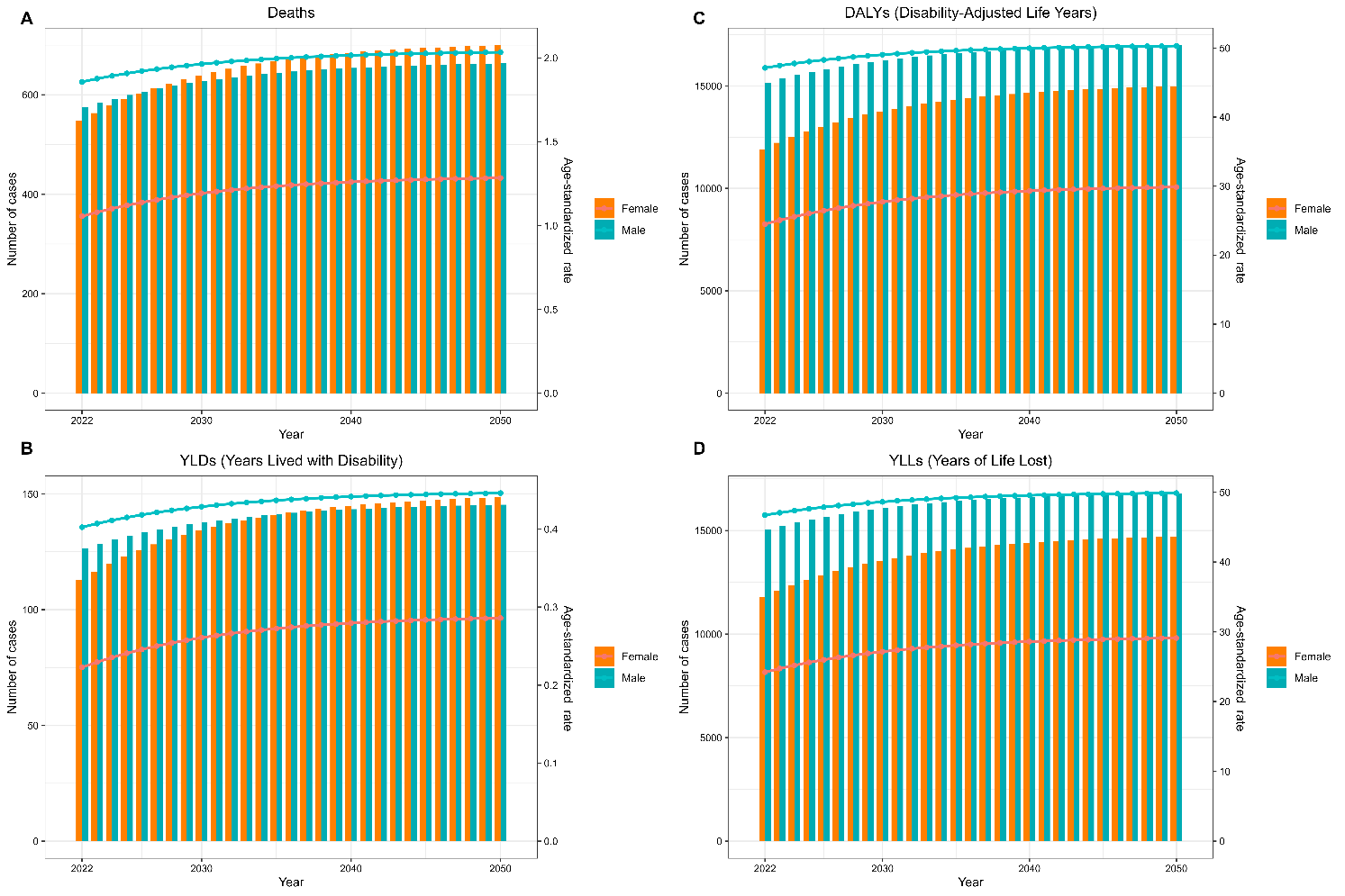
Supplementary Figure 38.** Projections to 2050 of the global burden of liver cancer attributable to high body mass index (HBMI) in Russian performed using the Exponential Smoothing (ES) Model.

**
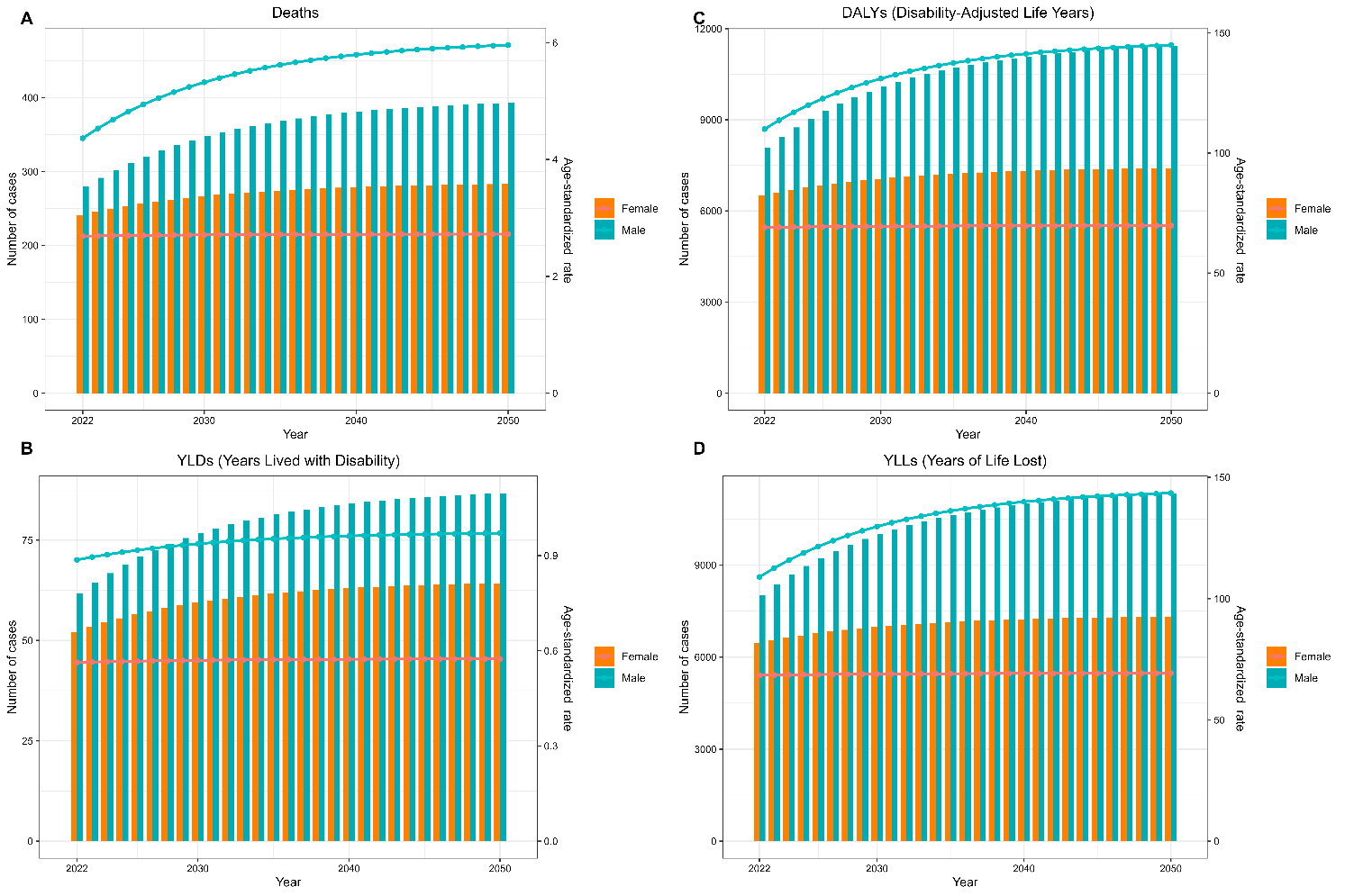
**

**Supplementary Figure 39.** Projections to 2050 of the global burden of liver cancer attributable to high body mass index (HBMI) in South Africa performed using the Exponential Smoothing (ES) Model.

**
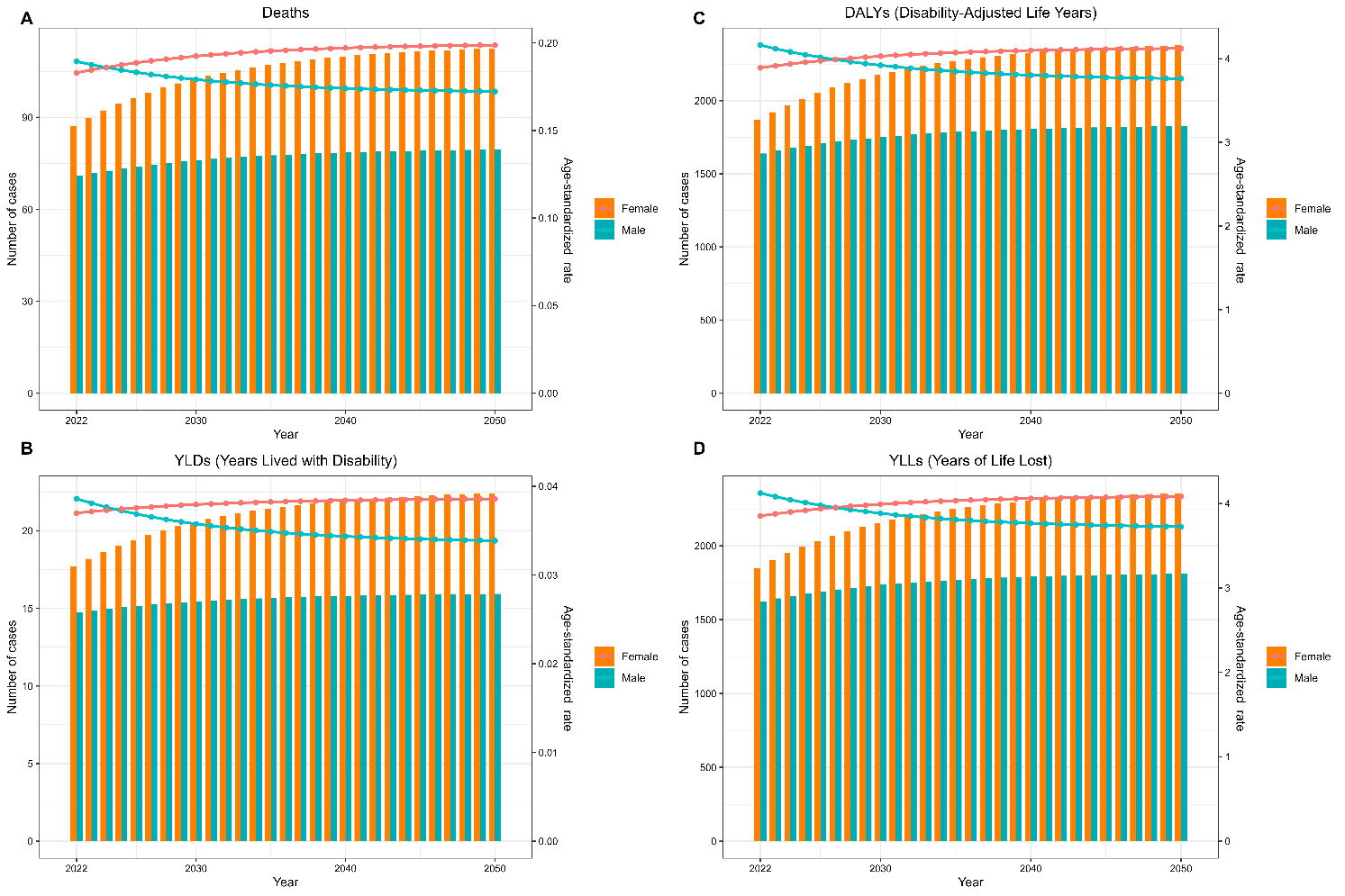
**

**Supplementary Figure 40.** Projections to 2050 of the global burden of liver cancer attributable to high fasting plasma glucose (HFPG) in Brazil performed using the Exponential Smoothing (ES) Model.

**
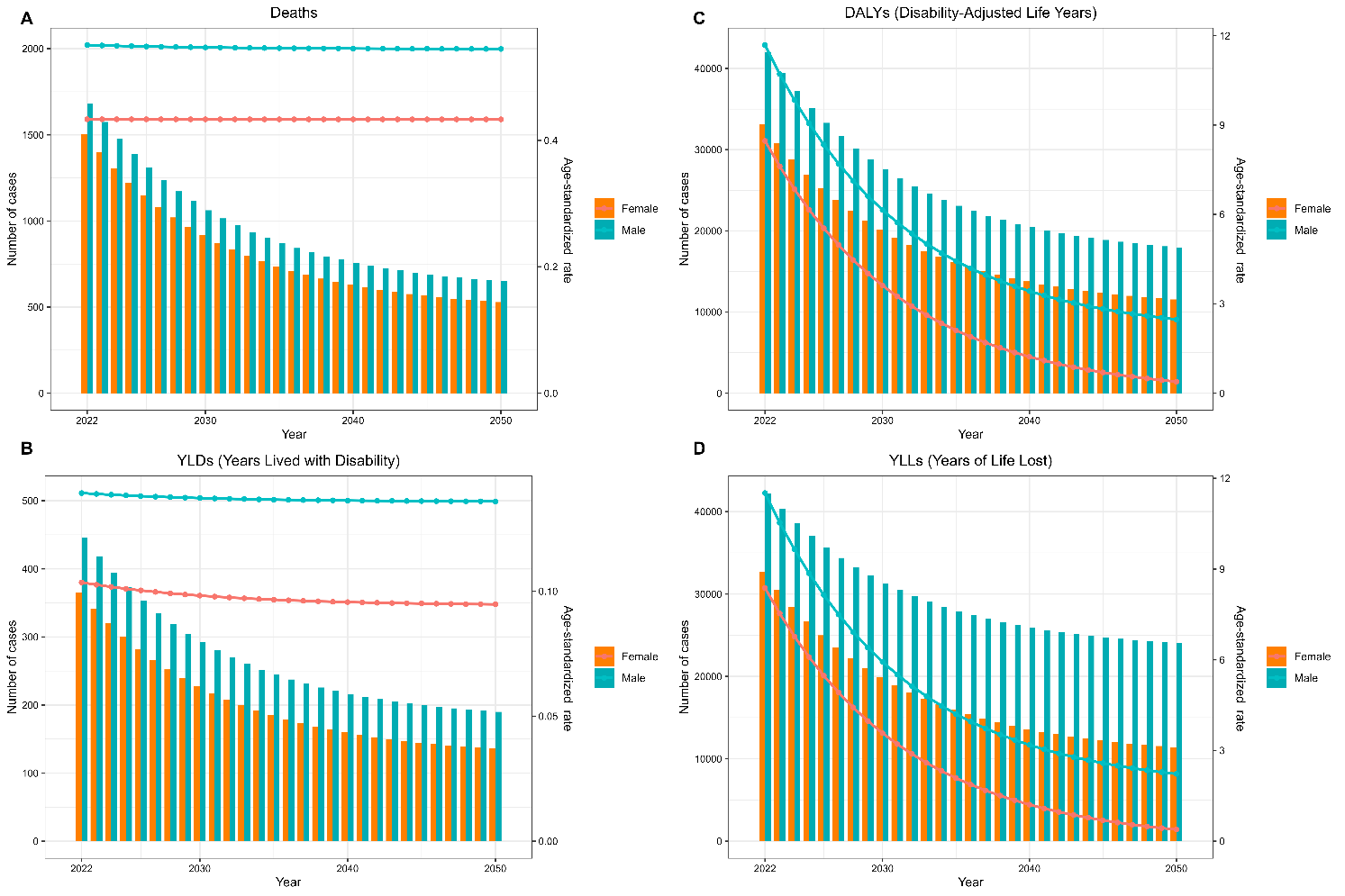
**

**Supplementary Figure 41.** Projections to 2050 of the global burden of liver cancer attributable to high fasting plasma glucose (HFPG) in China performed using the Exponential Smoothing (ES) Model.

**
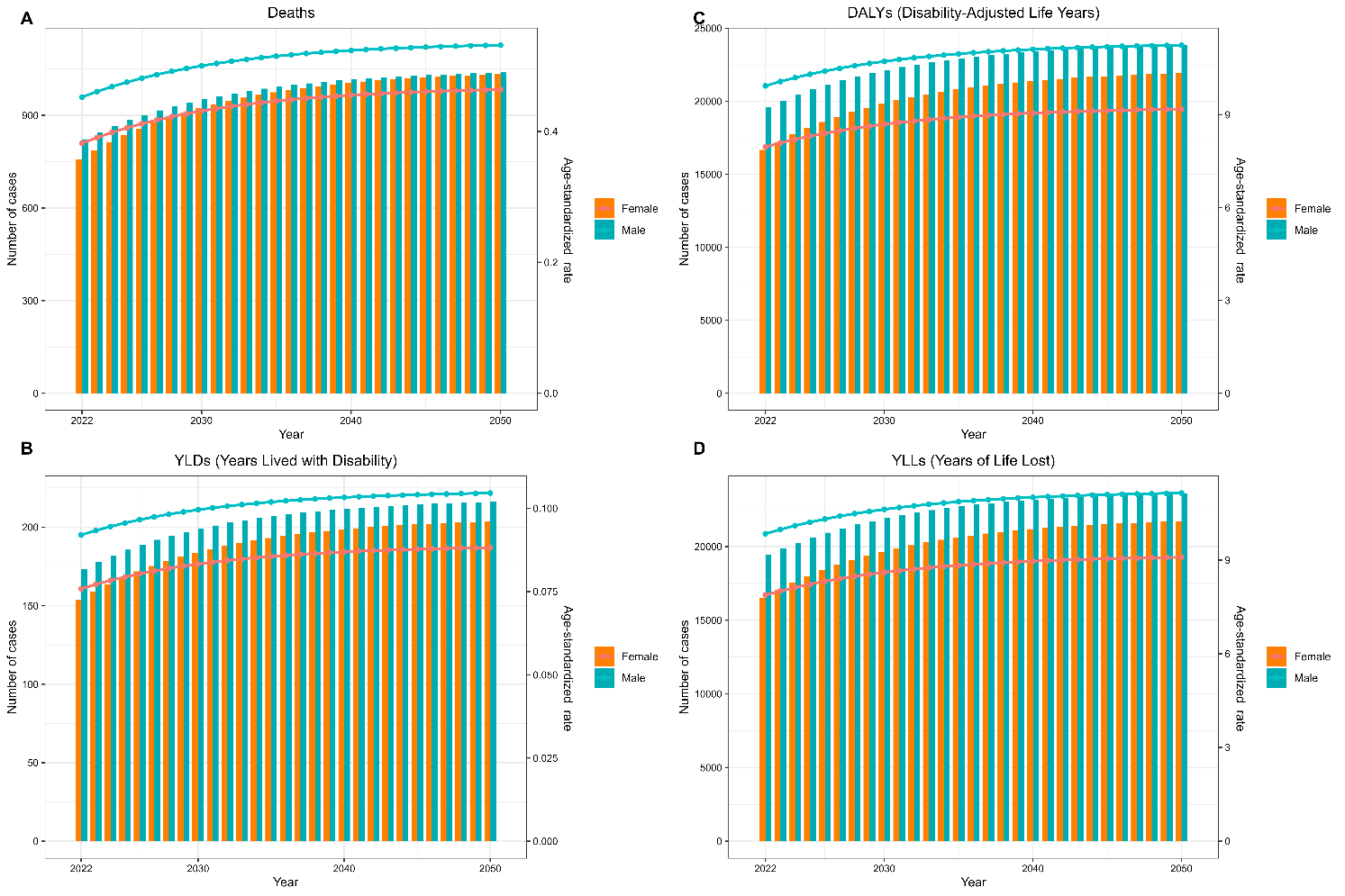
Supplementary Figure 42.** Projections to 2050 of the global burden of liver cancer attributable to high fasting plasma glucose (HFPG) in India performed using the Exponential Smoothing (ES) Model.

**
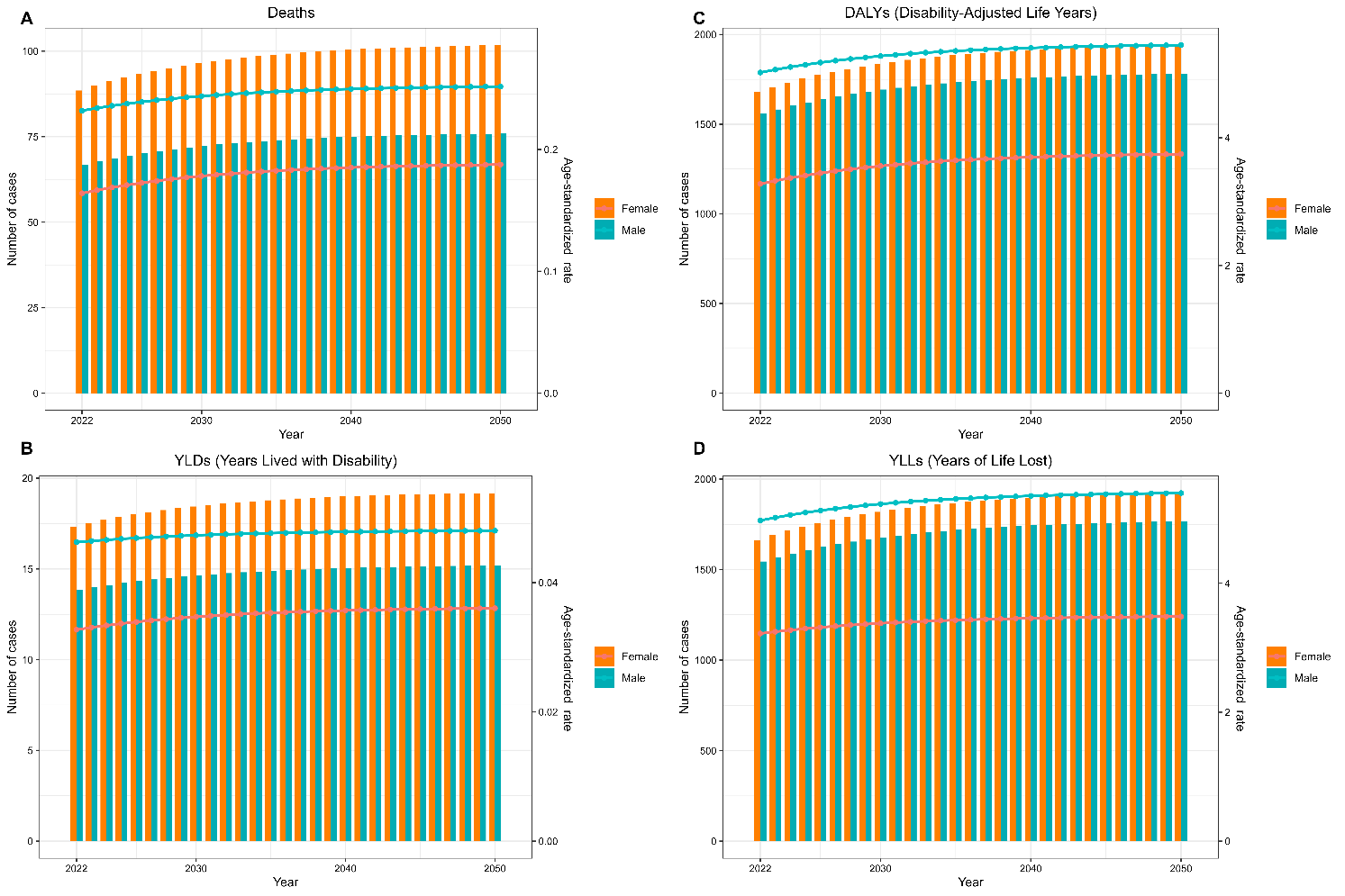
Supplementary Figure 43.** Projections to 2050 of the global burden of liver cancer attributable to high fasting plasma glucose (HFPG) in Russian performed using the Exponential Smoothing (ES) Model.

**
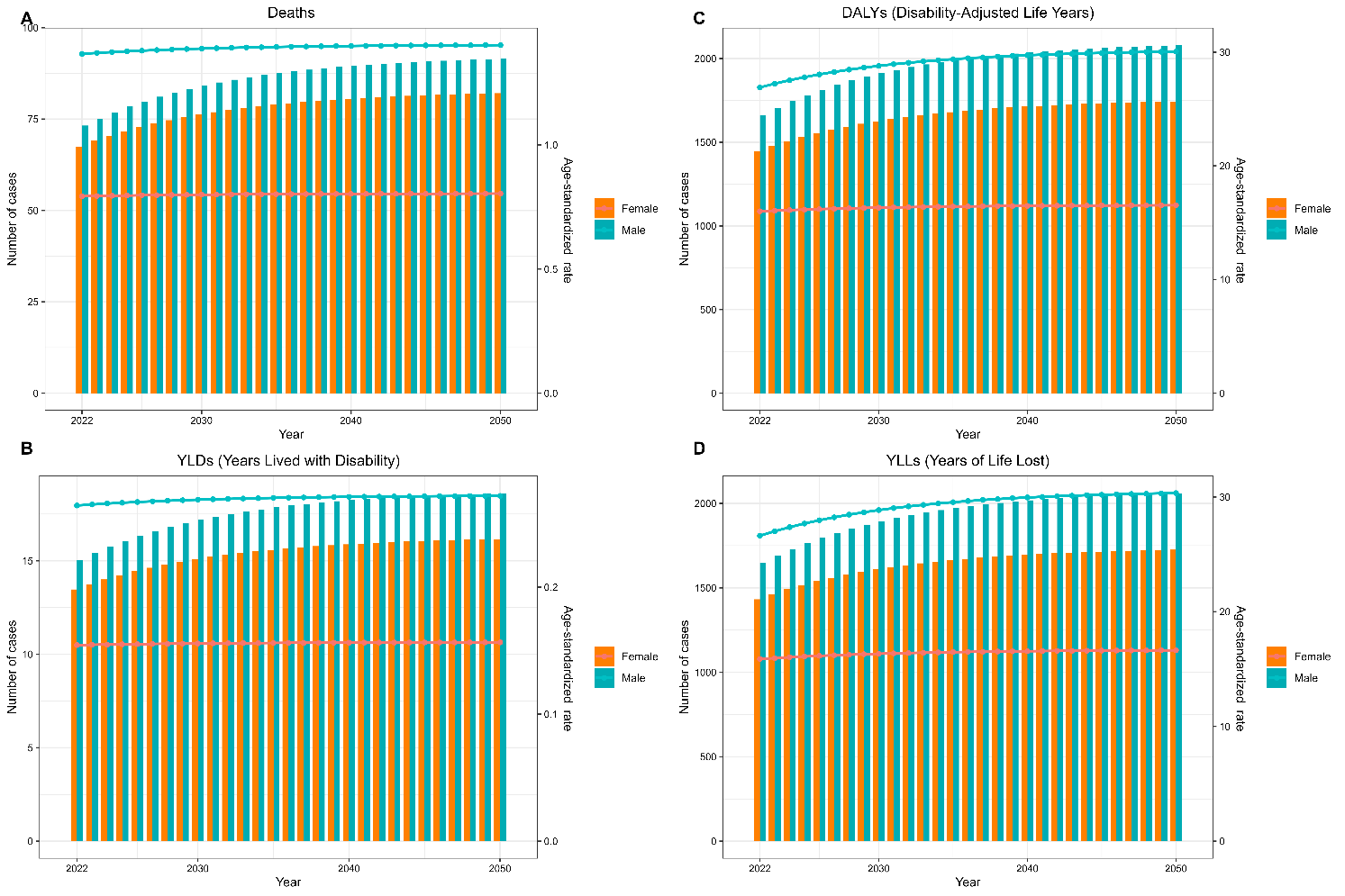
Supplementary Figure 44.** Projections to 2050 of the global burden of liver cancer attributable to high fasting plasma glucose (HFPG) in South Africa performed using the Exponential Smoothing (ES) Model.

# Supplementary Tables

**Table S1** The deaths and ASMR of liver cancer attributable to HFPG in BRICS countries in 1990 and 2021.

|  | 1990 | | 2021 | | EAPC (95% *CI*) |
| --- | --- | --- | --- | --- | --- |
|  | number (95% UI) | ASR (95% UI) | number (95% UI) | ASR (95% UI) |  |
| **Brazil** | 35.84 (3.51-75.58) | 0.13 (0.01-0.28) | 154.26 (16.31-310.92) | 0.19 (0.02-0.37) | 2.25 (1.94-2.57) |
| sex |  |  |  |  |  |
| Female | 22.26 (2.14-47.35) | 0.15 (0.01-0.32) | 84.25 (8.76-170.56) | 0.18 (0.02-0.37) | 1.35 (1.02-1.67) |
| Male | 13.58 (1.36-28.87) | 0.11 (0.01-0.23) | 70.01 (7.57-143.18) | 0.19 (0.02-0.39) | 2.81 (2.49-3.12) |
| age |  |  |  |  |  |
| 40-44 years | 0.5 (0.05-1.02) | 0.01 (0-0.01) | 1.42 (0.15-3.07) | 0.01 (0-0.02) | 1.62 (1.37-1.86) |
| 45-49 years | 1 (0.1-2.17) | 0.02 (0-0.04) | 2.88 (0.3-6.12) | 0.02 (0-0.04) | 1.7 (1.43-1.98) |
| 50-54 years | 2.04 (0.19-4.64) | 0.04 (0-0.09) | 6.95 (0.76-14.72) | 0.05 (0.01-0.11) | 1.99 (1.68-2.3) |
| 55-59 years | 3.09 (0.34-6.65) | 0.07 (0.01-0.16) | 13.37 (1.73-26.99) | 0.11 (0.01-0.23) | 2.36 (2.05-2.67) |
| 60-64 years | 5.04 (0.52-11.02) | 0.14 (0.01-0.31) | 21.06 (2.47-44.24) | 0.21 (0.03-0.45) | 2.37 (2.07-2.67) |
| 65-69 years | 5.69 (0.57-12.17) | 0.21 (0.02-0.45) | 25.2 (2.71-51.65) | 0.33 (0.04-0.67) | 2.23 (1.92-2.53) |
| 70-74 years | 6.25 (0.59-12.94) | 0.33 (0.03-0.68) | 25.82 (2.51-49.43) | 0.45 (0.04-0.86) | 1.94 (1.6-2.28) |
| 75-79 years | 6.06 (0.58-11.91) | 0.47 (0.05-0.93) | 23.53 (2.35-44.86) | 0.62 (0.06-1.18) | 1.69 (1.32-2.05) |
| 80-84 years | 4.17 (0.38-8.58) | 0.62 (0.06-1.27) | 18.73 (1.89-37.57) | 0.76 (0.08-1.53) | 1.51 (1.11-1.91) |
| 85-89 years | 1.56 (0.15-3.47) | 0.53 (0.05-1.19) | 10.15 (0.97-20.84) | 0.78 (0.07-1.61) | 1.95 (1.57-2.33) |
| 90-94 years | 0.36 (0.03-0.84) | 0.44 (0.04-1.01) | 4.06 (0.38-8.85) | 0.73 (0.07-1.59) | 2.14 (1.72-2.56) |
| 95+ years | 0.07 (0.01-0.16) | 0.36 (0.03-0.86) | 1.09 (0.1-2.57) | 0.51 (0.05-1.21) | 1.72 (1.15-2.31) |
| **China** | 1233.37 (123.85-2656.82) | 0.46 (0.05-1) | 3419.97 (358.94-7296.94) | 0.49 (0.05-1.04) | 0.99 (0.69-1.29) |
| sex |  |  |  |  |  |
| Female | 619.73 (64.57-1357.05) | 0.45 (0.05-0.99) | 1615.63 (162.44-3455.36) | 0.43 (0.04-0.93) | 0.58 (0.28-0.89) |
| Male | 613.64 (58.45-1328.11) | 0.48 (0.05-1.03) | 1804.34 (189.5-4029.7) | 0.55 (0.06-1.23) | 1.08 (0.76-1.4) |
| age |  |  |  |  |  |
| 40-44 years | 40.42 (3.62-91.2) | 0.06 (0.01-0.14) | 53.25 (5.4-114.49) | 0.06 (0.01-0.13) | -0.34 (-0.57--0.12) |
| 45-49 years | 65.78 (5.54-135.72) | 0.13 (0.01-0.26) | 128.36 (12.44-277.88) | 0.12 (0.01-0.25) | -0.07 (-0.45-0.32) |
| 50-54 years | 106.23 (10.02-236.96) | 0.22 (0.02-0.5) | 241.89 (25.35-534.24) | 0.2 (0.02-0.44) | 0.25 (-0.1-0.6) |
| 55-59 years | 161.54 (17.57-362.75) | 0.37 (0.04-0.84) | 328.95 (37.53-732.32) | 0.3 (0.03-0.67) | -0.36 (-0.64--0.08) |
| 60-64 years | 205.06 (22.68-441) | 0.58 (0.06-1.25) | 379 (45.97-819.85) | 0.52 (0.06-1.12) | 0.24 (-0.03-0.51) |
| 65-69 years | 218.97 (21.54-462.89) | 0.8 (0.08-1.7) | 599.6 (63.01-1252.34) | 0.78 (0.08-1.63) | 0.53 (0.15-0.91) |
| 70-74 years | 195.66 (19.11-414.35) | 1.04 (0.1-2.2) | 553.64 (55.91-1150.13) | 1.04 (0.1-2.16) | 0.22 (-0.12-0.57) |
| 75-79 years | 138.37 (13.91-298.45) | 1.22 (0.12-2.62) | 449.33 (46.05-934.12) | 1.36 (0.14-2.82) | 0.85 (0.46-1.24) |
| 80-84 years | 64.5 (6.32-134.39) | 1.22 (0.12-2.54) | 388.88 (39.9-820.95) | 1.96 (0.2-4.15) | 3.25 (2.66-3.84) |
| 85-89 years | 31.11 (2.99-66.75) | 1.84 (0.18-3.96) | 217 (19.77-478.7) | 2.28 (0.21-5.03) | 1.73 (1.28-2.19) |
| 90-94 years | 5.43 (0.53-11.72) | 1.77 (0.17-3.82) | 70.53 (6.71-159.9) | 2.41 (0.23-5.45) | 1.59 (1.16-2.02) |
| 95+ years | 0.29 (0.03-0.64) | 0.73 (0.06-1.59) | 9.54 (0.89-22.02) | 1.49 (0.14-3.45) | 3.66 (3.15-4.17) |
| **India** | 246.77 (25.2-522.67) | 0.18 (0.02-0.38) | 1521.76 (163.96-3096.2) | 0.41 (0.04-0.83) | 2.86 (2.56-3.16) |
| sex |  |  |  |  |  |
| Female | 99.75 (9.83-218.3) | 0.15 (0.01-0.33) | 724.41 (76.53-1497.48) | 0.37 (0.04-0.77) | 2.9 (2.8-3.01) |
| Male | 147.01 (15.07-310.51) | 0.2 (0.02-0.43) | 797.35 (85.26-1636.25) | 0.44 (0.05-0.91) | 2.5 (2.32-2.69) |
| age |  |  |  |  |  |
| 40-44 years | 3.44 (0.34-7.18) | 0.01 (0-0.02) | 15.03 (1.59-31.41) | 0.02 (0-0.03) | 2.39 (2.26-2.52) |
| 45-49 years | 7.71 (0.78-16.44) | 0.02 (0-0.05) | 33.28 (3.61-71.71) | 0.04 (0-0.09) | 2.17 (2.06-2.28) |
| 50-54 years | 16.04 (1.53-34.23) | 0.05 (0.01-0.12) | 67.8 (7.4-140.69) | 0.1 (0.01-0.21) | 1.99 (1.82-2.17) |
| 55-59 years | 30.34 (3.26-64.59) | 0.12 (0.01-0.26) | 140.2 (17.33-296.7) | 0.25 (0.03-0.53) | 2.28 (2.07-2.49) |
| 60-64 years | 44.3 (4.91-96.25) | 0.22 (0.02-0.48) | 210.26 (24.12-448.31) | 0.44 (0.05-0.94) | 2.11 (1.99-2.23) |
| 65-69 years | 46.35 (4.78-97.62) | 0.34 (0.03-0.71) | 285.75 (31.75-581.32) | 0.75 (0.08-1.53) | 2.29 (2.11-2.47) |
| 70-74 years | 41.88 (4.17-88.12) | 0.48 (0.05-1.01) | 284.38 (29.7-567.06) | 1.05 (0.11-2.09) | 2.45 (2.34-2.56) |
| 75-79 years | 32.12 (3.1-66.7) | 0.62 (0.06-1.3) | 231.69 (23.42-451.96) | 1.37 (0.14-2.67) | 2.64 (2.47-2.8) |
| 80-84 years | 19.28 (1.83-39.94) | 0.68 (0.06-1.41) | 165.68 (16.38-328.35) | 1.7 (0.17-3.37) | 3.35 (2.93-3.77) |
| 85-89 years | 4.73 (0.44-10.26) | 0.48 (0.04-1.04) | 67.04 (6.74-134.29) | 1.6 (0.16-3.2) | 4.46 (3.7-5.23) |
| 90-94 years | 0.56 (0.05-1.28) | 0.23 (0.02-0.52) | 18.89 (1.78-40.44) | 1.5 (0.14-3.22) | 6.32 (5.14-7.52) |
| 95+ years | 0.03 (0-0.07) | 0.06 (0.01-0.14) | 1.77 (0.17-3.97) | 0.65 (0.06-1.45) | 7.83 (6.44-9.24) |
| **Russian Federation** | 34.29 (3.41-74.73) | 0.06 (0.01-0.13) | 153.41 (14.85-327) | 0.19 (0.02-0.4) | 4.99 (4.55-5.42) |
| sex |  |  |  |  |  |
| Female | 19.78 (1.97-42.57) | 0.05 (0-0.1) | 86.92 (8.63-181.33) | 0.16 (0.02-0.34) | 4.89 (4.26-5.52) |
| Male | 14.51 (1.4-32.23) | 0.08 (0.01-0.17) | 66.49 (6.17-148.16) | 0.23 (0.02-0.51) | 4.35 (3.8-4.9) |
| age |  |  |  |  |  |
| 40-44 years | 0.29 (0.03-0.61) | 0 (0-0.01) | 0.79 (0.08-1.73) | 0.01 (0-0.02) | 3.67 (2.99-4.35) |
| 45-49 years | 0.5 (0.05-1.15) | 0.01 (0-0.02) | 1.98 (0.17-4.38) | 0.02 (0-0.04) | 3.82 (3.21-4.44) |
| 50-54 years | 1.97 (0.19-4.41) | 0.02 (0-0.04) | 4.17 (0.4-9.34) | 0.05 (0-0.11) | 3.96 (3.43-4.49) |
| 55-59 years | 3.18 (0.33-7.03) | 0.04 (0-0.09) | 10.76 (1.11-23.44) | 0.11 (0.01-0.25) | 3.95 (3.43-4.48) |
| 60-64 years | 6.33 (0.7-13.82) | 0.07 (0.01-0.16) | 19.9 (2.08-44.24) | 0.19 (0.02-0.43) | 4.11 (3.6-4.62) |
| 65-69 years | 5.39 (0.57-11.89) | 0.1 (0.01-0.23) | 27.47 (2.8-58.93) | 0.33 (0.03-0.7) | 4.45 (3.88-5.02) |
| 70-74 years | 4.78 (0.45-10.17) | 0.14 (0.01-0.3) | 22.39 (2.15-45.41) | 0.35 (0.03-0.71) | 4.37 (3.71-5.02) |
| 75-79 years | 6.3 (0.58-13.39) | 0.18 (0.02-0.39) | 17.78 (1.68-36.51) | 0.7 (0.07-1.44) | 5.38 (4.77-6) |
| 80-84 years | 3.68 (0.34-8.03) | 0.2 (0.02-0.44) | 28.16 (2.64-58.77) | 0.88 (0.08-1.84) | 5.81 (5.12-6.51) |
| 85-89 years | 1.42 (0.13-3.17) | 0.19 (0.02-0.44) | 11.98 (1.06-26.03) | 0.87 (0.08-1.88) | 6.12 (5.49-6.76) |
| 90-94 years | 0.37 (0.03-0.88) | 0.22 (0.02-0.52) | 7.04 (0.59-15.85) | 1.07 (0.09-2.42) | 5.84 (5.22-6.47) |
| 95+ years | 0.07 (0.01-0.18) | 0.22 (0.02-0.52) | 0.99 (0.08-2.36) | 0.72 (0.06-1.71) | 3.83 (3.04-4.63) |
| **South Africa** | 22.14 (1.69-51.47) | 0.37 (0.03-0.86) | 136.83 (12.6-289.1) | 1 (0.09-2.12) | 3.08 (2.53-3.63) |
| sex |  |  |  |  |  |
| Female | 14.13 (1.06-34.39) | 0.39 (0.03-0.95) | 65.78 (6.31-138.09) | 0.79 (0.08-1.67) | 2.23 (1.78-2.69) |
| Male | 8.01 (0.54-21.57) | 0.34 (0.02-0.9) | 71.05 (6.35-156.06) | 1.36 (0.12-2.99) | 4.08 (3.18-4.99) |
| age |  |  |  |  |  |
| 40-44 years | 0.44 (0.04-0.93) | 0.03 (0-0.05) | 1.62 (0.16-3.71) | 0.04 (0-0.1) | 1.44 (0.75-2.15) |
| 45-49 years | 0.62 (0.06-1.42) | 0.04 (0-0.1) | 2.55 (0.25-5.75) | 0.08 (0.01-0.18) | 1.92 (1.55-2.3) |
| 50-54 years | 0.98 (0.09-2.31) | 0.08 (0.01-0.2) | 5.73 (0.56-13.38) | 0.22 (0.02-0.51) | 3.08 (2.5-3.67) |
| 55-59 years | 1.55 (0.15-3.57) | 0.16 (0.02-0.38) | 11.98 (1.19-25.21) | 0.52 (0.05-1.1) | 3.69 (3.04-4.35) |
| 60-64 years | 1.98 (0.17-4.7) | 0.24 (0.02-0.58) | 18.09 (1.8-38.99) | 0.94 (0.09-2.02) | 4.56 (3.83-5.28) |
| 65-69 years | 2.69 (0.2-6.31) | 0.44 (0.03-1.04) | 22.98 (2.11-47.77) | 1.58 (0.15-3.29) | 4.22 (3.44-5.01) |
| 70-74 years | 3.15 (0.2-7.54) | 0.7 (0.05-1.68) | 20.71 (1.84-42.93) | 2.09 (0.19-4.33) | 3.62 (2.85-4.4) |
| 75-79 years | 3.17 (0.23-7.29) | 1.21 (0.09-2.79) | 18.58 (1.61-38.82) | 3.06 (0.27-6.4) | 2.56 (1.89-3.23) |
| 80-84 years | 4.11 (0.3-9.17) | 2.34 (0.17-5.22) | 19.18 (1.82-39.16) | 5.02 (0.48-10.26) | 1.97 (1.39-2.55) |
| 85-89 years | 2.54 (0.19-6.04) | 2.78 (0.2-6.61) | 11.29 (0.94-23.88) | 6.48 (0.54-13.71) | 2.12 (1.52-2.72) |
| 90-94 years | 0.8 (0.06-1.94) | 2.65 (0.21-6.46) | 3.66 (0.29-8.34) | 7.09 (0.57-16.14) | 2.79 (2.27-3.31) |
| 95+ years | 0.1 (0.01-0.27) | 1.58 (0.12-4.08) | 0.47 (0.04-1.16) | 5.97 (0.5-14.83) | 4.3 (4.04-4.57) |

**Table S2** The DALYs and age-standardized DALYs rate (ASDR) of liver cancer attributable to HFPG in BRICS countries in 1990 and 2021.

|  | 1990 | | 2021 | | EAPC (95% *CI*) |
| --- | --- | --- | --- | --- | --- |
|  | number (95% UI) | ASR (95% UI) | number (95% UI) | ASR (95% UI) |  |
| **Brazil** | 833.28 (82.62-1772.41) | 2.83 (0.28-6.01) | 3431.14 (372.79-6957.22) | 4.04 (0.44-8.18) | 2.23 (1.97-2.49) |
| sex |  |  |  |  |  |
| Female | 504.96 (49.07-1083.37) | 3.23 (0.31-6.92) | 1814.17 (193.94-3682.65) | 3.89 (0.42-7.89) | 1.41 (1.1-1.72) |
| Male | 328.32 (33.1-705.25) | 2.36 (0.24-5.05) | 1616.97 (179.46-3325.72) | 4.21 (0.46-8.65) | 2.82 (2.52-3.12) |
| age |  |  |  |  |  |
| 40-44 years | 24.26 (2.31-49.17) | 0.31 (0.03-0.63) | 68.26 (7.15-148.1) | 0.41 (0.04-0.9) | 1.61 (1.37-1.86) |
| 45-49 years | 43.28 (4.36-93.98) | 0.71 (0.07-1.53) | 124.52 (12.95-264.61) | 0.88 (0.09-1.86) | 1.7 (1.42-1.97) |
| 50-54 years | 78.66 (7.4-178.73) | 1.53 (0.14-3.47) | 267.43 (29.11-566.52) | 2.07 (0.23-4.39) | 1.98 (1.67-2.29) |
| 55-59 years | 104.2 (11.41-224.59) | 2.44 (0.27-5.26) | 451.8 (58.32-912.16) | 3.86 (0.5-7.78) | 2.36 (2.05-2.67) |
| 60-64 years | 146.61 (15.06-320.82) | 4.1 (0.42-8.96) | 613.78 (71.93-1289.19) | 6.25 (0.73-13.12) | 2.37 (2.07-2.67) |
| 65-69 years | 139.8 (14.09-299.11) | 5.22 (0.53-11.16) | 619.36 (66.55-1270.59) | 8.04 (0.86-16.49) | 2.23 (1.92-2.53) |
| 70-74 years | 126.18 (11.87-261.28) | 6.65 (0.63-13.77) | 522.1 (50.9-998.98) | 9.09 (0.89-17.39) | 1.94 (1.6-2.27) |
| 75-79 years | 98.02 (9.44-192.56) | 7.62 (0.73-14.97) | 380.51 (38.07-724.96) | 10.04 (1-19.13) | 1.68 (1.32-2.05) |
| 80-84 years | 52.78 (4.82-108.48) | 7.84 (0.72-16.12) | 237 (23.9-474.44) | 9.63 (0.97-19.28) | 1.5 (1.1-1.9) |
| 85-89 years | 15.75 (1.49-35.05) | 5.39 (0.51-11.99) | 102.02 (9.73-209.35) | 7.87 (0.75-16.15) | 1.93 (1.55-2.31) |
| 90-94 years | 3.18 (0.3-7.33) | 3.83 (0.36-8.81) | 35.51 (3.34-77.62) | 6.36 (0.6-13.91) | 2.13 (1.71-2.55) |
| 95+ years | 0.55 (0.05-1.32) | 2.97 (0.28-7.06) | 8.85 (0.83-20.7) | 4.17 (0.39-9.75) | 1.66 (1.06-2.26) |
| **China** | 33022.08 (3304.07-71427.43) | 11.27 (1.13-24.32) | 80498.17 (8568.31-172563.99) | 11.03 (1.17-23.67) | 0.6 (0.38-0.81) |
| sex |  |  |  |  |  |
| Female | 15776.21 (1654.23-34782.3) | 10.77 (1.12-23.72) | 35632.65 (3645.93-76035.07) | 9.41 (0.96-20.13) | 0.17 (-0.1-0.45) |
| Male | 17245.88 (1630.32-37464.57) | 11.74 (1.11-25.41) | 44865.52 (4772.88-100238.56) | 12.74 (1.35-28.48) | 0.78 (0.5-1.07) |
| age |  |  |  |  |  |
| 40-44 years | 1946.35 (174.05-4390.16) | 2.9 (0.26-6.54) | 2563.55 (260.07-5516.61) | 2.8 (0.28-6.03) | -0.35 (-0.57--0.13) |
| 45-49 years | 2841.22 (238.78-5864.13) | 5.5 (0.46-11.36) | 5538.2 (537.41-11980.7) | 5.02 (0.49-10.86) | -0.07 (-0.45-0.32) |
| 50-54 years | 4075.17 (384.38-9094.05) | 8.54 (0.81-19.06) | 9297.36 (975.11-20535.17) | 7.69 (0.81-16.99) | 0.26 (-0.09-0.61) |
| 55-59 years | 5453.21 (592.43-12232) | 12.57 (1.37-28.2) | 11134 (1271.76-24786.55) | 10.13 (1.16-22.55) | -0.36 (-0.64--0.07) |
| 60-64 years | 5967.72 (660.23-12841.42) | 16.89 (1.87-36.34) | 11014.36 (1337.46-23837.33) | 15.09 (1.83-32.65) | 0.24 (-0.03-0.5) |
| 65-69 years | 5363.14 (527.27-11315.16) | 19.66 (1.93-41.48) | 14688.07 (1544.4-30693.48) | 19.15 (2.01-40.02) | 0.54 (0.15-0.93) |
| 70-74 years | 3947.33 (385.71-8356.93) | 20.98 (2.05-44.41) | 11194.91 (1129.85-23256.32) | 21 (2.12-43.64) | 0.22 (-0.12-0.57) |
| 75-79 years | 2242.65 (225.58-4838.04) | 19.71 (1.98-42.51) | 7261.38 (742.93-15130.33) | 21.93 (2.24-45.68) | 0.83 (0.44-1.22) |
| 80-84 years | 819.52 (80.41-1709.38) | 15.47 (1.52-32.27) | 4917.47 (503.47-10404.53) | 24.85 (2.54-52.57) | 3.23 (2.65-3.82) |
| 85-89 years | 315.57 (30.33-677.65) | 18.71 (1.8-40.17) | 2191.04 (199.5-4836.24) | 23 (2.09-50.77) | 1.73 (1.28-2.18) |
| 90-94 years | 47.75 (4.7-103.12) | 15.56 (1.53-33.61) | 619.12 (58.95-1404.58) | 21.12 (2.01-47.91) | 1.59 (1.16-2.02) |
| 95+ years | 2.46 (0.21-5.39) | 6.08 (0.53-13.3) | 78.73 (7.38-182.15) | 12.32 (1.16-28.5) | 3.63 (3.11-4.15) |
| **India** | 6235.57 (642.55-13247.44) | 4.04 (0.41-8.57) | 35113.54 (3861.39-72104.35) | 8.78 (0.96-17.98) | 2.59 (2.39-2.78) |
| sex |  |  |  |  |  |
| Female | 2411.44 (241.24-5310.93) | 3.32 (0.33-7.28) | 16029.03 (1729.51-33417.63) | 7.82 (0.84-16.27) | 2.74 (2.65-2.83) |
| Male | 3824.12 (394.97-8123.44) | 4.71 (0.48-9.95) | 19084.51 (2074.18-39535.17) | 9.78 (1.06-20.19) | 2.29 (2.14-2.43) |
| age |  |  |  |  |  |
| 40-44 years | 165.32 (16.32-344.89) | 0.38 (0.04-0.8) | 723.09 (76.36-1511.58) | 0.79 (0.08-1.65) | 2.39 (2.26-2.52) |
| 45-49 years | 333.54 (33.73-710.56) | 0.93 (0.09-1.97) | 1440.29 (156.14-3104.82) | 1.83 (0.2-3.94) | 2.17 (2.06-2.28) |
| 50-54 years | 616.53 (58.88-1316.59) | 2.09 (0.2-4.47) | 2605.92 (284.6-5408.73) | 3.87 (0.42-8.03) | 1.99 (1.82-2.16) |
| 55-59 years | 1023.39 (109.74-2176.51) | 4.08 (0.44-8.69) | 4732.85 (585.49-10014.28) | 8.46 (1.05-17.91) | 2.29 (2.08-2.5) |
| 60-64 years | 1290.09 (142.92-2804.72) | 6.47 (0.72-14.06) | 6124.1 (703.71-13063.55) | 12.86 (1.48-27.43) | 2.11 (1.99-2.22) |
| 65-69 years | 1139.12 (117.7-2400.66) | 8.34 (0.86-17.58) | 7006.02 (779.26-14269.52) | 18.48 (2.06-37.64) | 2.28 (2.1-2.47) |
| 70-74 years | 848.57 (84.6-1787.31) | 9.69 (0.97-20.41) | 5758.01 (602.09-11490.64) | 21.18 (2.21-42.27) | 2.45 (2.34-2.56) |
| 75-79 years | 520.21 (50.3-1081.64) | 10.11 (0.98-21.01) | 3758.52 (380.15-7327.75) | 22.19 (2.24-43.26) | 2.62 (2.46-2.78) |
| 80-84 years | 245.73 (23.35-508.95) | 8.7 (0.83-18.02) | 2106.13 (208.33-4167.78) | 21.63 (2.14-42.8) | 3.34 (2.93-3.76) |
| 85-89 years | 47.92 (4.5-103.88) | 4.85 (0.46-10.52) | 678.24 (68.25-1359.33) | 16.19 (1.63-32.44) | 4.46 (3.7-5.23) |
| 90-94 years | 4.91 (0.48-11.17) | 1.99 (0.19-4.54) | 165.65 (15.59-353.3) | 13.17 (1.24-28.1) | 6.33 (5.14-7.52) |
| 95+ years | 0.24 (0.02-0.56) | 0.5 (0.05-1.15) | 14.73 (1.44-33.07) | 5.37 (0.52-12.06) | 7.84 (6.43-9.27) |
| **Russian Federation** | 798.38 (80.64-1745.25) | 1.29 (0.13-2.82) | 3186.6 (314.06-6828.15) | 3.92 (0.39-8.41) | 4.62 (4.2-5.05) |
| sex |  |  |  |  |  |
| Female | 431.55 (43.91-928.52) | 1.08 (0.11-2.32) | 1648.65 (168.38-3435.18) | 3.22 (0.33-6.72) | 4.5 (3.9-5.11) |
| Male | 366.83 (35.63-818.14) | 1.72 (0.16-3.83) | 1537.95 (144.99-3435.26) | 4.97 (0.46-11.07) | 4.3 (3.77-4.84) |
| age |  |  |  |  |  |
| 40-44 years | 13.93 (1.26-29.52) | 0.15 (0.01-0.31) | 37.99 (3.69-83.03) | 0.35 (0.03-0.76) | 3.67 (2.99-4.35) |
| 45-49 years | 21.49 (1.95-49.29) | 0.32 (0.03-0.74) | 85.83 (7.52-189.43) | 0.86 (0.07-1.89) | 3.82 (3.21-4.44) |
| 50-54 years | 75.94 (7.47-169.84) | 0.72 (0.07-1.62) | 160.53 (15.33-359.98) | 1.82 (0.17-4.08) | 3.96 (3.44-4.48) |
| 55-59 years | 106.66 (10.96-236.04) | 1.34 (0.14-2.96) | 362.12 (37.44-787.79) | 3.83 (0.4-8.34) | 3.95 (3.43-4.48) |
| 60-64 years | 184.31 (20.33-402.86) | 2.1 (0.23-4.58) | 579.51 (60.65-1288.54) | 5.62 (0.59-12.5) | 4.12 (3.6-4.65) |
| 65-69 years | 133.23 (14.18-294.14) | 2.59 (0.28-5.72) | 675.82 (69-1450.97) | 8.01 (0.82-17.19) | 4.45 (3.88-5.02) |
| 70-74 years | 96.3 (9.09-204.57) | 2.8 (0.26-5.95) | 453.49 (43.62-919.68) | 7.07 (0.68-14.35) | 4.36 (3.72-5.01) |
| 75-79 years | 101.57 (9.43-215.7) | 2.93 (0.27-6.23) | 285.13 (26.92-586.53) | 11.26 (1.06-23.16) | 5.35 (4.75-5.95) |
| 80-84 years | 46.72 (4.33-102.01) | 2.56 (0.24-5.6) | 355.48 (33.33-741.01) | 11.16 (1.05-23.26) | 5.82 (5.13-6.51) |
| 85-89 years | 14.34 (1.28-32.1) | 1.97 (0.18-4.41) | 120.96 (10.75-262.9) | 8.74 (0.78-19) | 6.11 (5.47-6.75) |
| 90-94 years | 3.27 (0.29-7.69) | 1.94 (0.17-4.57) | 61.65 (5.14-138.97) | 9.39 (0.78-21.18) | 5.84 (5.22-6.47) |
| 95+ years | 0.62 (0.05-1.48) | 1.81 (0.16-4.35) | 8.1 (0.68-19.31) | 5.85 (0.49-13.95) | 3.76 (2.97-4.56) |
| **South Africa** | 462.19 (36.5-1073.46) | 7.06 (0.55-16.42) | 3022.95 (283.57-6434.31) | 20.07 (1.87-42.64) | 3.32 (2.83-3.81) |
| sex |  |  |  |  |  |
| Female | 289.62 (22.12-700.19) | 7.57 (0.58-18.34) | 1410.93 (138.28-2980.55) | 15.94 (1.55-33.67) | 2.48 (2.04-2.92) |
| Male | 172.56 (11.94-469.36) | 6.39 (0.43-17.29) | 1612.02 (145.64-3575.55) | 26.53 (2.38-58.53) | 4.23 (3.3-5.17) |
| age |  |  |  |  |  |
| 40-44 years | 21.37 (1.88-44.92) | 1.22 (0.11-2.57) | 77.95 (7.58-179) | 2.06 (0.2-4.74) | 1.44 (0.74-2.15) |
| 45-49 years | 26.97 (2.52-61.43) | 1.95 (0.18-4.43) | 110.41 (10.64-249.33) | 3.53 (0.34-7.97) | 1.92 (1.55-2.3) |
| 50-54 years | 37.67 (3.41-88.88) | 3.25 (0.29-7.68) | 220.76 (21.67-515.75) | 8.47 (0.83-19.78) | 3.08 (2.5-3.67) |
| 55-59 years | 52.28 (4.94-120.58) | 5.55 (0.52-12.79) | 404.9 (40.08-852.5) | 17.64 (1.75-37.14) | 3.7 (3.04-4.35) |
| 60-64 years | 57.63 (4.81-136.91) | 7.1 (0.59-16.87) | 527.65 (52.55-1138.09) | 27.37 (2.73-59.04) | 4.56 (3.84-5.29) |
| 65-69 years | 65.89 (4.9-154.43) | 10.86 (0.81-25.45) | 565.91 (52-1177.49) | 38.94 (3.58-81.03) | 4.23 (3.45-5.01) |
| 70-74 years | 64.28 (4.13-153.59) | 14.29 (0.92-34.14) | 420.75 (37.41-871.36) | 42.47 (3.78-87.96) | 3.62 (2.85-4.4) |
| 75-79 years | 51.02 (3.67-117.36) | 19.52 (1.4-44.91) | 301.08 (26.19-628.84) | 49.64 (4.32-103.68) | 2.56 (1.89-3.24) |
| 80-84 years | 51.66 (3.76-115.24) | 29.45 (2.14-65.69) | 243.52 (23.06-497.62) | 63.8 (6.04-130.38) | 1.98 (1.4-2.56) |
| 85-89 years | 25.59 (1.87-60.91) | 28.01 (2.04-66.66) | 113.98 (9.47-241.45) | 65.43 (5.44-138.6) | 2.13 (1.53-2.74) |
| 90-94 years | 6.97 (0.55-17.02) | 23.22 (1.84-56.73) | 32.11 (2.59-73.12) | 62.17 (5.01-141.58) | 2.79 (2.27-3.32) |
| 95+ years | 0.86 (0.07-2.21) | 12.98 (1.02-33.51) | 3.93 (0.33-9.75) | 50.22 (4.21-124.64) | 4.34 (4.08-4.61) |
